# Regulation of micro- and small-exon retention and other splicing processes by GRP20 for flower development

**DOI:** 10.1038/s41477-023-01605-8

**Published:** 2024-01-09

**Authors:** Jun Wang, Xinwei Ma, Yi Hu, Guanhua Feng, Chunce Guo, Xin Zhang, Hong Ma

**Affiliations:** 1 Department of Biology, Eberly College of Science, Huck Institutes of the Life Sciences, Pennsylvania State University, University Park, PA USA; 2 Jiangxi Provincial Key Laboratory for Bamboo Germplasm Resources and Utilization, Forestry College, Jiangxi Agricultural University, Nanchang, China; 3 Department of Chemistry and Department of Biochemistry and Molecular Biology, Eberly College of Science, Pennsylvania State University, University Park, PA USA

**Keywords:** Plant development, Plant molecular biology

## Abstract

Pre-mRNA splicing is crucial for gene expression and depends on the spliceosome and splicing factors. Plant exons have an average size of ~180 nucleotides and typically contain motifs for interactions with spliceosome and splicing factors. Micro exons (<51 nucleotides) are found widely in eukaryotes and in genes for plant development and environmental responses. However, little is known about transcript-specific regulation of splicing in plants and about the regulators for micro exon splicing. Here we report that glycine-rich protein 20 (GRP20) is an RNA-binding protein and required for splicing of ~2,100 genes including those functioning in flower development and/or environmental responses. Specifically, GRP20 is required for micro-exon retention in transcripts of floral homeotic genes; these micro exons are conserved across angiosperms. GRP20 is also important for small-exon (51–100 nucleotides) splicing. In addition, GRP20 is required for flower development. Furthermore, GRP20 binds to poly-purine motifs in micro and small exons and a spliceosome component; both RNA binding and spliceosome interaction are important for flower development and micro-exon retention. Our results provide new insights into the mechanisms of micro-exon retention in flower development.

## Abstract (web summary)

Splicing of transcripts containing micro and small exons requires specific factors. This study shows that GRP20 regulates micro- and small-exon retention of transcripts including floral homeotic genes, whose splicing is crucial for flower development.

## Main

Pre-messenger RNA (mRNA) splicing (hereafter RNA splicing) is one of the most important post-transcriptional processes for eukaryotic gene expression^1^ and is required for plant and animal development^2,3^. Compared with animal development, plant development not only depends heavily on proper environmental conditions, but also is negatively impacted by adverse environments^4,5^. The effects of environmental factors on plant development involve the functions of multiple plant hormones including auxin (indole-3-acetic acid) and abscisic acid (ABA)^4,5^. The hormonal regulation of environmental effects on development is largely controlled by transcription factors^4^, as well as epigenetic processes involving microRNA, DNA methylation and histone methylation^4,5^. RNA splicing is carried out by the spliceosome, a complex of small nuclear ribonucleoproteins^6^ and involves the recognition of GU-AG or AU-AC consensus at the exon–intron boundaries by splicing factors (SRs)^7^. Splicing factors and regulators are important for several plant processes, including flowering time^8^, circadian rhythms^9^, stress response^10^ and plant defence^11^. However, relatively little is known about transcript-specific regulation of splicing for genes that are essential for development. Furthermore, molecular mechanisms of specialized factors for regulation of RNA splicing remain largely unknown.

The exon ends are recognized by SRs to ensure accurate splicing in plants and animals^6,12^. In addition, SRs bind to specific motifs in pre-mRNAs, termed exon splicing enhancers and/or intron splicing enhancers, to initiate spliceosome assembly^13,14^. Exon sizes are less variable than intron sizes, averaging 150 nucleotides in vertebrates and 180 nucleotides in plants^15–18^. Exons with typical sizes and exon splicing enhancers can associate with the spliceosome for efficient splicing^19,20^, whereas shorter exons generally lack sufficient sequence motifs and require additional regulators for accurate splicing^21^. Unusually short exons (<51 nucleotides), called micro exons, are widely found in both plants (for example, >8,000 in ~6,000 *Arabidopsis* genes) and animals (~13,000 in humans)^22,23^. Such micro exons have been found to be essential^15,16,24^; for example, in humans and mice, conserved micro exons have been found in brain-specific transcripts and implicated in neurogenesis and brain functions^22,23^.

In plants, the importance of micro exons in gene functions has been suggested by their identification in 10 diverse species^25^ and by their presence in genes crucial for transcriptional regulation, cell division, stress response, protein modification and metabolism^22,23,25–27^. Specifically, micro exons are generally found in MIKC (MADS domain, I region, K domain, and C-terminal domain)-type MADS-box (an acronym of MCM1, AGAMOUS, DEFICIENS and SRF) genes and AP2 (APETALA2) family members encoding putative transcription factors from analyses of 63 plant species^27^. These genes include all core floral homeotic genes, *AGAMOUS* (*AG*), *APETALA1* (*AP1*), *APETALA2* (*AP2*), *APETALA3* (*AP3*), *PISTILLATA* (*PI*), *SEPALLATA3* (*SEP3*) and *SEPALLATA4* (*SEP4*), and their micro exons are conserved across angiosperms^27^. Micro exons in MADS-box genes encode portions of the K domain^27^, which is important for tetramerization^28,29^. Indeed, the reduced inclusion of such a micro exon in the floral MADS-box gene *SEP3* caused abnormal flower development^28,30^. In addition, AP2 family genes containing conserved micro exons include those in the AP2 subfamily (such as *TARGET OF EAT1* (*TOE1*)) and the ERF subfamily (such as *SMALL ORGAN SIZE1* (*SMOS1*)), which are also required for normal development in *Arabidopsis* and rice^27,31^. Moreover, a conserved nine-nucleotide micro exon encoding a portion of the AP2 domain in *WRI1* (*WRINKLED1*) is crucial to fatty acid synthesis in *Arabidopsis* and plants^27,32^. However, regulators for micro-exon retention have not been reported for any transcripts in plants.

Only a few studies have identified factors regulating micro-exon splicing in animals. In humans and mice, transcriptome analyses of multiple tissues identified >2,500 alternative splicing (AS) events and the micro exons of 3–27 nucleotides affected by AS are highly conserved and potentially regulatory in brain development^22^. The retention of such micro exons was increased in tissue culture expressing the neuronal splicing factor nSR100/SRRM4, suggesting that nSR100 promotes micro-exon inclusion in some mRNAs; this was further supported by the finding that brains of individuals with autism have both reduced nSR100 levels and misregulated splicing of micro exons^22^. The RNA-binding protein RBFOX1 was found to bind to intronic sequences adjacent to 145 brain-specific micro exons, suggesting a role in regulating the inclusion of micro exons; in addition, a poly-pyrimidine-binding protein, PTBP1, was reported to reduce the retention of micro exons^23^. However, the mechanisms of these three RNA-binding proteins in regulating micro-exon splicing remain unclear. Moreover, animal splicing factors for micro exons of 27–50 nucleotides have not been reported. Furthermore, exons slightly larger than micro exons (51–100 nucleotides) are also found in plant and animal genomes (for example, *Arabidopsis*, rice and human)^15,16,18^ and defined as small exons in this study. Splicing of small exons might also be facilitated by additional factors; for example, the N1 exon in the mouse c-*src* gene and the IDX exon in the human *LGH* gene are inefficiently spliced in vitro by reconstituted spliceosomes, whereas artificially extending the N1 exon to 109 nucleotides increased its retention efficiency^20,33^. These observations suggest that the proper splicing of small exons also benefits from additional regulation^33^, but such regulators have not been reported in either plants or animals.

Pre-mRNA sequence characteristics also impact accurate splicing, such as intronic poly-pyrimidine tracts, which are generally required for splicing and recognized by known SRs: SRp40, SF2, SRp55, SC35 and U2AF65 in humans^14^. For micro exons, the aforementioned nSR100, RBFOX1 and PTBP1 proteins are suggested to bind poly-pyrimidine (poly(Y)) motifs in the neighbouring introns to regulate micro-exon splicing for a small fraction (hundreds) of animal genes with micro exons^22,23^. Notably, the replacement in imperfect poly(Y) tracts of a purine by a pyrimidine in the upstream introns improved retention of short internal exons^19^, suggesting the importance of such sequence characteristics in diverse transcripts for normal splicing. However, sequence motifs in the exons and their binding proteins for micro-exon splicing in plants and animals remain unknown.

## Results

### *GRP20* encodes a predicted non-classical RNA-binding protein

**Fig. 1:**
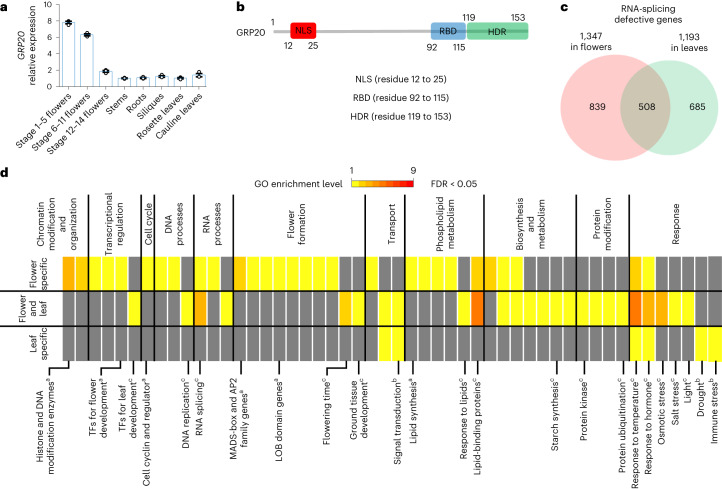
Pre-mRNA splicing events in WT and *grp20*. **a**, Relative expression levels of *GRP20* in several *Arabidopsis* organs and structures: stage 1–5 flowers (including the inflorescence meristem), stage 6–11 flowers, stage 12–14 flowers, stems, roots, siliques, rosette leaves and cauline leaves. GRP20 is generally expressed in multiple tissues. The data are presented as mean ± s.e.m. from three biological replicates. **b**, The GRP20 protein domain structure, with an NLS, an RBD and an HDR. **c**, A Venn diagram of overlap for genes with splicing defects in flowers and/or leaves. **d**, GO category analysis of genes with defective transcripts in flower-specific, flower and leaf overlapping, and leaf-specific groups. The GO categories are shown for those with FDR < 0.05 (Mann–Whitney *U* test with 95% confidence intervals). GO enrichment is ranked 1–9 from yellow to red. Red indicates higher enrichment. ^a^Categories highly enriched in the flower: histone and DNA modification enzymes, transcription factors for flower development, cell-division-related genes, ABCDE floral homeotic genes (MADS-box and AP2 family genes), LOB domain genes and lipid synthesis. ^b^Categories highly enriched in the leaf: genes for signal transduction and for drought and immune stress response. ^c^Categories highly enriched in both the leaf and flower: transcription factors for leaf development, DNA replication, RNA splicing, flowering time, ground tissue development, starch synthesis, lipid binding proteins and protein ubiquitination and those that broadly respond to temperature, hormone, osmotic stress, salt stress and lipids. TF, transcription factor.

**Extended Data Fig. 1:**
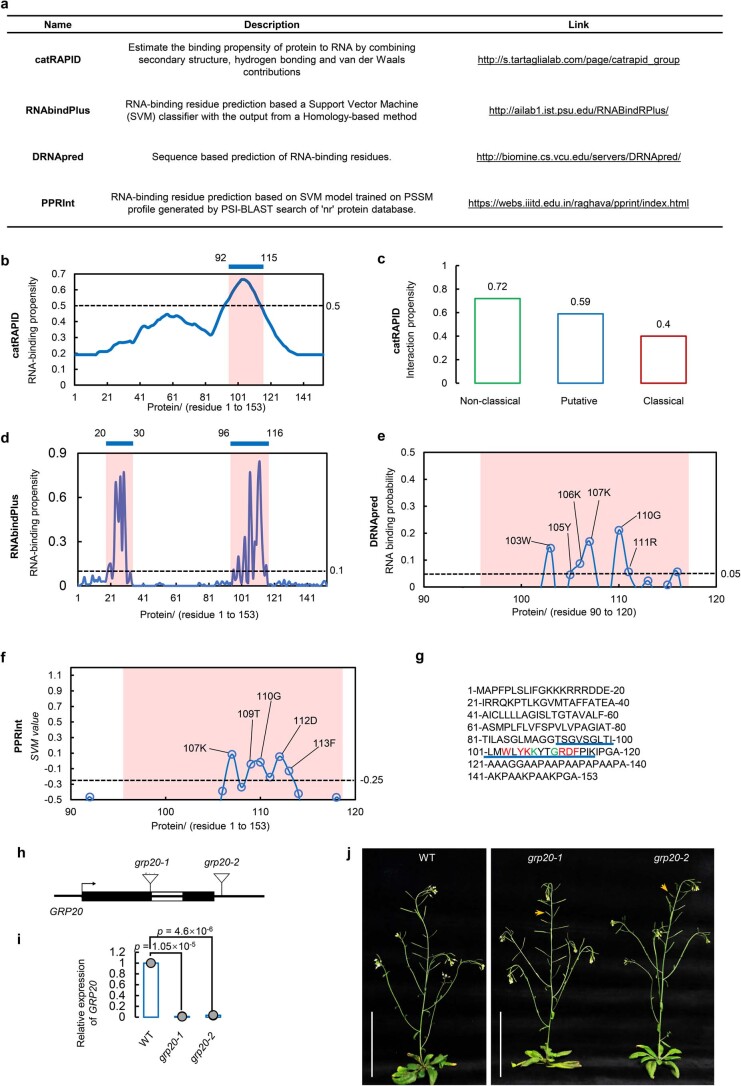
The prediction of an RNA-binding domain and highly disordered domain in GRP20. **a**, The four prediction programs used for the RNA-binding prediction. The basic algorithms are shown in the table. **b**, The RNA binding propensity of GRP20 from catRAPID. The propensity ≥ 0.5 indicates potential RNA-binding ability, identifying the region with residue 92 to 115 as an RNA binding domain (highlighted by pink background). The overall interaction score of GRP20 is 0.54 (≥0.5), indicating that GRP20 is a putative RNA binding protein. **c**, The prediction of classification of the potential RNA-binding domain in GRP20 by catRAPID. The program divides potential RNA-binding proteins into three categories: classical, non-classical and putative according to propensity values^79^. The scores ≥ 0.5 reflects the propensity to be associated with one of the categories. The results indicate that the score of GRP20 is highest (0.72) for a non-classical RNA-binding protein and above threshold (0.59) for a putative RNA-binding protein, but not enough for a classic RNA-binding protein. **d**, RNA-binding propensity of GRP20 predicted by RNAbindPlus. The propensity ≥ 0.1 indicates potential RNA-binding residues in GRP20, in two regions: residue 20 to 30, and residue 96 to 116 (highlighted by pink). **e, f**, The prediction of core residues in GRP20 for RNA binding by DRNApred **(e)** and PPRlnt **(f)** programs. Each dot represents an amino acid. The amino acids with high probability for binding are indicated for the putative RNA-binding region (residue 92 to 116, highlighted by pink). The cutoff used in the two programs are 0.5 and −0.25, respectively. **g**, The GRP20 amino acid sequence, with the putative RNA-binding region (blue underlined) and key amino acids for RNA binding (Red letters: predicted by either predictor in **e** and **f**; Green letters: predicted by both predictors in **e** and **f**). **h**, The *GRP20* gene structure with positions of two T-DNA insertion *grp20* mutations (the triangles above gene structure). **i**, The relative expression of *GRP20* in two *grp20* alleles compared to WT by RT-qPCR. The expression level of *GRP20* is normalized to WT being set as ‘1’. Two-sided Student’s *t* test. Data are presented as mean ± SE from three biological replicates. **j**, Vegetative growth between WT and two *grp20* mutants. Some siliques are shorter, suggesting that the mutation of *GRP20* affected fertility. Yellow arrows indicate short siliques. Bar = 10 cm. Source data

Glycine-rich proteins (GRPs) are important for seed germination, root development, stress response and pollen development^34,35^. Two hnRNP (heterogeneous nuclear ribonucleoproteins)-like GRPs, GRP7 and GRP8, were found to bind to RNAs and affect the AS of nearly 100 transcripts using RT (reverse transcription)-PCR^36^. GRP7 also regulates the splicing of its own pre-mRNA in feedback control associated with the circadian clock^37^ and promotes AS of *FLM* to control flowering time^38^. These observations suggest that other GRPs might function in RNA binding and splicing. Our early transcriptomic analyses identified *GRP20* as expressed in all tissues tested and more highly in flowers (Fig. 1a), suggesting a role in the flower and possibly other processes. Moreover, the similar expression levels in leaves between *GRP20* and a constitutively expressed gene *EF1α* (Supplementary Fig. 1a) suggest that the level of *GRP20* expression in leaves is not very low, just much lower than its level in flowers. To obtain clues about the molecular functions of GRP20, we examined the domain organization of GRP20. GRP20 has a putative nuclear localization signal (NLS, residues 12 to 25; Fig. 1b); however, there were no annotated domains in the C-terminal region. We used the catRAPID and RNAbindPlus programs to investigate whether GRP20 could potentially bind to RNA (Extended Data Fig. 1a). The two programs predicted GRP20 as an RNA-binding protein, with a predicted RNA-binding domain (RBD; residues 92 to 115; Fig. 1b,c and Extended Data Fig. 1b,d), and probably belonging to a non-classical type of RNA-binding protein (Extended Data Fig. 1c). We further used two other programs (DRNApred and PPRInt) to identify likely core amino acid residues for RNA binding (Extended Data Fig. 1a) and identified several aromatic (W/Y) and charged (K/R/D) amino acids as potentially having higher affinities for RNA (Extended Data Fig. 1e–g). Moreover, we annotated a highly disordered region (HDR, residues 119 to 153) in the C-terminal region of GRP20 (Fig. 1b). The relatively high floral expression and multiple predicted protein domains (Supplementary Fig. 1b–d), as well as preliminary mutant phenotypes, suggested that GRP20 is an excellent candidate for functional investigation.

### GRP20 regulates splicing of genes for development and response

The putative RNA-binding domain suggests that GRP20 might regulate RNA processing. To test a possible role of GRP20 in RNA splicing, we used two T-DNA (transfer-DNA of the Ti plasmid of *Agrobacterium tumefaciens*) insertional *grp20* alleles (Extended Data Fig. 1h,j) with greatly reduced *GRP20* expression (Extended Data Fig. 1i). We examined possible effects of *grp20-1* on RNA splicing in flowers and leaves by transcriptomic analyses and identified 839, 508 and 685 genes with various defects in splicing detected only in the flower, in both the flower and leaf, and only in the leaf, respectively (Fig. 1c). For convenience, the most abundant transcript detected in the wild type (WT) is referred to as the ‘typical transcript’, whereas other transcripts are referred to as alternative transcripts (sometimes also called ‘defective transcripts’ if detected only in the *grp20* mutant). Gene Ontology (GO) category analyses revealed that categories for chromatin modification and organization, cell cycle regulation, flower organ formation, phospholipid and pigment synthesis, and response to auxin and heat are highly enriched in defective transcripts found only in flowers (Fig. 1d). In addition, categories of biosynthesis and metabolism, transcriptional regulation, circadian rhythm and RNA metabolism are enriched in defective (alternatively spliced) transcripts detected in both flowers and leaves (Fig. 1d). Furthermore, signal transduction and diverse environmental response including response to temperature (heat and cold), osmotic, salt, light, drought, lipid and immune stresses are enriched in transcripts showing defects only in leaves (Fig. 1d). Therefore, GRP20 is an important splicing regulator of genes that probably play roles in plant growth and environmental responses.

**Fig. 2:**
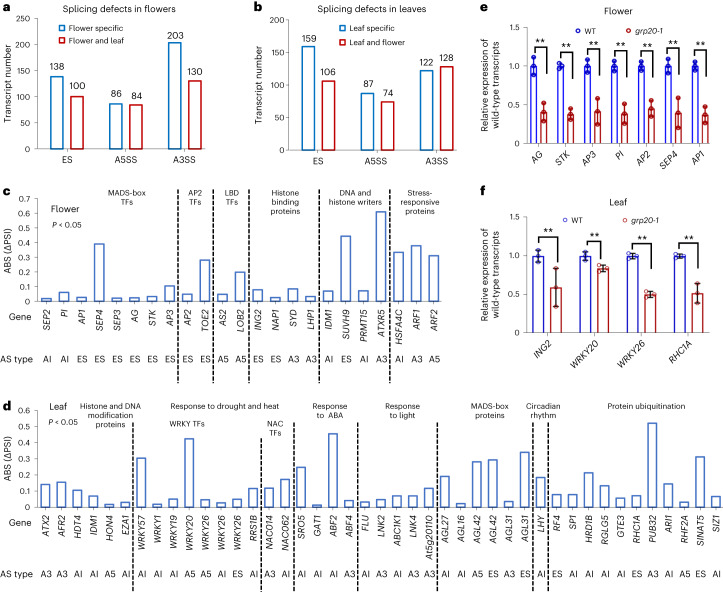
Splicing events in WT and *grp20* flowers and leaves. **a**,**b**, Number of genes whose transcripts had pre-mRNA splicing defects (ES, A3SS and A5SS) in *grp20* flowers (**a**) and leaves (**b**). Flower specific, different transcripts found only in flowers. Flower and leaf, different transcripts found in both flower and leaf. Leaf specific, different transcripts found only in leaves. **c**, ΔPSI of alternative transcripts in *grp20* and WT flowers, for floral homeotic genes (MADS-box genes and AP2), AP2 family genes, LOB domain genes, histone-binding genes, DNA and histone writers, and stress-responsive genes. Data are shown as the absolute value of ΔPSI (ABS (ΔPSI)) in **c** and **d**. *P* values for all transcripts in **c** and **d** are <0.05. The ΔPSI and *P* value of each affected transcript are shown in Supplementary Table 1. ES, A3 (A3SS), A5 (A5SS) and AI (ASI) at the bottom represent the splicing type in the transcripts. Type indicates the corresponding splicing defective category: ES, A3SS, A5SS and ASI. **d**, ΔPSI of alternative transcripts in *grp20* and WT leaves, for histone and DNA modification genes, WRKY and NAC transcription factors, MADS-box genes, circadian rhythm genes, protein ubiquitinating genes and genes that respond to ABA and light. **e**,**f**, Relative levels of wild-type transcripts of skipped exons of floral homeotic genes in *grp20-1* flowers (**e**), and WRKY family genes, histone methylation binding gene *ING2* and protein ubiquitination gene *RHC1A* in *grp20-1* leaves (**f**). *P* (*AG*) = 0.0033; *P* (*STK*) = 0.00018; *P* (*AP3*) = 0.0055; *P* (*PI*) = 0.0017; *P* (*AP2*) = 0.0013; *P* (*SEP4*) = 0.0081; *P* (*AP1*) = 0.00078; *P* (*ING2*) = 0.0031; *P* (*WRKY20*) = 0.0097; *P* (*WRKY26*) = 8.27 × 10^−5^; *P* (*RHC1A*) = 0.00301. The primers are listed in Supplementary Table 7. The data are presented as mean ± s.e.m. from three biological replicates for **e** and **f**. **P < 0.01, two-sided Student’s *t*-test in **e** and **f**. Source data

**Fig. 3:**
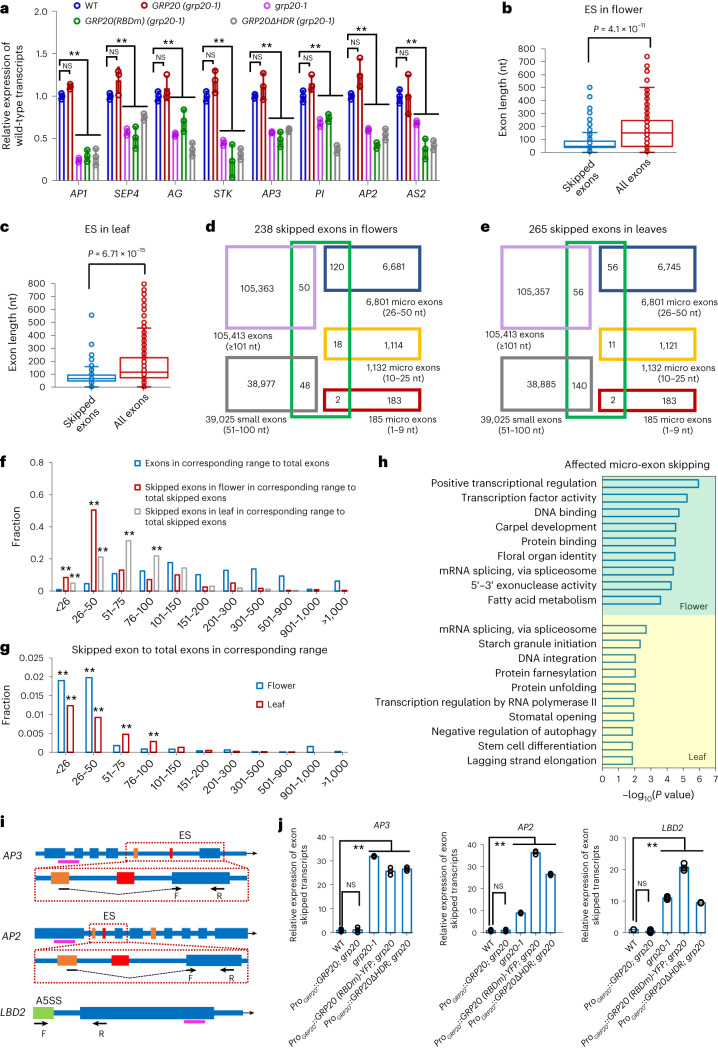
Micro- and small-exon skipping in *grp20* flowers and leaves, and RNA expression of floral homeotic and LOB domain genes. **a**, Relative levels of alternative floral transcripts with affected exons of floral homeotic genes and *AS2* in different genotypes (shown as mean ± s.e. from three biological replicates). The *P* value is shown in the following order (from left to right) in **a** and **j** by two-sided Student’s *t*-test, **indicate *P* < 0.01 and NS indicates not significant: WT versus *GRP20*, WT versus *grp20-1*, WT versus *GRP20* (*RBDm*) and WT versus *GRP20ΔHDR*. For *AP1*, *P* = 0.13, 1.9 × 10^−5^, 0.0005 and 0.0026; for *SEP4*, *P* = 0.16, 0.00017, 0.003 and 0.0013; for *AG*, *P* = 0.37, 0.002, 0.024 and 0.00056; for *STK*, *P* = 0.12, 0.00058, 0.0029 and 0.00017; for *AP3*, *P* = 0.32, 0.0003, 0.0064 and 3.5 × 10^−5^; for *PI*, *P* = 0.09, 0.0039, 0.0081 and 0.00037; for *AP2*, *P* = 0.11, 0.00041, 3.4 × 10^−5^ and 0.0017; for *AS2*, *P* = 0.92, 0.0028, 0.0027 and 0.00086. For primer information, see Extended Data Fig. 3 and Supplementary Table 7. **b**,**c**, The length distribution of skipped exons and all exons in the genes in flowers (**b**) and leaves (**c**), with two-sided Student’s *t*-test. The lower bound, maxima, minima, centre and upper bound of box plots (from left to right) are shown as follows: for skipped exon in **b**: 40, 2,131, 2, 48 and 88.5; for all exons in **b**: 48, 2,131, 2, 151.56 and 247.26; for skipped exon in **c**: 61, 2,757, 5, 83 and 131; for all exons in **c**: 93, 2,757, 5, 169.29 and 286.13. **d**,**e**, Venn diagrams for overlaps for 238 skipped exons in *grp20* flowers (**d**) or 265 skipped exons in *grp20* leaves (**e**), with exons of various size ranges. **f**, The enrichments were indicated by two asterisks as the fraction of skipped exons larger than twice the corresponding referenced exons. Fractions of exon numbers in various size ranges to total exons (reference) and skipped exons in various size ranges to total skipped exons in *grp20* flowers and leaves. **g**, Two asterisks indicate enrichments that the fraction is larger than 0.0025. Fractions of skipped exon numbers to total exons in various size ranges in *grp20* flowers and leaves. **h**, GO category analysis of genes with affected micro-exon skipping in *grp20* flowers and leaves, shown for those with *P* < 0.05 (Mann–Whitney *U* test with 95% confidence intervals). **i**, Illustrations of *AP3*, *AP2* and *LBD2* gene structures with positions of forward (F) and reverse (R) primers for qRT-PCR of specific transcripts. Blue boxes, exons; thin lines, introns; orange boxes, wild-type micro exons; red boxes, skipped micro exons in *AP3* and *AP2*. A green box in *LBD2:* an alternative extra exonic region (A5SS) in *LBD2*. The red dotted boxes below gene structures are enlargements of the affected regions of *AP3* and *AP2*; dashed lines, introns and the skipped micro exons; thick pink line below each gene structure, the region as control for amplifying both wild-type and alternative transcripts. **j**, Relative expression of *AP3* and *AP2* transcripts with micro-exon skipping, or alternative 5′ site in *LBD2*, in flowers by RT-qPCR. For *AP3*, *P* = 0.83, 4.14 × 10^−6^, 0.00041 and 6.4 × 10^−6^; for *AP2*, *P* = 0.9, 3.31 × 10^−5^, 8.01 × 10^−6^ and 3.46 × 10^−7^; for *LBD2*, *P* = 0.22, 0.00083, 0.0011 and 5.49 × 10^−5^. The data are presented as mean ± s.e.m. from three biological replicates for **a** and **j**. Source data

**Extended Data Fig. 2:**
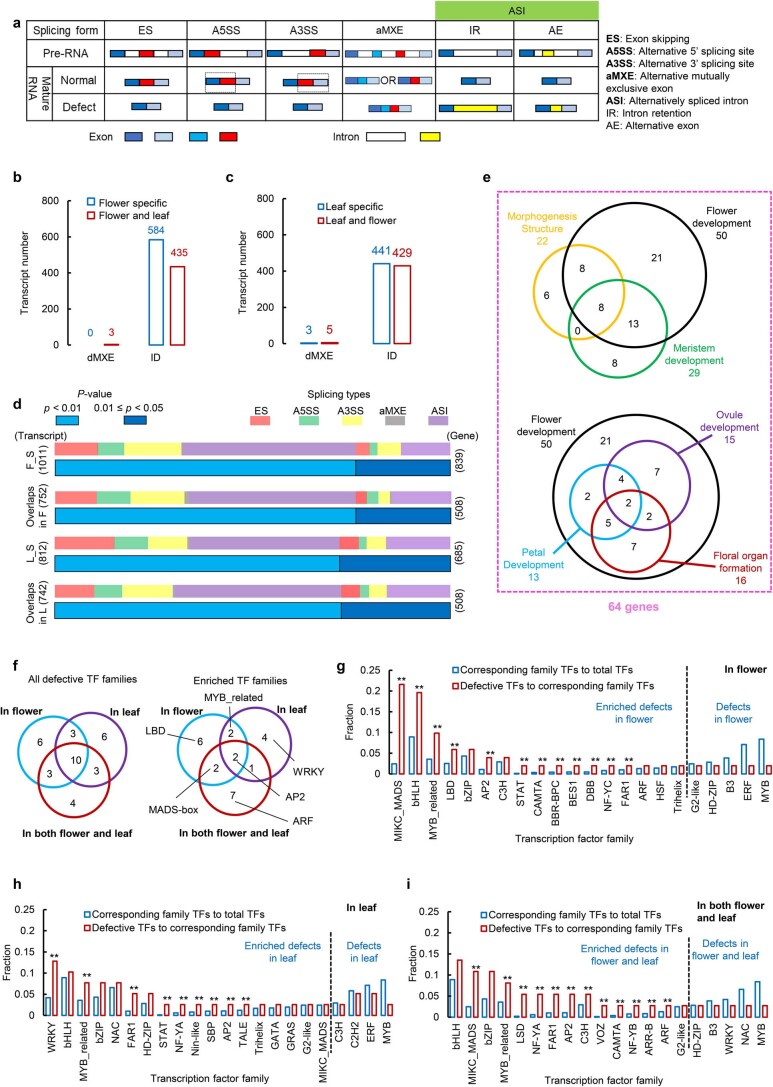
Analyses of pre-mRNA splicing in *grp20* flowers and leaves, as compared to WT. **a**, Illustrations of categories of splicing defects observed in this study. The categories are exon skipping (ES); alternative 3’ splicing site (A3SS); alternative 5’ splicing site (A5SS), alternative mutually exclusive exon (aMXE), and alternatively spliced intron (ASI), including intron retention (IR), delayed intron degradation (DID), and alternative exon (AE) inside an intron. The blue and red rectangles represent normal exons, yellow rectangles represent abnormal exonic sequences, and the white rectangles represent introns. **b**, Numbers of genes/transcripts with ASI and aMXE splicing defects in the flower-specific group and the overlapping group between flower and leaf. **c**, Numbers of genes/transcripts with ASI and aMXE splicing defects in the leaf-specific group and the overlapping group between leaf and flower. **d**, A heatmap of defective types and significance of each gene in flowers and leaves. *P*-values are categorized to two groups: *p* < 0.01 (blue) and 0.01 ≤ *p* < 0.05 (dark blue) (a likelihood-ratio test with 95% confidence intervals). The splicing defects are categorized into five groups including ES (light red), A3SS (yellow), A5SS (light green), aMXE (gray), and ASI (light purple). Among the splicing defects specifically detected in floral transcripts, ASI and ES are more frequent than other types. **e**, A Venn diagram of the overlap between genes in different GO categories. 64 genes in flower development (including petal development, ovule development and floral organ formation), morphogenesis structure and meristem development are highly enriched in defective transcripts in *grp20* flowers. **f**, A Venn diagram of the overlap between defective transcription factor families and enriched defective transcription factor families in flower specific (blue circle), in flower and leaf overlapping (red circle), and in leaf specific (purple circle) groups. **g-i**, Fractions of all annotated transcription factors containing abnormal transcripts to all transcription factors in the same family in *grp20* flower specific (**g**), leaf specific (**h**), and flower and leaf overlapping groups (**i**). The enrichment is compared to referenced fractions of transcription factors in each family to all 1,717 transcription factors. The enriched defects in flower and leaf are defined if the defective fraction is more than referenced fraction. Two asterisks (**) indicate enrichment of defects in corresponding transcription factor family if the defective fraction is more than twice of reference fraction.

**Extended Data Fig. 3:**
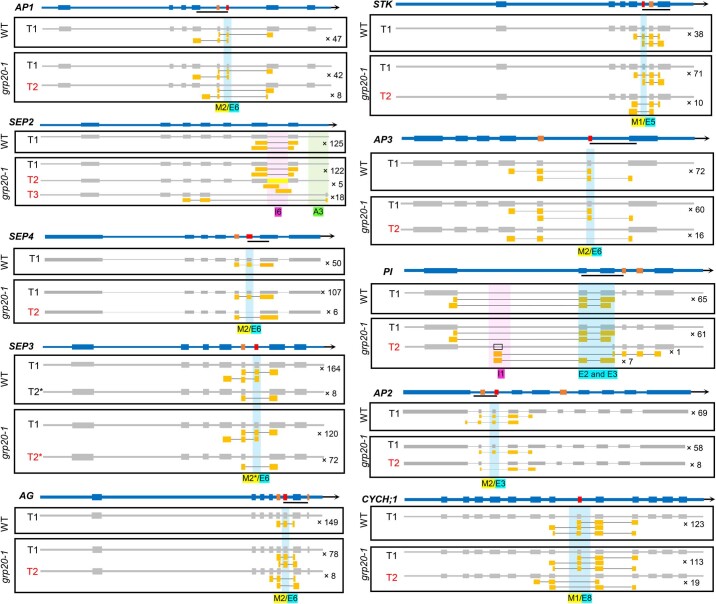
A summary of reads corresponding to wild-type and alternative transcripts in WT and *grp20* flowers for micro exon skipping and other defects. Illustrations of gene structures, transcripts and reads for floral homeotic MADS-box genes, *AP2* and *CYCH;1* including micro exons. The blue structure is gene structure, and grey structures are the transcripts in WT and *grp20* flowers. Most exons are shown as blue boxes and introns as blue lines between exons; skipped micro exons in *grp20* are highlighted as red boxes and other micro exons as brown boxes. The yellow bars represent reads (with thin lines for sequences that were absent) supporting the normal and abnormal transcripts. The × plus number near a transcript represents the read counts mapped to the affected region(s) supporting this transcript. The reads for MADS-box transcripts lacking the micro exons were observed in *grp20* flowers, but not in the WT. For example, there are 47 reads supporting the wild-type *AP1* transcript T1 in WT (× 47), but no WT reads supporting alternative transcripts; therefore, × 0 is omitted and the percentage of reads only observed in mutants is 0%. In *grp20* flowers, there are 42 reads supporting wild-type T1 (× 42); however, there are another 8 reads (× 8) supporting an alternative transcript T2, with the second micro exon skipped. Therefore, the percentage of alternative transcript is 16%. T1 and T2 in black represent wild-type transcripts that could be observed in WT. T2 and T3 in red represent alternative transcripts that only could be observed in *grp20* mutants. T2* in black in *SEP3* represents a transcript in WT with ~5% detection; in contrast, this transcript had substantially increased percentage in *grp20* flowers (~37%). ‘I’ indicates intron, ‘M’ indicates micro exon, ‘E’ represents exons, and ‘A3’ represents alternative 3’ site. The number indicates the order of the affected exon or intron. For example, M2 indicates the second micro exon in this gene. E6 indicates the sixth exon in this gene. I1 indicates the first intron in this gene. M2/E6 indicates the affected exon is both the second micro exon and sixth exon. The ES, ASI and A3SS defective regions are highlighted, respectively, by light blue, light pink and light green. The black lines under the gene structure indicate the regions amplified by primers used in qRT-PCR.

**Extended Data Fig. 4:**
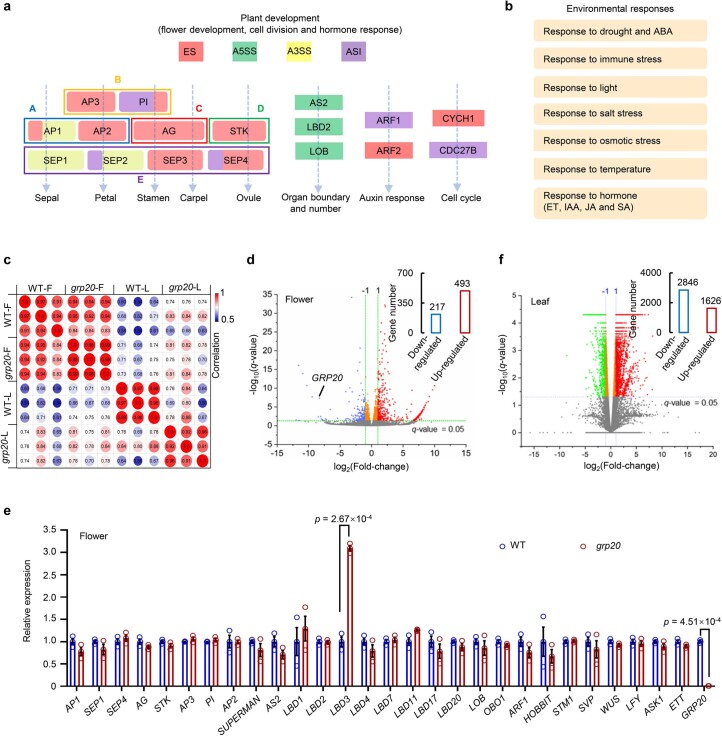
Transcriptomic analyses in WT and *grp20* flowers and leaves, and the comparison of DEGs in *grp20* and floral homeotic mutant flowers. **a**, The illustration of splicing defect types for floral homeotic genes, LOB domain genes, Auxin responsive factor (ARF) family genes, and cell division genes; the splicing defect types are indicated with red (ES), green (A5SS), yellow (A3SS), and purple [ASI (IR and AE)]. **b**, The illustration of wide defects of environmental response in *grp20*. **c**, Pearson correlation coefficient values of all three replicates in RNA-seq in WT and *grp20*. The red colors indicate high levels of correlation, with > 0.9 values for biological replicates of the same tissue. **d**, Differentially expressed genes (DEGs) in WT and *grp20-1* flowers. The expression of *GRP20* is indicated by a short line. The numbers of down-regulated and up-regulated genes in *grp20-1* flowers are shown at the top right. There are ~2% differential genes in *grp20* flowers, unlike the relatively large number of differential genes (1,000-2,000) affected by floral homeotic mutants^80^. **e**, The relative expression of flower developmental genes in WT and *grp20-1* flowers by RT-qPCR. Genes include floral homeotic MADS-box genes (*AP1* to *PI*), *AP2*, LOB domain genes (*AS2* to *LOB*), and other flower developmental genes (*SUPERMAN*, *OBO1*, *ARF1* to *ETT*). The expression levels were normalized to WT being ‘1’. The expression of *GRP20* is greatly reduced in the mutant as expected. The expression levels of known floral regulatory genes were not significantly different between the wild-type and *grp20* flowers, except for *LOB DOMAIN 3* (*LBD3*). The experiments are conducted with three independent replicates and two-sided Student’s *t* test is used for statistics. Data are presented as mean ± SEM. **f**, Differentially expressed genes (DEGs) in WT and *grp20-1* leaves. The expression of *GRP20* is indicated by a short line. For **d** and **f**, the X-axis is log_2_(fold-change of [*grp20-1* to WT]) and Y-axis is log_10_(*q*-value). The cut-off for DEGs is log_2_(fold-change) ≥ 1 or log_2_(fold-change) ≤ -1 (vertical green dash lines) and *q-value* < 0.05 (horizontal green dash line). The numbers of down-regulated and up-regulated genes in *grp20-1* leaves are shown at the top right. Source data

Our analyses indicate that there were five types of alternative transcripts (Extended Data Fig. 2a) in flowers (Fig. 2a, Extended Data Fig. 2b,d and Supplementary Table 1) and leaves (Fig. 2b, Extended Data Fig. 2c,d and Supplementary Table 1). In particular, increased floral transcripts in *grp20* with exon skipping were detected for several MADS-box genes (*AP1*, *AP3*, *AG*, *STK* and *SEP4*) and *AP2* (Figs. 1d and 2c, and Extended Data Figs. 2e–g and 3), suggesting the importance of GRP20 in the splicing of flower transcripts. Also, splicing changes were observed for genes regulating cell division, such as *CYCH;1* (exon skipping (ES)) and *HOBBIT* (alternatively spliced intron (ASI); Fig. 1d and Extended Data Fig. 3), and for epigenetic regulation, such as *LHP1* (alternative 3′ splicing sites (A3SS)) and *SUVH9* (ES) in flowers (Figs. 1d and 2c, and Supplementary Table 1). Moreover, genes related to hormonal signalling and stress responses, including auxin responsive factors and heat shock proteins, were enriched among genes with increased alternative transcripts in both *grp20* flowers and leaves (Figs. 1d and 2c), whereas transcripts of housekeeping genes, such as meristem stem cell regulator *WUS*, were similar to the wild type in flowers, suggesting that GRP20 regulates proper splicing of a specific subset of florally expressed pre-mRNAs. Notably, alternatively spliced transcripts with ES, ASI, A3SS and alternative 5′ splicing site (A5SS) were observed in leaves for genes that are responsive to diverse stresses, such as AP2 family genes for abiotic stresses, *NPR4* for disease response and others (Fig. 2d and Supplementary Table 1), suggesting that GRP20 has a broad impact on splicing of genes involved in environmental responses. Moreover, notably increased numbers of reads for alternatively spliced transcript were detected in *grp20* leaves compared with the WT for epigenetic regulators and those of flowering time, such as *HDT4* and MADS-box genes (Fig. 2d and Supplementary Table 1). In addition, alternative transcripts were detected in *grp20* leaves, but not in the WT, for genes in circadian rhythm and protein ubiquitination (Fig. 2d and Supplementary Table 1). The differences in splicing of transcripts of crucial genes in *grp20* indicate that GRP20 is a novel regulator of RNA splicing for genes important for development and predicted for environmental responses. Transcriptional regulatory genes are generally affected in both *grp20* flowers and leaves. Among 1,717 annotated transcription factors in 58 gene families, distinct families are enriched among those with alternative transcripts in the flower and/or leaf (Extended Data Fig. 2f). In addition to MIKC-type MADS-box genes, LOB (lateral organ boundaries) domain genes related to reproduction were highly enriched in the *grp20* flower (Extended Data Fig. 2f,g). However, stress-responsive and leaf developmental factor genes including WRKY (WRKYGQK heptapeptide) and NAC (an acronym of NAM, ATAF1-2 and CUC2) family members were enriched among genes with alternative transcripts in the *grp20* leaf (Extended Data Fig. 2f,h). Moreover, MYB-related, bHLH, bZIP and AP2 family members were observed with alternative transcripts in both flowers and leaves (Extended Data Fig. 2f,i), supporting the idea that GRP20 affects the splicing of transcripts for multiple regulators of transcription (Extended Data Fig. 4a,b). We then estimated the levels of the typical transcripts for MADS-box genes, *AP2* and LOB domain gene *AS2* using RT-qPCR (RT-quantitative PCR) and found that they were reduced significantly in the *grp20* flower to about 40–60% of the WT levels (Figs. 2e and 3a). In addition, the levels of typical transcripts in the *grp20* leaf for *ING2*, *WRKY* and *RHC1A* genes were also reduced to about 50–80% of the WT levels (Fig. 2f), supporting the changes in splicing observed in transcriptomic analyses.

We also investigated the differential gene expression level between WT and *grp20* stage 1–12 flowers and leaves with three biological replicates (Extended Data Fig. 4c and Supplementary Table 2), allowing the identification of 217 downregulated genes including *GRP20* and 493 upregulated genes (Extended Data Fig. 4d) (~2% of total genes, 710 of 35,000) in flowers. Specifically, the expression levels of known homeotic genes and other MADS-box genes, LOB domain genes and other flower developmental genes were not significantly different between the wild-type and *grp20* flowers (Extended Data Fig. 4e). Thus, the *grp20* mutation does not affect the mRNA levels of most floral regulatory genes, but specifically affects RNA splicing. However, about 2,800 downregulated genes including *GRP20* and 1,600 upregulated genes were identified in *grp20* leaves (Extended Data Fig. 4f and Supplementary Table 2). It is possible that splicing defects for various environmental stress-responsive genes in leaves led to feedback on gene expression and greater numbers of the differentially expressed genes in the leaves than in the flowers.

### GRP20 regulates micro-exon and small-exon splicing

**Extended Data Fig. 5:**
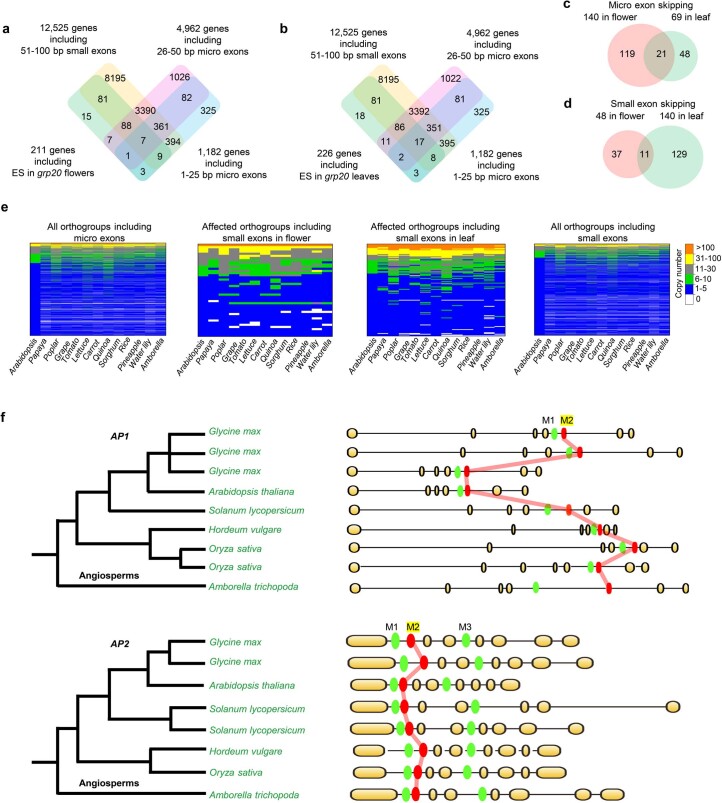
The micro exons and small exons in alternative transcripts in *grp20* flowers and leaves. **a, b**, Venn diagrams of the overlap among 211 genes with exon skipping in *grp20* flowers (**a**) or 226 genes with exon skipping in *grp20* leaves (**b**), and numbers of *Arabidopsis* genes containing small exons (51-100 nucleotides) and micro exons with lengths (inclusive) of 26-50 nucleotides, or ≤ 25 nucleotides, respectively. Totally 114 genes in *grp20* flowers and 69 genes in *grp20* leaves with exon skipping overlap with the above two groups of genes containing micro exons ≤ 50 nucleotides. A total of 48 genes in *grp20* flowers and 140 genes in *grp20* leaves with exon skipping overlap with the group containing small exons. The data suggested the idea that small exons and micro exons are important gene-structural elements in plant genomes. **c, d**, Venn diagrams of overlaps for skipped micro exon (**c**) and skipped small exon (**d**) in flowers and/or leaves. **e**, Heatmaps of copy number with all referenced orthogroups and defective orthogroups including micro exons and small exons in *grp20* flowers and leaves among thirteen angiosperms (*Arabidopsis* to *Amborella*). Protein ubiquitination and degradation genes (*UBP15* and *PBC1*), drought responsive proteins (*DI19*), epigenetic factor (*BONSAI* (*BNS*)) and meiotic gene (*RAD50*) are found in the small exon skipping in flowers. In addition, responsive factors to GA (vacuolar sorting protein), JA (*NINJA*), high light (*DEG5*) and cold (*PFC1*), regulators for leaf morphology (*BLH4*, *ANU7*, *ACA4* and others), protein modification genes (*MEKK1* and other kinases, and *PUB9* and other U-box proteins), transcription factors (*AGL31*, *AGL42*, *NF-YA7* and others), cell death inducer (*ACD6*), epigenetic regulators (methyltransferases) and polypyrimidine tract binding protein (*PTB1*) were also observed in affected small exons in leaves. **f**, Gene structures of *AP1* and *AP2* homologues among representative angiosperms. On the left are the phylogenetic relationships of selected angiosperm *AP1* and *AP2* homologs, respectively, and on the right part are the corresponding gene structures. The elliptical boxes indicate exons, and the thin lines represent introns. Red and green highlight micro exons (M1, M2 and M3), with M2 (red) being skipped in *grp20* mutants. Gene structure comparison indicates the skipped micro exons are conserved in angiosperms and are linked by light red lines. Intron sizes are not to scale.

To assess the parameters of GRP20-targeted transcripts for ES, we examined the lengths of affected exons in *grp20* flowers and leaves. The results indicated that the average length of affected exons was 50 nucleotides in both flowers and leaves, much shorter than that of all exons in flowers and leaves (Fig. 3b,c) and below the average exon length of 180 nucleotides in plants. The *Arabidopsis* genome contains 150,204 exons in 23,910 genes with more than one exon and 12,867 genes without an intron. Among 150,204 exons, as mentioned before, >8,000 are micro exons found in ~6,000 genes; in addition, 39,025 small exons (51–100 nucleotides) are present in 12,525 genes (Fig. 3d,e and Extended Data Fig. 5a,b). Among the *Arabidopsis* micro exons, 185 (in 183 genes) have fewer than 10 nucleotides, 1,132 (999 genes) have 10–25 nucleotides and 6,745 (4,962 genes) have 26 to 50 nucleotides (Fig. 3d,e, Extended Data Fig. 5a,b and Supplementary Table 3). As a further test for mapping efficiency of the transcriptome datasets, we examined the results for additional micro exons with ≤25 nt. Among 1,186 genes with 1,317 annotated micro exons of ≤25 nt, 863 genes showed expression in flowers with detected reads. The reads for 851 genes were mapped to gene regions including micro exons, resulting in the detection of 922 micro exons, whereas reads for 12 genes were mapped to regions lacking micro exons. In the *grp20* mutant flowers, reads for 839 genes were mapped to regions including micro exons (reads for 24 genes were in regions lacking micro exons), with detection of 912 micro exons, although no additional micro exons had notable difference in splicing between WT and *grp20*. These results indicate that nearly all micro exons with ≤25 nt of florally expressed genes were detected in both WT and *grp20* transcriptome datasets.

We found that 238 exons were skipped in 211 *grp20* floral transcripts (Extended Data Fig. 5), including 59% (140 of 238) micro exons (120 with 26–50 nucleotides) and 20% (48 of 238) small exons (51–100 nucleotides; Fig. 3d,f and Supplementary Table 3). In addition, 265 exons were skipped in 226 leaf transcripts (Extended Data Fig. 5), with 26% (69 of 265) and 53% (140 of 265) being micro exons and small exons, respectively (Fig. 3e,f and Supplementary Table 3). Among exons of different sizes in the genome, the skipped micro exons in flowers and both the skipped micro and small exons in leaves were enriched (Fig. 3g). Only small numbers of micro exons and small exons were skipped in both floral and leaf transcripts (Extended Data Fig. 5c,d). These results indicate that GRP20 preferentially promotes proper retention of micro and small exons with largely distinct sets of targets in flowers and leaves. To obtain additional clues regarding the functions of affected transcripts with missing micro or small exons, we identified several enriched GO categories (Fig. 3h and Supplementary Table 3) including floral organ identity, meiosis, transcriptional regulation, RNA modification and metabolism for floral transcripts, and transcriptional regulation, stomatal opening, autophagy, cell differentiation, cell death and environmental responses for leaf transcripts (Fig. 3h and Supplementary Table 3). The results suggested that GRP20 is a major regulator for micro-exon and small-exon splicing for genes involved in or annotated for plant growth and environmental responses. Moreover, other exons affected by GRP20 are in genes also implicated in normal development and predicted for response to environment (Supplementary Table 3).

As *GRP20* is expressed at lower levels in the leaf than in the flower, it is possible that leaf transcripts might show micro-exon skipping more frequently. To test this idea, we compared 20,810 genes expressed in both the WT flower and leaf and detected reads supporting skipping of 23 micro exons in the leaf and skipping of 21 other micro exons in the flower. It is possible that the regulation of micro-exon retention in the leaf might involve other unknown factors.

Micro exons with 10 to 50 nucleotides (Fig. 3d) are enriched in floral homeotic genes in flowers, and some of them had alternative transcripts with skipped micro exons in *grp20* flowers (Fig. 3h), including a micro exon encoding a part of the K domain in several floral homeotic MADS-box genes (*AP1*, *SEP4*, *SEP3*, *AG*, *STK* and *AP3*). In addition, a micro exon encoding a part of the AP2 domain in *AP2* and *TOE2* was skipped in some *grp20* floral transcripts (Extended Data Fig. 3). In particular, 5–22% reads for MADS-box and *AP2* transcripts lacking the micro exons were detected in *grp20*, but not in the WT (Extended Data Fig. 3). Also, transcripts lacking micro exons were observed for genes regulating cell division, such as *CYCH;1* (Extended Data Fig. 3). The levels of alternative transcripts showing micro-exon skipping in *AP3* and *AP2*, and A5SS in *LBD2*, were found to significantly increase in *grp20* relative to that in WT (Fig. 3i,j). The increased production of such alternative transcripts in *grp20* flowers missing an exon and containing other splicing differences might have caused a reduction of the annotated WT protein level and activity.

### Identification of putative GRP20 homologues among plants

**Fig. 4:**
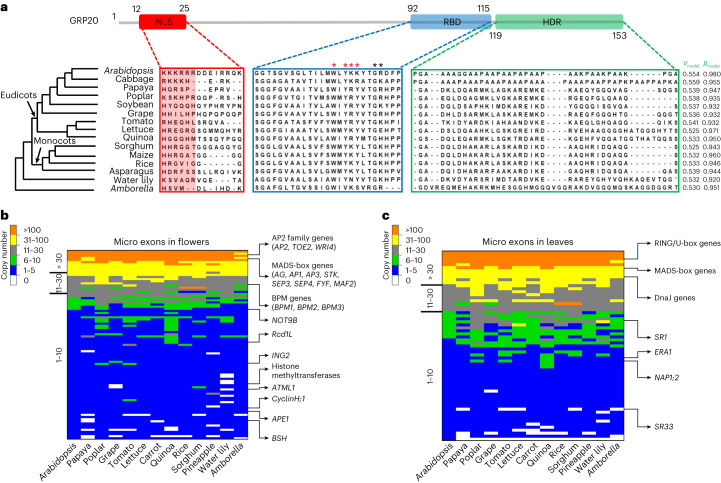
Identification of putative GRP20 homologues among plants. **a**, The alignment of NLS (residue 12 to 25, red box), the putative RBD (residue 90 to 114, blue box) and highly disorder region (residue 119 to 153, green box) in GRP20 homologues from 15 angiosperm species, including 9 eudicots (in the top clade of the tree), 4 monocots (in the second clade), water lily (*Nymphaea colorata*) and *Amborella* (*A. trichopoda*, sister to other angiosperms). The positively charged amino acids, the R, K and H region (residue 12 to 17), in NLS are highlighted in light red. The residues in GRP20 RBD predicted to bind RNA are indicated with asterisks above the GRP20 amino acid sequence: 103W, 105Y, 106K, 107K, 110G and 111R. Specifically, 103W, 105Y, 106K and 107K (red asterisks) were altered in a mutant construct to test for RNA binding, as shown in Fig. 7 and Extended Data Fig. 8. The disorder confidence scores along HDRs and whether HDRs can undergo condensation formation of putative GRP20 homologues are shown by *V*_model_ and *β*_model_ (*β*-turn propensity). The ParSe program was used in this prediction. *V*_model_ < 0.56 and *β*_model_ > 0.9 indicate that the domain is intrinsically disordered and prone to undergo condensation formation or fold to a stable conformation. **b**,**c**, The heat maps of copy number with affected micro exons in *grp20* flowers (**b**) and leaves (**c**) among 13 angiosperms (*Arabidopsis* to *Amborella*). The orthogroups including MADS-box genes and AP2 family genes (including nearly all floral homeotic genes), cell cyclin genes (*NOT9B*, *CYCH;1* and *Rcd1L*), transcription factors (homeodomain-like gene *GT-1* and homeobox gene *ATML1*), epigenetic regulators (H3K4 methylation reader *ING2*, chromatin remodeler *BSH* and histone methyltransferase), proteosome-degradation genes (MATH-BTB domain genes *BPM1*, *BPM2* and *BPM3*), acclimation of photosynthesis to environment gene *APE1* and other essential genes are indicated in the right panel of the flower heat map. Orthogroups including transcription factors (MADS-box gene *AGL42*), epigenetic regulators (*NAP1;2*), protein ubiquitination genes (RING/U-box genes), ABA responsive genes (*ERA1*), protein chaperons (DnaJ genes), splicing factors (*SR33* and *SR1*) and protein kinases are indicated in the right panel of the leaf heat map.

As a first step to learn whether GRP20 function in RNA splicing might be conserved among plants, we retrieved the sequences of putative GRP20 homologues from 14 representative angiosperms. Earlier in this study, we described three GRP20 functional domains, NLS, RBD and HDR (Fig. 1b). A comparison of GRP20 with its putative homologues indicates that the GRP20 RBD exhibits sequence similarity to corresponding regions in the GRP20 homologues (Fig. 4a). Moreover, the putative GRP20 homologues also have predicted NLS with positively charged amino acid residues (Fig. 4a), suggesting that they might also be nuclear proteins. Although the GRP20 HDR, by its disordered nature, does not require a specific sequence for function, we analysed the corresponding regions of putative GRP20 homologues for their disordered propensity by using a computational program with *V*_model_ and *β*_model_, which supported the C-terminal region of each of the 14 putative GRP20 homologues with disorder characteristics (*V*_model_ < 0.56 and *β*_model_ > 0.9; Fig. 4a).

A crucial function of GRP20 is to promote micro- and small-exon retention; thus, we examined the orthogroups including genes with affected micro exons and small exons. Among the orthogroups with at least one gene that has a micro exon affected in *grp20*, a majority have only 1–5 genes (61 of 90 flowers and 33 of 63 leaves; Fig. 4b,c, Extended Data Fig. 5e and Supplementary Tables 3 and 4); this pattern is also found for orthogroups containing genes whose small exons are affected (Extended Data Fig. 5e and Supplementary Tables 3 and 4). Specifically, affected micro exons were present in MADS-box gene (ABCE genes and others) and AP2 gene family members (*AP2*, *TOE2* and *WRI4*) and are conserved (Fig. 4b and Extended Data Fig. 5f), suggesting that these transcripts also needed to be properly spliced for normal function in other plants. In addition, other conserved genes affected by micro-exon skipping include cell division and differentiation genes (*CYCH;1*, *NOT9B* and *Rcd1L*), epigenetic factor, transcription factors and others (Fig. 4b and Supplementary Tables 3 and 4). Moreover, drought-responsive proteins, epigenetic factor and meiotic gene are among the genes with small-exon skipping in flowers (Supplementary Table 3). On the other hand, genes with leaf transcripts affected by micro-exon skipping are those for transcriptional regulation, hormone response and splicing (Fig. 4c and Supplementary Tables 3 and 4); for genes affected by small-exon skipping in the leaf, the implicated functions include response to hormone and stresses, leaf morphology, induction of cell death and poly-pyrimidine tract binding (Supplementary Tables 3 and 4).

### GRP20 is required for normal floral organ development

**Fig. 5:**
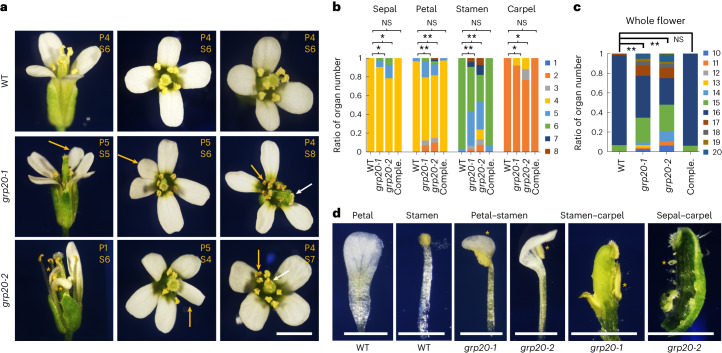
Floral phenotypes in the wild type and *grp20* mutants. **a**, The floral phenotypes in the WT and *grp20* mutants. The yellow letters and numbers at the top right of each panel indicate the corresponding organ and number in the flower. P, petal; S, stamen. In WT flowers, there were four petals and six stamens; in *grp20-1* flowers, four to five petals and five to eight stamens; and in *grp20-2* flowers, one to five petals and four to seven stamens. Yellow arrows and asterisks indicate extra organs (petal or stamen, arrows) and chimeric organs (fusion of petal and stamen tissues, asterisks), respectively. White arrows indicate abnormal carpels. Scale bar = 1 mm. **b**, Number of floral organs in flowers of different genotypes (flower counts: WT, 80; *grp20-1*, 85; *grp20-2*, 90; comple., 95). In *grp20* mutants, there were three to five sepals, two to six petals, two to eight stamens and two to four carpels whereas the wild type had four sepals, four petals, five to six stamens and two carpels. Data are presented as the fraction (ratio) of the floral organ number to the total organ count in **b** and **c**, and detailed flower counts are shown in Supplementary Table 5. For examples, all 80 flowers of the WT have 4 sepals; therefore, the fraction of 4 in the WT is 1 (80/80). However, among 85 flowers of *grp20-1*, 74, 9 and 2 flowers have 4, 5 and 6 sepals, respectively. Therefore, the fractions of 4, 5 and 6 sepals in *grp20-1* are 0.870 (74/85), 0.106 (9/85) and 0.024 (2/85), respectively. The *P* values are shown in the following order (from left to right) in **b** and **c**: WT versus *grp20-1*, WT versus *grp20-2* and WT versus complementation. For ‘Sepal’, *P* = 0.0167 and 0.01; for ‘Petal’, *P* = 0.0063 and 0.0082; for ‘Stamen’, *P* = 0.0014 and 0.0045; for ‘Carpel’, *P* = 0.013 and 0.018. **c**, The total organ number in a WT flower, *grp20* mutants and the complementation line (flower counts: WT, 60; *grp20-1*, 75; *grp20-2*, 48; comple., 66); 35% and 28% of the flowers in *grp20-1* and *grp20-2*, respectively, showed abnormal organ numbers. From left to right, *P* = 0.007 and 0.0025. In **b** and **c**, ‘Comple.’ is the complementation line with *Pro*_*GRP20*_*::GRP20-YFP* in the *grp20-1* background. Data are presented as mean ± s.d. for indicated flower counts; *P < 0.05; **P < 0.01, two-sided Student’s *t*-test. **d**, The floral organ identity phenotypes of *grp20* mutants. The normal petal and stamen in the WT are shown in the first two panels. Chimeric organs between the petal–stamen, stamen–carpel and sepal–carpel in the *grp20* mutants are shown in the other four panels. Scale bars = 500 μm. The yellow asterisks point to specific fused organs.

**Extended Data Fig. 6:**
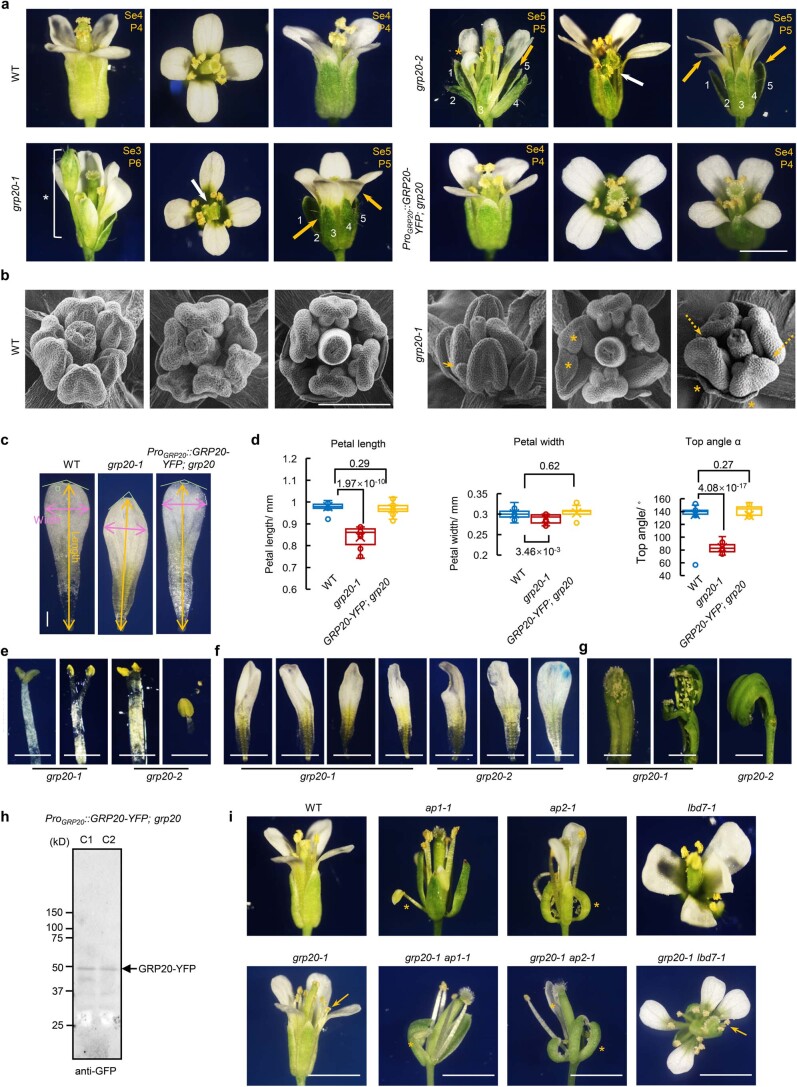
Floral phenotypes in WT, *grp20*, and the complementation plants (*ProGRP20::GRP20-YFP; grp20*), and flower phenotypes of double mutants between *GRP20* and floral homeotic genes or LOB domain gene. **a**, Sepal and carpel defects in *grp20* mutants and complementation lines. WT flowers show normal organ number and morphology. Flower defects are shown in *grp20-1* including in the left panel, the white asterisk indicates a floral bud at the position of a sepal in *grp20-1*; a white arrow in the middle panel indicates an unfused carpel in *grp20-1* and yellow arrows in the right panel indicate an extra sepal and petal, respectively. Flower defects were shown in *grp20-2* including an extra sepal (indicated by an orange arrow; total 5) and a curved carpel (indicated by a white arrow) are shown in the left and middle panels, respectively; an extra sepal and extra petal (total 5 sepals and 5 petals) in the right panel are indicated by orange arrows. Normal flowers are shown in *Pro*_*GRP20*_*::GRP20-YFP;grp20* complementation line. Bar = 1 mm. The yellow letter and number at top right of each panel indicate corresponding organ and number in the flower. Se: Sepal; P: Petal. The white number indicates sepal count in a flower. **b**, Scanning electron microscope (SEM) images of floral phenotypes in WT (left) and *grp20-1* (right). An extra stamen is indicated by an orange arrow in the left panel in *grp20-1*. Fused anthers and petals are indicated by the orange asterisks in the middle panel and the right panel, respectively in *grp20-1*. The positions of missing stamens are indicated by dashed orange arrows in the right panel in *grp20-1*. Bar = 20 μm. **c**, Petal length, width, and top angle in WT, *grp20* and complementation lines. Petal length (pl), width (pw) and top angle (α) are indicated by the orange double arrowed line, pink double arrowed line, and light green angle α, respectively. Bar = 100 μm. **d**, Reduced pl in *grp20-1*: (difference of pl in WT and *grp20-1*)/(average pl in WT) = (0.98-0.84) mm /0.98 mm = 14%. Flower counts for petal length: WT, 15; *grp20-1*, 18; and *Pro*_*GRP20*_*::GRP20-YFP grp20*, 18. Slightly reduced petal width in WT, *grp20* and complementation plants. Flower counts for petal width: WT: 16, *grp20-1*: 20 and *Pro*_*GRP20*_*::GRP20-YFP; grp20*: 18. Reduced top angle α in *grp20-1*. (Reduced α in *grp20-1*)/(average α in WT)= (140-83)/ 140 = 40%. Flower counts: WT: 16; *grp20-1*: 15; *Pro*_*GRP20*_*::GRP20-YFP;grp20*: 15. two-sided Student’s *t* test is used for statistics; The lower bound, maxima, minima, centre and upper bound of box plots (from left to right) is shown as: For pl: WT: 1.36, 1.41, 1.29, 1.375, 1.39; *grp20-1*: 1.105,1.24, 1.04, 1.2, 1.225; *GRP20-YFP (grp20)*: 1.33, 1.43, 1.28, 1.36, 1.39. For pw: WT: 0.41, 0.46, 0.39, 0.42, 0.43; *grp20-1*: 0.39, 0.42, 0.38, 0.41, 0.42; *GRP20-YFP (grp20)*: 0.42, 0.46, 0.39, 0.43, 0.435. For top angle α: WT: 136, 152, 132, 83.6, 92; *grp20-1*: 75, 101, 72, 83.6, 92; *GRP20-YFP (grp20)*: 134.25, 154, 132, 146, 148.75. **e**, Stamen phenotypes in *grp20* mutants. **f**, Petal morphologies in *grp20* mutants. **g**, Carpel phenotypes in *grp20* mutants. Bar = 300 μm. **h**, Western blot analysis of GRP20-YFP in two complementation lines (C1 and C2) with the *Pro*_*GRP20*_*::GRP20-YFP* transgene in *grp20*. A GFP antibody was used against whole cell lysate from transgenic flowers. **i**, Mature flowers of double mutants between *grp20* and homeotic mutants *ap1-1*, *ap2-1* and LOB domain mutant *lbd7-1*. The yellow arrows indicate extra stamen in *grp20-1* and *grp20-1 lbd7-1*. The asterisks indicate chimeric organs in *ap1-1*, *grp20-1 ap1-1*, *ap2-1* and *grp20-1 ap2-1*. The double mutants *grp20-1 ap1-1*, *grp20-1 ap2-1* and *grp20-1 lbd7-1* show more severe floral organ defects including various organ numbers compared to single mutants. Bar = 1 mm. Source data

The effects of GRP20 on floral RNA splicing, especially micro-exon retention of crucial floral homeotic MADS-box and *AP2* genes (Figs. 3a,j and 4b, and Extended Data Fig. 3), suggest that *GRP20* might be involved in flower development. Thus, we investigated flower development of the *grp20* mutants (Extended Data Fig. 1h). Compared with wild-type flowers (Fig. 5a and Extended Data Fig. 6a), about 30% of *grp20* flowers showed organ defects, including altered numbers of sepals, petals, stamens and carpels (Fig. 5a,b, Extended Data Fig. 6a,b and Supplementary Table 5); the total number of *grp20* floral organs also varied from 12 to 20 (Fig. 5c and Supplementary Table 5). Other *grp20* floral organ abnormalities included reduced petal length and angle between the distal margins, stamens with fused filaments or anthers, abnormally large anther with a shorter filament and others (Extended Data Fig. 6c,d). Moreover, organ identity defects were found in *grp20* flowers, including chimeric (fused) organs with petal-like and stamen-like portions, stamen–carpel portions and sepal–carpel parts (Fig. 5d and Extended Data Fig. 6e–g); finally, floral meristem defect was also infrequently seen with a complete floral bud occupying the position of the sepal (Extended Data Fig. 6a, bottom left panel), resembling an *ap1* mutant flower. To verify that the defects were caused by the *grp20* mutation, we introduced a fusion of the *GRP20* promoter with its coding region into the *grp20* mutant background (Extended Data Fig. 6h) and found that flowers of transgenic plants were normal (Fig. 5b,c and Extended Data Fig. 6a,c,d). Therefore, *GRP20* is required for normal flower development and affects organ patterning.

**Fig. 6:**
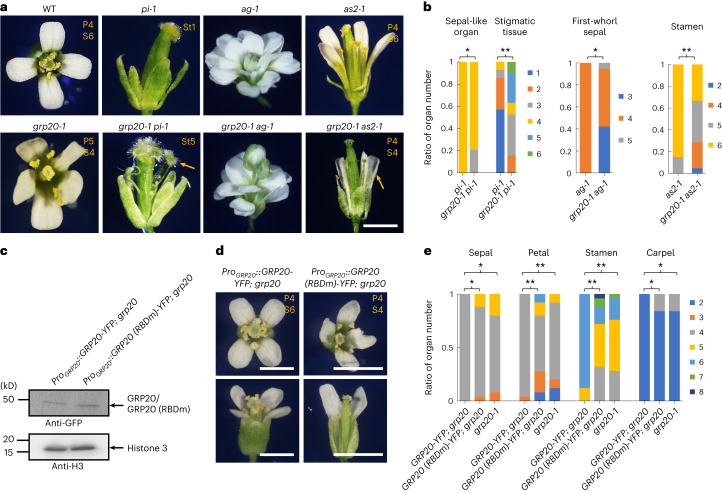
RBD is required for flower development. **a**, The flower phenotypes of *grp20-1 pi-1*, *grp20-1 ag-1* and *grp20-1 as2-1* double mutants. Scale bar = 1 mm. The yellow arrows indicate the stronger phenotypes in double mutants compared with single mutants: more stigmatic tissues in *grp20-1 pi-1* and shorter petals in *grp20-1 as2-1*. The asterisk indicates an abnormal chimeric organ. The yellow letters and numbers at the top right of each panel indicate corresponding organs and numbers in the flower. P, petal; S, stamen; St, stigmatic tissue. **b**, Number of floral organs in flowers of single and double mutants. The number of sepal-like organs in the second whorl and stigmatic tissues in *pi-1* and *grp20-1 pi-1* (flower counts: *pi-1*, 19 for sepal-like organs and 14 for stigmatic tissues; *grp20-1 pi-1*: 19 for sepal-like organs and 19 for stigmatic tissues). The number of first-whorl sepals in *ag-1* and *grp20-1 ag-1* (flower counts: *ag-1*, 19; *grp20-1 ag-1*, 19). The number of stamens in *as2-1* and *grp20-1 as2-1* (flower counts: *as2-1*, 20; *grp20-1 as2-1*, 21). From left to right, *P* = 0.041, 5.24 × 10^−6^, 0.015 and 8.74 × 10^−4^. **c**–**e**, Analyses of transgenic plants expressing GRP20 proteins with normal or mutant RBD (RBDm), as shown in Extended Data Fig. 9a. **c**, Relative protein expression levels in transgenic plants. Anti-histone 3 was used as a loading control. **d**, Mature flowers in transgenic plants. Yellow letters at the top right of each panel indicate the organ and corresponding number. Scale bars = 1 mm. **e**, Floral organ numbers in transgenic plants. Flower counts: *Pro*_*GRP20*_*::GRP20-YFP grp20*, 25; *Pro*_*GRP20*_*::GRP20 (RBDm)-YFP grp20*, 25; and *grp20-1*, 25. From left to right, for ‘Sepal’, *P* = 0.042 and 0.043; for ‘Petal’, *P* = 0.0024 and 2.08 × 10^−5^; for ‘Stamen’, *P* = 0.0085 and 0.0022; for ‘Carpel’, *P* = 0.033 and 0.027. For **b** and **e**, data are presented as mean ± s.d. for indicated flower counts; *P < 0.05; **P < 0.01, two-sided Student’s *t*-test. Source data

To further test whether the *grp20* floral defects were related to the reduced function of floral regulatory genes exhibiting splicing defects, we generated relevant double mutants and examined their floral phenotypes. For example, the *grp20-1 pi-1* double mutant showed a relatively reduced number of sepal-like organs in the second whorl (Fig. 6a,b and Supplementary Table 5), consistent with the idea that a part of *grp20* defects was due to reduced *PI* WT transcript. In addition, the *grp20-1 pi-1* flower produced unfused carpels with more than four stigmata, more severe than *pi-1* single mutants (Fig. 6a,b and Supplementary Table 5), in agreement with the observation that genes other than *PI* also were alternatively spliced in *grp20* flowers. Similarly, the *grp20-1 ag-1* double mutant also showed floral defects different from those of *ag-1*, including a decreased number of first-whorl sepals (Fig. 6a,b and Supplementary Table 5). Double mutants of *grp20-1* and *as2-1* reduced the number of stamens and flower (organ) size compared with single mutant *as2-1* (Fig. 6a,b and Supplementary Table 5). In addition, double mutants of *grp20-1* with mutations in other floral homeotic and LOB domain genes including *ap1*, *ap2* and *lbd7* also showed more severe defects than the corresponding single mutants in some aspects of floral organ identity and morphology (Extended Data Fig. 6i), supporting the idea that GRP20 regulates flower development, at least in part by affecting the splicing of some homeotic genes and organ boundary genes (Extended Data Fig. 4a).

To further test whether the *grp20* floral defects were related to the skipping of micro exons in transcripts of floral regulatory genes, we generated transgenic plants that contain fusions of the native promoter to the normal floral gene coding complementary DNA (cDNA; containing micro exons) for one of the A, B and E functions, including (1) A function, *AP1* and *AP2*; (2) B function, *AP3* and *PI*; and (3) E function, *SEP3* and *SEP4*, in the *grp20-1* background (Supplementary Fig. 2a–c). These transgenic plants produced flowers with partially restored phenotypes consistent with the increased levels of one of the ABE functions, including floral organ number and morphology compared with those of *grp20* (Supplementary Fig. 2e,f), suggesting that they are able to partially rescue the defects in *grp20* mutants. As controls, transgenic plants were also generated expressing the corresponding transgenes (*AP1* and *AP2*, *AP3* and *PI*, *SEP3* and *SEP4*) lacking the micro exons. Although these transgenes were expressed at similar levels (Supplementary Fig. 2c,d), the transgenic plants showed flower defects similar to those of the *grp20* mutants (Supplementary Fig. 2e,f). These results indicate that the function of GRP20 in flower development is at least in part dependent on micro-exon (and small exon) splicing of floral regulatory genes.

Similarly, we introduced transgenes for either the full-length *AS2* coding sequence (CDS) or a 5′ alternatively spliced *AS2* transcript into the *grp20-1* background (Supplementary Fig. 2a,b,d). We found that the defects of size and morphology are partially rescued in transgenic plants with full-length *AS2* CDS (Supplementary Fig. 2e), but the transgenic plants with the 5′ alternatively spliced *AS2* transcript showed similar flower size and morphology to those of *grp20-1* (Supplementary Fig. 2e). The results further support the idea that proper splicing regulated by GRP20 is important for normal flower development.

**Extended Data Fig. 7:**
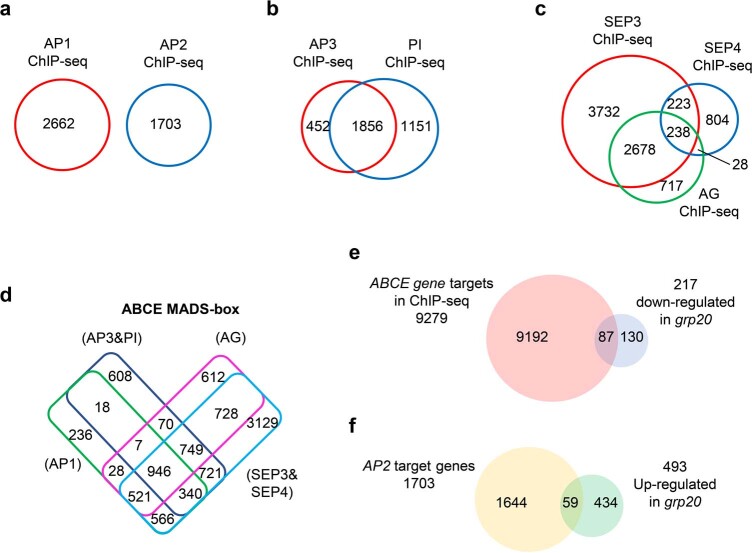
The comparison of DEGs in *grp20* and floral homeotic mutants. **a-c**, Venn diagrams of potential target genes of A function AP1 or AP2 proteins (**a**), B function AP3 or PI proteins (**b**) and C (AG) and E function (SEP3 and SEP4) proteins (**c**). **d**, A Venn diagram of the overlap among potential targets of ABCE (AP1, SEP4, SEP3, AG, AP3 and PI) proteins. Total 9279 genes might be putative targets of ABCE MADS-box genes. **e**, A Venn diagram of the overlap between the potential ABCE (AP1, SEP4, SEP3, AG, AP3, and PI) target genes and down-regulated genes in *grp20* flowers. 40% (87/217) of down-regulated genes in *grp20* flowers among putative MADS-box protein targets. **f**, A Venn diagram of the overlap between potential AP2 target genes and up-regulated genes in *grp20*. 12% (59/493) of up-regulated genes in *grp20* flowers among putative AP2 targets. AP1, AP3, PI, AG, SEP3, and SEP4 are known positive regulators of gene expression; however, AP2 is a known negative regulator in gene expression for flower development.

As the floral homeotic genes encode transcription factors, we wondered whether some of the genes differentially expressed in *grp20* flowers were due to the defects in homeotic genes. Hence, we searched 9,279 potential target genes of ABCE MADS-box proteins (usually activators) and 1,703 potential target genes of the AP2 protein (a known repressor) supported by public ChIP–seq (chromatin immunoprecipitation-sequencing) results (Extended Data Fig. 7a–d and Supplementary Table 6)^39,40^ and found 87 downregulated genes (40%) among putative MADS-box protein targets and 59 upregulated genes (12%) among putative AP2 targets (Extended Data Fig. 7e,f and Supplementary Table 6), suggesting that the splicing defects in these floral regulatory genes might have in turn caused differential expression of some of their target genes in *grp20* flowers. Therefore, GRP20 probably affects floral organ patterning by regulating splicing of nearly all ABCE floral homeotic genes. Moreover, as floral homeotic genes and GRP20 are highly conserved among flowering plants, it is possible that the role of GRP20 in the regulation of RNA splicing and flower development is conserved among at least some angiosperms.

To test the above hypothesis, we transformed *grp20-1* with fusions of the *Arabidopsis GRP20* promoter to cDNAs of *GRP20* homologues from cabbage (*Brassica rapa*, *BrGRP20*), soybean (*Glycine max*, *GmGRP20*), rice (*Oryza sativa*, *OsGRP20*) and *Amborella* (*Amborella trichopoda*, *AmGRP20*; Supplementary Fig. 3a,b). The *GRP20* homologues showed slightly lower expression levels compared with that of *AtGRP20* (Supplementary Fig. 3c); nevertheless, the *BrGRP20* transgenic plants showed almost normal flowers, and *GmGRP20* transgenic plants showed less severe defects in floral organ number and morphology when compared with *grp20* (Supplementary Fig. 3d,e), supporting the idea that *BrGRP20* is able to rescue the floral defects of the *grp20* mutant and that *GmGRP20* could partially replace the function of *AtGRP20*. However, we did not observe obvious rescue of floral defects in *OsGRP20* and *AmGRP20* transgenic plants. Furthermore, we tested whether the *BrGRP20* homologue could rescue *grp20* phenotypes in transcript splicing of floral homeotic genes using RT-qPCR in the above transgenic plants. The levels of transcripts of *AP1*, *AP3*, *SEP3* and *AP2* are similar in *AtGRP20* and *BrGRP20* flowers (Supplementary Fig. 3f). In addition, *GmGRP20* transgenic plants showed significantly decreased levels of transcripts lacking micro exons compared with those in *grp20* (Supplementary Fig. 3f). These results suggest that the *GRP20* function in flower development and RNA splicing of floral regulatory genes is probably conserved between *Arabidopsis* and cabbage.

### GRP20 binds to purine-rich motifs in micro and small exons

**Fig. 7:**
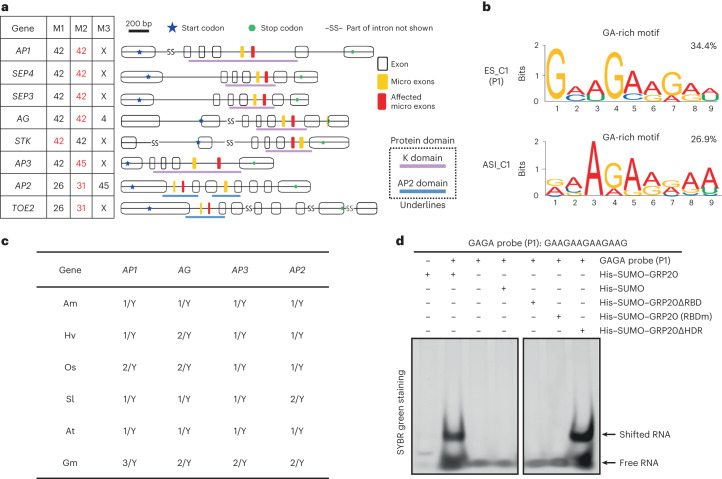
RNA consensus motifs, binding of GRP20 to GA-rich consensus and the regulation of micro-exon splicing in floral homeotic genes. **a**, An illustration of the micro exons in MADS-box and AP2 family genes. The micro exons and their sizes in MADS-box and AP2 family genes are shown in the table on the left. X indicates the absence of the micro exon. The affected micro exons are highlighted in red in gene structure diagrams on the right. The other micro exons are shown in orange, whereas other exons are shown as open boxes. Illustrations of gene structures, transcripts and reads for these floral homeotic genes and other flower developmental genes are shown in Extended Data Fig. 3. **b**, The consensus motifs and percentages found in defective transcripts, with ES and ASI defects. ES_C1 and ASI_C1 correspond to RNA probe 1, used for RNA binding. ES_C1 and ASI_C1 are the two most frequent consensuses; ASI, alternatively spliced intron. **c**, The presence of micro exons in close functional homologues (orthologues) of *AP1*, *AG*, *AP3* and *AP2* in several angiosperms. The number indicates the gene copy in this species. Y and N indicate the presence and absence of micro exons in each copy, respectively. As examples, the gene structures, including micro exons, of *AP1* and *AP2* homologues are shown in Extended Data Fig. 5h. Am, *A. trichopoda*; Os, *O. sativa*; Hv*,* *Hordeum vulgare*; Sl, *Solanum lycopersicum*; At, *A. thaliana*; Gm, *G. max*. **d**, An in vitro EMSA binding experiment using recombinant GRP20 proteins and synthetic consensus P1, as shown in Extended Data Fig. 8a. His–SUMO was used as a negative control. The mutations in the RBD and deletion of RBD were selected based on the predicted RNA-binding domain and residues shown in Extended Data Fig. 1. Source data

**Extended Data Fig. 8:**
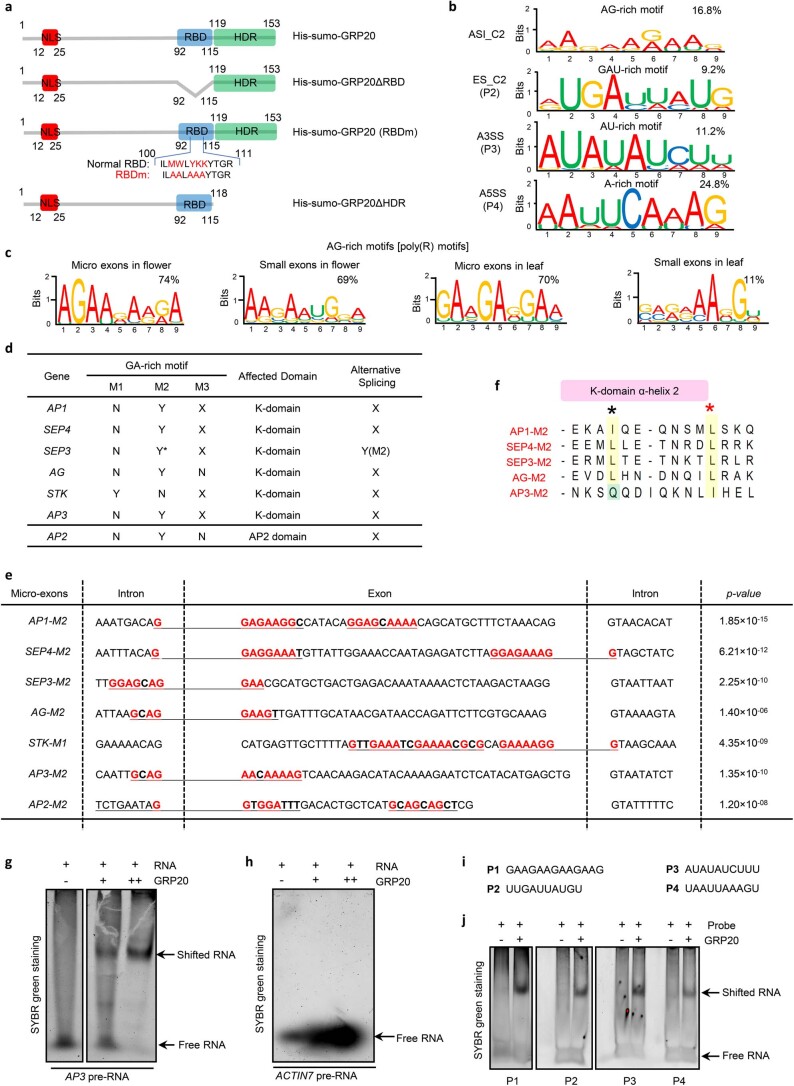
Evolutional analyses of transcripts including micro exon and small exon skipping, and *in vitro* binding between pre-mRNA or RNA motifs and GRP20. **a**, Illustrations of GRP20 constructs for expressing full-length GRP20 and recombinant proteins with the RBD mutation with five amino acids (M102A, W103A, Y105A, K106A, and K107A; RBDm) or deletion of HDR or deletion of RBD. **b**, The consensus motifs and percentages found in defective transcripts, with ES, A3SS, A5SS or ID defects. ES_C2, A3SS and A5SS correspond to RNA probe 2, 3, and 4, respectively, used for RNA binding. A5SS and ID_C2 are the second most frequent consensuses in *grp20*; A3SS: Abnormal 3’ splicing site; A5SS: abnormal 5’ splicing site. **c**, The consensus motifs and percentages found in defective micro exons and small exons in *grp20* flowers and leaves. GA-rich [poly(R)] is enriched in affected micro and small exons. **d**, The RNA motifs related to micro exons and affected protein domain in MADS-box proteins and AP2. M1, M2 and M3 indicate micro exons. Y indicates the presence and N indicates the absence of GA-rich motifs in micro exons; X indicates lack of the micro exon. An asterisk in the row for *SEP3* indicates the micro exon is skipped in an alternatively spliced transcript in WT at ~5%; however, the number of reads supporting this ES transcript is notably increased in *grp20* flowers. **e**, GA-rich motifs in affected micro-exons, as indicated by underline, with G and A residues in the motif highlighted in red. *p-*values were calculated by using MEME program with the test method named Expectation-Maximization algorithm, and are highly significant. **f**, The protein alignment of α-helix 2 in K domain encoded by affected micro-exons. Asterisks indicate the conserved key amino acid for protein tetramerization. The red asterisk indicates the most important amino acid for tetramerization formation by SEP3. **g**, *In vitro* binding of the *AP3* pre-mRNA by recombinant GRP20 using RNA EMSA. The free RNA and shifted RNA with protein are indicated. ++ indicates double the amount of input proteins (+). **h**, *In vitro* binding test of the *ACTIN7* pre-mRNA by GRP20 using RNA EMSA; the band is free RNA, indicating no detected binding. The *AP3* and *ACTIN7* pre-mRNA were synthesized by T7 *in vitro* transcription using the genomic DNAs of the corresponding gene as the template. The gels were stained by the SYBR Green fluorescent dye for RNA. **i**, The P1 to P4 probes correspond to 4 consensuses identified in the genes with splicing defects (P1: ES_C1; P2: ES_C2; P3: A3SS and P4: A5SS corresponding to Fig. 7b and Extended Data Fig. 8b). **j**, *In vitro* binding of the P1 to P4 probes by GRP20. The same amount of GRP20 proteins (17 ug) and the same amount of RNA probe (400 fmol) were added to each reaction system. The bottom bands are free RNAs, and the upper bands are shifted RNAs. GRP20 was able to bind to each of the four probes, with relatively strong binding to P1 and weak binding to P4. Source data

To learn how *GRP20* regulates flower development and RNA splicing, we tested whether the predicted RBD (Fig. 1b) is important for GRP20 function in flower development and splicing. In RBD, the W, Y, and K residues are highly conserved among angiosperms and predicted to be important for RNA binding (Fig. 3a); we generated a mutant GRP20 coding sequence (RBDm) with changes at these and other conserved residues (Extended Data Fig. 8a). We transformed the *grp20-1* mutant with a fusion of the native promoter to the *GRP20* cDNA with the RBD mutation (*Pro*_*GRP20*_*–GRP20–RBDm*), with the wild-type *GRP20* transgene (*Pro*_*GRP20*_*–GRP20*) as a positive control (Extended Data Fig. 8a). Although both *GRP20* and *GRP20–RBDm* transgenic plants showed similar protein expression levels (Fig. 6c), the *Pro*_*GRP20*_*–GRP20–RBDm* transgenic plants showed flower defects similar to those of the *grp20* mutants, unlike the *Pro*_*GRP20*_*–GRP20* transgenic plant with normal flower organs (Fig. 6d,e and Supplementary Table 5), indicating that RBD is crucial for flower development. Furthermore, we tested whether RBD is needed for RNA splicing using RT-qPCR for specific transcripts in plants carrying the *Pro*_*GRP20*_*–GRP20* or *Pro*_*GRP20*_*–GRP20–RBDm* transgenes in the *grp20-1* background. The levels of transcripts containing the relevant exons for several floral homeotic genes (for example, *AG*, *SEP4*, *AP1*, *AP2* and others; Fig. 5a) and LOB domain genes (*AS2*) were found to be reduced in transgenic plants with defective RBD, but similar to the WT in the *Pro*_*GRP20*_*–GRP20* transgenic plants (Fig. 3a), indicating that the RBD-defective transgene was not able to rescue the *grp20* phenotype of ES and alternative 5′ site transcript. In addition, the *Pro*_*GRP20*_*–GRP20–RBDm* transgenic plants exhibited a significant increase of *AP3* and *AP2* transcripts with micro-exon skipping and also alternative *LBD2* transcript with A5SS similar to those in *grp20* (Figs. 3i,j and 7a). These results indicate that the GRP20 RBD is required for flower organ patterning and RNA splicing of floral regulatory genes.

To investigate whether the regulation of splicing by GRP20 is related to sequence characteristics (motifs) in affected floral and leaf transcripts, we examined the regions of pre-mRNAs that exhibit altered splicing from 50 nucleotides upstream to 50 nucleotides downstream of the affected region (including the affected region), according to the splicing types (Extended Data Fig. 2a). The results revealed that a GA-rich consensus (poly-purine motif) was found in 45% of the skipped exons and 44% of the ASIs (Fig. 7b and Extended Data Fig. 8b), higher than the other motifs including GA-rich and A-rich motifs in introns, GAU-rich motif in exons, AU-rich motif in 3′ regions and A-rich motif in 5′ regions (Fig. 7b and Extended Data Fig. 8b), suggesting that the exonic poly-purine motifs (poly(R)) might be important for regulating splicing by GRP20. As most of the skipped exons in *grp20* flowers and leaves were micro and small exons, we examined their sequences and found that 74% of micro exons and 69% of small exons skipped in *grp20* flowers have the GA-rich motifs (Extended Data Fig. 8c). In addition, the GA-rich consensus was also found in 70% of micro exons and 11% of small exons skipped in *grp20* leaves (Extended Data Fig. 8c). To further test whether the GA-rich motif is enriched in the putative targets of GRP20, we performed an enrichment test between the affected exons in *grp20* and all annotated exons. The significant enrichment with a *P* value of 2.3 × 10^−84^ supports the idea that the poly-purine motifs are important for regulating exon retention by GRP20. In particular, the binding of GRP20 to poly-purine motifs in exons might be crucial for micro- and small-exon retention. In any case, the RBD is required for the wild-type level of transcripts containing the micro exon for MADS-box genes and *AP2* (Figs. 3a and 7a), whereas increased levels of the transcripts lacking the micro exons in *AP3* and *AP2* (Figs. 3i,j and 7a) were observed in *grp20* and RBD-defective transgenic plants. Furthermore, the affected micro exons in floral homeotic genes all contain one to two GA-rich motifs (Fig. 7a,c and Extended Data Fig. 8d,e), suggesting that the GA-rich motifs can mediate GRP20-dependent splicing of a subset of micro exons (and small exons) in floral transcripts.

To test whether the GRP20 protein with a putative RBD (Fig. 1b) can bind to RNA, including the GA-rich motif, we used an RNA electrophoretic mobility shift assay (EMSA) with the recombinant GRP20 to show that it could bind to the *AP3* pre-mRNA weakly (Extended Data Fig. 8g), but not to the *ACTIN7* pre-mRNA (Extended Data Fig. 8h). Further RNA EMSA tests indicated that GRP20 could bind to four synthetic RNA probes, with relatively high affinity for one (P1) with the GA-rich consensus (Extended Data Fig. 8i,j), similar to sequence motifs found in defective transcripts (Extended Data Fig. 8i,j). To test whether the in vitro RNA binding is dependent on the RBD, we expressed recombinant wild-type and mutant GRP20 proteins (Extended Data Fig. 8a) and tested their binding to the GA-rich probe (P1). The results showed that deletion of RBD or a mutation in the RBD blocked RNA binding by GRP20, indicating that the RBD is required for GRP20 to bind to the poly-purine motif (Fig. 7d). However, the GRP20 with deletion of the HDR could still bind to the GA-rich probe, suggesting that this domain is not crucial for RNA binding by GRP20 (Fig. 7d). The in vivo binding of GRP20 to micro-exon-containing regions of the *AP1*, *AP3*, *SEP3* and *AP2* transcripts was also confirmed by RNA immunoprecipitation (Supplementary Fig. 4a). In addition, we tested the in vitro binding of GRP20 with the GA-rich motif of the *AP1* transcript and found that GRP20 is able to bind to the *AP1* GA-rich motif, but not a mutant version with changes of three G’s to three U’s, further supporting the specific recognition between GRP20 and GA-rich RNA motifs (Supplementary Fig. 4b). The presence of one or more of the GA-rich motifs in the affected transcripts supports the hypothesis that 65% of ES and various amounts of other defects in *grp20* floral transcripts are caused by the lack of GRP20 binding directly to these transcripts; however, other defective transcripts lacking such motifs might be regulated indirectly via additional factors, such as SR1, SR33 and SmB (small nuclear ribonucleoprotein core protein), which are also affected in *grp20* (Supplementary Table 1).

### GRP20 is able to form condensates

**Extended Data Fig. 9:**
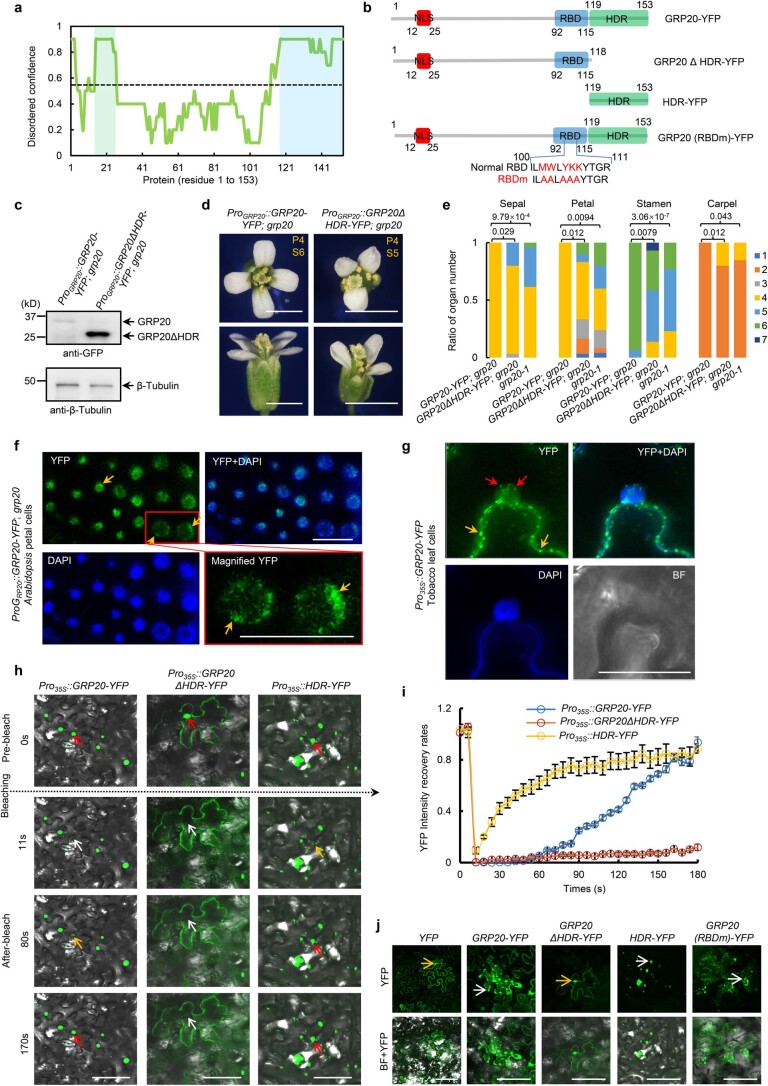
GRP20 condensates in Arabidopsis and tobacco cells, and HDR is required for flower development and condensate formation. **a**, The disorder confidence score along the GRP20 protein predicted by Phyre2. There are two highly disordered regions (60%-100%) in GRP20, one near the N-terminus (residue 15 to 25, a region with positive charge) and highlighted by light green; the other is the C-terminal region (residue 119 to 153, highly disordered region, HDR) and highlighted by light blue. **b**, Illustration of GRP20 protein structures for various transgenic constructs. **c-e**, Analyses of different transgenic plants with HDR constructs, as shown in Extended Data Fig. 9b. (**c**) Comparison of protein expression level in transgenic plants, using Western blot with antibodies against GFP and the same amount of input flower proteins indicated by antibodies against β-Tubulin. The GRP20ΔHDR transgenic plants expressed the GRP20 protein at a level higher than that in the wild-type. (**d**) Flower phenotypes in transgenic plants as labeled at the top. Yellow letters and numbers at the right top of each panel indicate the organ and corresponding number. P: Petal; S: Stamen. Bar = 1 mm. (**e**) Floral organ numbers in transgenic plants. Flower counts: *Pro*_*GRP20*_*::GRP20-YFP grp20*, 30; *Pro*_*GRP20*_*::GRP20ΔHDR-YFP grp20*, 30 and *grp20-1*, 26. Data are presented as mean ± SD. Two-sided Student’s *t* test. **f**, The GRP20 condensates in *Arabidopsis* petal nuclei in complementation lines. The nuclei were stained by DAPI. The condensates are indicated by yellow arrows. BF: Bright field. The bottom right panel is a magnified image of red boxed area in the top left panel. Bar= 5 μm. **g**, GRP20 condensates in a tobacco leaf cell following transient transformation. The nucleus is also stained by DAPI. Red arrows indicate some of nuclear condensates and yellow arrows indicate some of the condensates in the cytosol. Bar= 5 μm. **h**, The liquid fluidity of GRP20 condensates tested by photo-bleaching in tobacco cells. The YFP fluorescent signal was recorded from 0 second(s) to 180 s. The YFP condensates were bleached by 100% 514 nm laser from 7 to 11 s after the start of recording. Red arrows mark the positions of strong YFP fluorescence signals, yellow arrows indicate weak YFP signals (following bleaching), and white arrows indicate the positions corresponding to the pre-bleach YFP signals. **i**, Fluorescence intensity recovery of YFP signals from the experiments shown in Extended Data Fig. 9h. HDR: highly disordered region. The intensity data are shown by mean ± SD from 15, 10 and 20 condensates for GRP20-YFP, GRP20ΔHDR-YFP, and HDR-YFP, respectively. Bar= 10 μm. **j**, Condensate formation of various GRP20 proteins with/without deletion of HDR, HDR alone, or RBD mutations, respectively, in tobacco leaf epidermal cells. The condensates were detected in GRP20-YFP, HDR-YFP, and GRP20 (RBDm)-YFP, the signal from GRP20ΔHDR-YFP was relatively even, without obvious condensate. Results support the idea that HDR is necessary and sufficient for condensate formation, but RBD is not necessary to condensate formation. The yellow arrow indicates the nucleus, and the white arrow indicates the condensate, respectively. Bar= 5 μm. Source data

GRP20 contains an HDR (Fig. 1b and Extended Data Fig. 9a), which is annotated by the disorder confidence program, rich in proline and alanine residues and highly hydrophilic^41^. To investigate the role of the HDR in vivo for flower development and RNA splicing, the transgenic plants were generated that contain a fusion of the *GRP20* promoter to the *GRP20* cDNA lacking the HDR (*GRP20ΔHDR*) in the *grp20* background (Extended Data Fig. 9b). Although the *GRP20ΔHDR* transgenic plants expressed the GRP20 protein at a level higher than that in the wild type (Extended Data Fig. 9c), they showed floral phenotypes similar to those of *grp20* (Extended Data Fig. 9d,e and Supplementary Table 5), suggesting that the HDR is needed for normal flower development. In addition, the *GRP20ΔHDR* transgenic plants in the *grp20* background showed the reduced levels of transcripts containing the relevant exons for several floral homeotic genes (for example, *AG*, *SEP4*, *AP1*, *AP2* and others) and LOB domain gene (*AS2*), similar to those in *grp20* and the *GRP20 (RBDm)* transgenic line (Fig. 3a). Moreover, the *GRP20ΔHDR* transgenic plants also exhibited a significant increase in the level of *AP3* and *AP2* transcripts lacking the relevant micro exon and also the alternative *LBD2* transcript with A5SS similar to those in *grp20* (Fig. 3j). These results further suggest that the HDR is also important for RNA splicing during flower development.

HDRs have been linked to liquid–liquid-phase separation (LLPS)^42^, which involves the formation of condensates of proteins or other macromolecules^43^ and can be visualized as punctate signals in the cell^44^, leading to the hypothesis that GRP20 can also form condensates. We found that the GRP20–YFP (yellow fluorescent protein) fusion protein formed condensates in *Arabidopsis* petal cells and tobacco epidermal cells (Extended Data Fig. 9f,g). Then, we showed that the GRP20–YFP condensates could be restored following photo bleaching (Extended Data Fig. 9h,i). Moreover, a fusion protein of the HDR with YFP also formed condensates, which was restored after the photo bleaching (Extended Data Fig. 9h,i). In addition, the observations of condensate formation of GRP20 (RBDm)–YFP (Extended Data Fig. 9j) and RNA binding of GRP20ΔHDR (Fig. 7d) support the idea that RNA binding and condensate formation are separate activities of GRP20.

### GRP20 interacts with the U5 subunits of the spliceosome

**Fig. 8:**
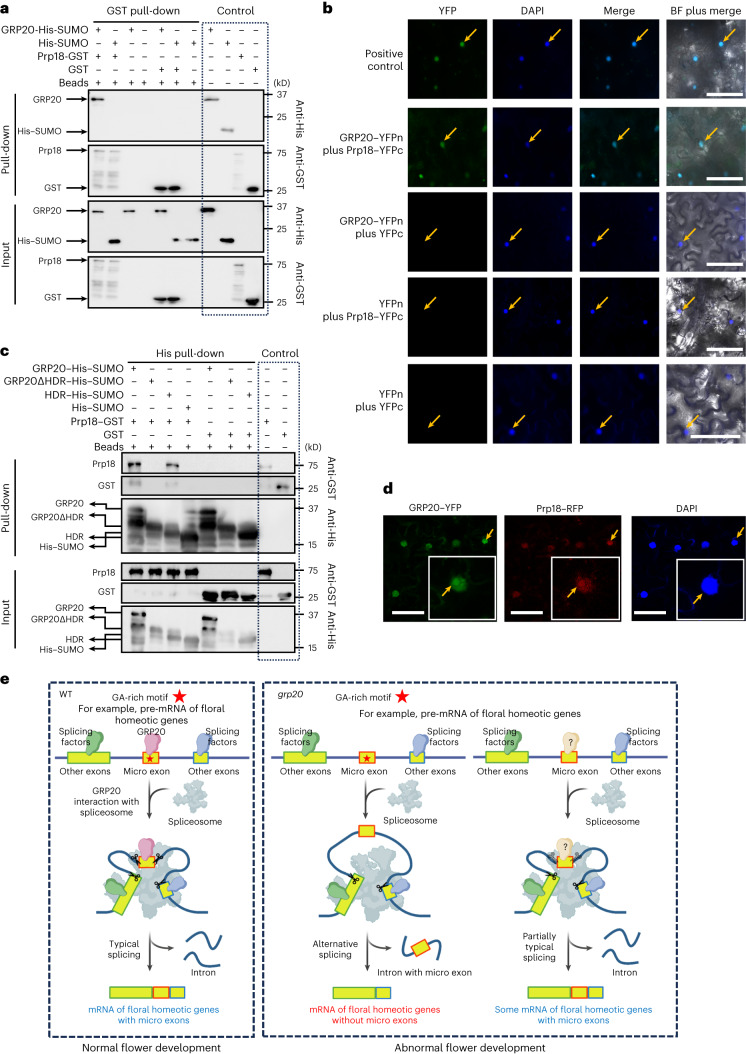
The interaction of GRP20 and spliceosome in vitro and in vivo, and a model showing the mechanism of GRP20 in RNA splicing. **a**, Detection of GRP20 and Prp18 interaction by GST pull-down assay in vitro. The full-length Prp18 was tagged with GST, and GRP20 was tagged with SUMO–His. His–SUMO and GST were used for negative controls. Input and GST pull-down samples were analysed by western blot using antibodies against His and GST, respectively. **b**, Examination of GRP20 and Prp18 interaction in tobacco leaves by BiFC. Co-expression of a fusion of the YFP-N-terminal region (YFPn) with GRP20 and that of the YFP-C-terminal region (YFPc) with Prp18 resulted in strong YFP fluorescence (green in the left row) in the nucleus similar to the positive control, indicating a positive interaction between GRP20 and Prp18 in vivo. Co-expression of GRP20 or Prp18 with blank YFPc or YFPn was performed as the negative control. The nuclei are indicated by DAPI (second row). The third panel (cyan) merges DAPI (blue) with YFP (green). BF, bright field. Scale bars = 20 μm. **c**, Examination of truncations of GRP20 and Prp18 by His pull-down assay. The truncations of GRP20 (GRP20ΔHDR and HDR) were tagged with His–SUMO. Input and GST pull-down samples were analysed by western blot using antibodies against His and GST, respectively. The interaction of GRP20 and Prp18 was dependent on HDR. **d**, The co-localization of GRP20–YFP and Prp18–RFP in nuclear condensates in tobacco leaf cells. The nuclei were stained with DAPI. The yellow arrows indicate the condensates in the nuclei. Scale bars = 10 μm. **e**, In WT cells (left), GRP20 RBD interacts with a GA-rich motif (red star) in some micro and small exons of specific pre-mRNAs, such as floral homeotic transcripts. Furthermore, the C-terminal portion of GRP20 interacts with the spliceosome to promote retention of micro and small exons during RNA splicing, thereby supporting normal flower development. In *grp20* cells (right), the recognition of micro and small exons and spliceosome interaction is reduced owing to the absence of GRP20, leading to an increase of transcripts lacking the micro and small exons. However, unknown factors might bind to pre-mRNAs and deliver pre-mRNAs to the spliceosome for splicing; therefore, in the absence of GRP20, both typical and alternatively spliced transcripts are produced. The reduced levels of floral homeotic transcripts lead to abnormal flower development. Panel **e** was created with BioRender.com and Microsoft PowerPoint 2021. Source data

**Extended Data Fig. 10:**
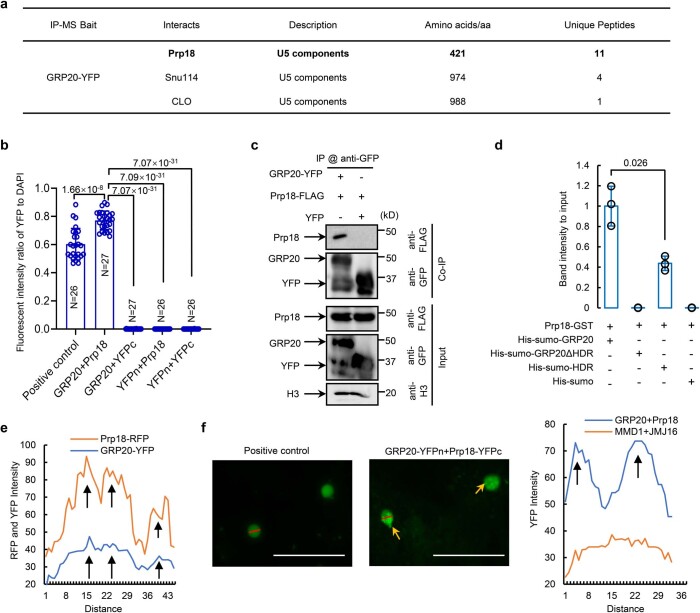
The interaction between GRP20 and spliceosome in the condensates depending on HDR. **a**, The spliceosome U5 subunits identified by IP-MS. **b**, The quantification of YFP signals in the nucleus of tobacco leaves in Fig. 8b. The fraction of YFP intensity to DAPI intensity for a single nucleus was estimated. The data were presented as mean ± SD from 26 or 27 nuclei for each transient experiment indicated in each column. PC: positive control. **c**, Co-IP of GRP20 and Prp18 from *Arabidopsis*. GRP20-YFP and Prp18-FLAG fusion proteins driven by the CaMV-35S promoter were co-expressed in *Arabidopsis*. Seedlings from such transgenic plants were used to extract nuclear proteins for immunoprecipitation using GFP antibody-conjugated agarose beads. The pre-IP and IP samples were analyzed by immunoblotting using antibodies against FLAG and GFP, separately. *Arabidopsis* co-expressing GFP and Prp18-FLAG driven by the 35 S promoter was used as a negative control. The pre-IP sample using an antibody against Histone 3 was the loading control. **d**, The band intensity of Prp18-GST in His pull-down experiments for GRP20, GRP20ΔHDR and HDR in Fig. 8c. His-sumo is a negative control. Nearly none of Prp18 was captured by GRP20ΔHDR and 40% of Prp18 was captured by HDR, compared to full-length GRP20. The experiments are conducted by three independent replicates with similar results. The data were presented as mean ± SEM. **e**, The fluorescent intensity of Prp18-RFP and GRP20-YFP estimated in the drawing line across the nucleus (left to right distance shown in the X-axis) in Fig. 8d by Image J. The data suggested that Prp18-RFP and GRP2-YFP are co-localized in the nuclei and have stronger co-localization in three condensates in the nucleus indicated by black arrows. **f**, The condensate formation in the nucleus showing interactions between GRP20 and Prp18 by BiFC assay. The MMD1 and JMJ16 were used as a control that do not form condensates under interaction. The yellow arrows indicate condensates in the tobacco nuclei when GRP20-YFPn interacts with Prp18-YFPc. The fluorescent intensity of YFP after interactions in the right panel was estimated by drawing red lines across the nucleus (left to right distance shown in the X-axis) in the left panel. YFP signals represented positive interaction. Two obvious peaks indicated by black arrows were observed in the interaction nucleus of GRP20 and Prp18 suggesting two condensates, however, none of peaks (condensates) were observed in positive interacting nucleus. Two-sided Student’s *t* test is used for statistics in 10b and 10d. Source data

Our results suggest that GRP20 can bind to some micro exons and small exons in pre-mRNAs in part through poly-purine motifs and probably recruits specific pre-mRNAs for splicing. To test whether known components of spliceosome machinery and regulators can bind to GRP20, we performed immunoprecipitation–mass spectrometry with a GRP20–YFP protein expressed in plants. Among putative interactive proteins, spliceosome U5 components Prp18, Snu114 and CLO were identified (Extended Data Fig. 10a). Importantly, U5 is a highly conserved core subunit of the spliceosome^7^. The physical interaction between GRP20 and Prp18 was further confirmed in vitro by a GST pull-down assay (Fig. 8a), by bimolecular fluorescence complementation (BiFC) in tobacco cells (Fig. 8b and Extended Data Fig. 10b) and by co-immunoprecipitation in *Arabidopsis* plants (Extended Data Fig. 10c). To investigate which part of GRP20 is required for interaction with Prp18, we generated truncated proteins (Extended Data Fig. 8a) and found that deletion of the HDR completely disrupted the interaction with Prp18 and that the HDR could bind to Prp18 weakly (Fig. 8c and Extended Data Fig. 10d), suggesting that HDR is crucial for the interaction. Furthermore, we obtained co-localization of GRP20 and Prp18 in the tobacco nucleus. The co-localization signals were particularly strong in the nuclear condensates (Fig. 8d and Extended Data Fig. 10e), and the stronger YFP signals were also observed in nuclear condensates from the interaction between GRP20–YFPn and Prp18–YFPc (Extended Data Fig. 10f). Such interactions among GRP20, the spliceosome and pre-mRNAs, especially those containing micro and small exons, probably facilitate splicing of these RNAs.

To examine whether the interaction between GRP20 and Prp18 is important for GRP20 function in flower development and proper retention of micro exons in floral homeotic genes, we generated a GRP20 deletion mutant (GRP20Δ143–153) lacking the C-terminal 11 amino acid residues and a mutant *GRP20* cDNA (HDRm) with changes at lysine and proline residues (Supplementary Fig. 5a). These mutant GRP20 proteins failed to interact with Prp18 (Supplementary Fig. 5b). In contrast, both mutant GRP20 proteins could still form condensates (Supplementary Fig. 5c), providing a means to test the role of GRP20 interaction with Prp18 without affecting condensate formation. We transformed the *grp20-1* mutant with a fusion of the native promoter to the *GRP20* cDNA with the HDR mutation (*Pro*_*GRP20*_*–GRP20–HDRm*), with the wild-type *GRP20* transgene (*Pro*_*GRP20*_*–GRP20*) as a positive control (Supplementary Fig. 5a,d). Although both *GRP20* and *GRP20–HDRm* transgenic plants showed similar protein expression levels (Supplementary Fig. 5d), the *Pro*_*GRP20*_*–GRP20–HDRm* transgenic plants showed flower defects similar to those of the *grp20* mutants, unlike WT plants (Supplementary Fig. 5e,f), indicating that the interaction to Prp18 (component of U5 of the spliceosome) is crucial for GRP20 function in flower development. Furthermore, we tested whether the interaction to the spliceosome is needed for RNA splicing using RT-qPCR for specific transcripts in plants carrying the *Pro*_*GRP20*_*–GRP20–HDRm* transgene in the *grp20-1* background. The *Pro*_*GRP20*_*–GRP20–HDRm* transgenic plants exhibited significantly increased levels, compared with the WT, of *AP1*, *AP3* and *AP2* transcripts lacking the micro exons that were skipped in the *grp20* mutant, similar to those in *grp20*; also, the levels of alternative *AS2* transcript with A5SS were similar in *Pro*_*GRP20*_*–GRP20–HDRm* transgenic plants and *grp20* (Supplementary Fig. 5g). These results indicate that the interaction between GRP20 and the spliceosome is required for flower organ patterning and RNA splicing of floral regulatory genes.

On the basis of results here, we propose a model with specific mechanisms of GRP20-mediated micro-exon (and small-exon) retention (Fig. 8e). GRP20 specifically binds to micro-exon-containing regions of floral homeotic transcripts and facilitates the proper retention of the micro exons by interacting with Prp18, a component of the U5 portion of the spliceosome. Furthermore, the observations that micro exons of floral homeotic genes were retained, albeit at reduced levels, in *grp20* mutants and that most micro exons were retained indicate that there are probably other factors for micro-exon (and small-exon) splicing (Fig. 8e).

## Discussion

Pre-mRNA splicing depends on interactions with the spliceosome, splicing factors and regulators^45^. For most introns, the GU-AG nucleotides at the ends of the intron are involved in spliceosome binding and promote accurate splicing; also, the sequence information in typical exons with an average length of 150 nucleotides in vertebrates and 180 nucleotides in plants facilitates the binding of general splicing factors^6,17^. However, micro exons usually lack sequence motifs for binding by general splicing factors and probably require additional factors. In this study, we identified a highly conserved RNA-binding protein, GRP20, in angiosperms that functions in RNA splicing, including the proper splicing of micro and small exons. GRP20 can bind to RNAs containing GA-rich and other motifs in micro and small exons and other exons of pre-mRNA through an RBD recognized here, thereby facilitating the proper splicing of subsets of genes that are expressed in the flower and/or leaf. This is the first report of a specialized splicing regulator of genes with known or predicted functions in plant development and environmental responses. Furthermore, GRP20 interacts with specific pre-mRNAs through its RBD and with the spliceosome involving its C-terminal portion; GRP20 might coordinate with the spliceosome machinery and pre-mRNAs to promote typical RNA splicing (Fig. 8e). Moreover, GRP20 is the first identified eukaryotic regulator of micro-exon and small-exon splicing with a newly recognized domain for binding to exonic poly-purine motifs and likely interaction with the spliceosome component. Overall, our results provide new mechanistic insights into the regulation of plant gene expression, at the level of RNA splicing, for genes important for flower development and possibly other processes.

In *Arabidopsis*, mutants defective in the splicing factors *SC35* and *SR45* genes encoding SR proteins exhibit intron retention defects in many transcripts and ES in fewer transcripts^46,47^, but whether they play roles in micro-exon and small-exon splicing is not known. SC35 and SR45 can bind to purine-rich (GA) motifs in introns, not exons^46,47^, and SR45 also binds to pyrimidine-rich motifs^48^, but the role of RNA-binding activities of these proteins in splicing has not been tested in vivo, nor is additional information about their mechanisms available. The binding of plant SRs and other RNA-binding proteins to exons with a particular size range (micro and small exons or other sizes) has not been reported. GRP20 is capable of binding to micro exons and acts as a eukaryotic regulator of small exon splicing; the binding to poly-purine motifs in the exons is a newly reported mechanism for exon retention in eukaryotes. Moreover, the skipping of the micro exon could be reduced when purines in the intronic poly-pyrimidine tract are replaced by pyrimidines^19^. Therefore, a single or a few nucleotide changes between purine and pyrimidine might lead to RNA splicing defects, resulting in mutant proteins, providing an explanation for alternative RNA splicing associated with SNPs.

In *Arabidopsis*, ~5.5% of exons (8,118 of 150,240) are micro exons and ~25.9% (39,025 of 150,240) are small exons (Fig. 2d); moreover, ~16.3% of *Arabidopsis* genes (5,693 of 35,000) contain micro exons and ~35.8% (12,525 of 35,000) contain small exons (Extended Data Fig. 5). In addition, ~23% of rice genes possess micro exons^26^. These data support the idea that micro and small exons are important gene-structural elements in plants, as proposed for micro exons in humans^23^. The gene structures of *AP1*, *AP3*, *AG* and *AP2* homologues in diverse angiosperms, including the early-divergent *Amborella*, monocots barley and rice, and eudicots tomato and soybean, all contain the micro exons corresponding to those that are skipped in *grp20* floral transcripts (Fig. 7c and Extended Data Fig. 5h), suggesting that the proper splicing of these micro exons is conserved and probably important for flower development across angiosperms. The combination of GRP20 and micro exons in floral homeotic genes might be a key component in the regulatory programme for flower development during angiosperm evolution.

*Arabidopsis* has other GRPs related to GRP20; to test whether some of them also have some functions similar to those of GRP20, in flower development and RNA splicing, we investigated closely related *GRP20* paralogs *GRP17*, *GRP19* and *GRP21*, and a more distant gene *GRP7* (Supplementary Figs. 1 and 6), using their corresponding T-DNA insertion mutants (Supplementary Fig. 6a). Although the RNA expression levels of each gene were significantly decreased in the corresponding mutant (Supplementary Fig. 6b), the mutants showed similar floral phenotypes to the WT (Supplementary Fig. 6c,d). In addition, the *AP1*, *AP3*, *SEP3* and *AP2* transcripts lacking the micro exons affected in the *grp20* mutant were not detected in *grp17*, *grp19*, *grp21* and *grp7* flowers, unlike the floral and splicing phenotypes of *grp20* (Supplementary Fig. 6e–h). These results and the differences in protein domains suggest that GRP17, GRP19, GRP21 and GRP7 probably do not play similar roles to GRP20 in flower development and RNA splicing.

Furthermore, nearly all of the micro exons in MIKC-type MADS-box genes encode a part of the K domain^27^, which allows the formation of multimeric complexes of MADS-box proteins as the molecular basis for the floral quartet model^49^. The crystal structure and biochemical studies of SEP3 showed that the N- and C-terminal regions of the second amphipathic helix encoded by the two micro exons in *SEP3* are essential for dimerization and tetramerization, respectively^29^. In our study, all affected micro exons in ABCE family genes encode the C-terminal region of the second helix, similar to that in SEP3, and share leucine and isoleucine residues for tetramer formation (Extended Data Fig. 8f), suggesting that the proper retention of the micro exons is essential for the multimeric complex, an essential aspect of the quartet model for flower development. It was found that a circular RNA containing the second micro exon of *SEP3* increased the level of the splicing variant without the second micro exon^30^, and the overexpression of this *SEP3* splicing variant induced changes in petal and stamen number similar to those of *grp20* (ref. ^30^), supporting the idea that the micro exon is important for normal floral organ patterning. Although GRP20 is crucial for proper retention of micro exons in floral developmental genes, GRP20 affects a portion of micro and small exons in floral and leaf transcripts (Fig. 3d,e), suggesting that other factors are needed to regulate splicing of other micro and small exons and biological processes. In addition, *GRP20* also promotes the retention of longer exons and affects 5′ and 3′ splicing junctions in some transcription factor family genes, LOB domain genes and other developmental and responsive genes. These splicing defects might also contribute to abnormal organ shape, blue dots on petals and other abnormal floral phenotypes in *grp20* flowers. Further studies are required to investigate the possible role of GRP20 in other plant development and response processes. We showed that GRP20 can form condensates and that the HDR of GRP20 is required for condensate formation, suggesting that GRP20-dependent splicing might involve LLPS. Condensate formation and LLPS have been implicated in RNA metabolism including splicing^43^ and regulation of gene expression^50^; such processes have also been suggested to involve LLPS in plants^51,52^. Our results here on GRP20 and the general presence of micro and small exons in plant genes suggest that splicing regulators specialized for micro and small exons are probably important for normal gene expression.

## Methods

### Plant materials and growth conditions

The *Arabidopsis thaliana* mutants used in this study were obtained from the *Arabidopsis* Biological Resource Center and are as follows: *grp20-1* (SALK_134093), *grp20-2* (SALK_026077), *ap1-1* (CS127), *ap2-1* (CS148), *pi-1* (CS77), *ag-1* (CS25), *as2-1/lbd6-1* (CS3117), *lbd7-1* (SALK_075629), *grp17-1* (SALK_133589), *grp19-1* (SALK_034288), *grp19-2* (CS923713), *grp21-1* (SALK_032127), *grp21-2* (SALK_127070) and *grp7-1* (SALK_039556). The single mutants were crossed with Col-0, and the genotypes were identified by PCR using corresponding primers listed in Supplementary Table 7. The double mutants were generated by relevant crosses and identified in the F2 generation by PCR. *Arabidopsis* and *Nicotiana benthamiana* were grown in a plant growth room at 21 °C, with a 16 h light and 8 h dark photoperiod and 60% humidity.

### Bioinformatic analyses of GRP20 protein domain

The predictions of potential RNA-binding ability and RNA-binding regions in GRP20 were conducted by using the software catRAPID^53^ and RNAbindPlus^54^. The category of RNA-binding protein for GRP20 was also predicted by using the catRAPID signature program. The putative RNA-binding residues were identified by using two programs: DRNApred^55^ and PPRInt^56^. The cut-off values for catRAPID, RNAbindPlus, DRNApred and PPRInt were 0.5, 0.1, 0.05 and −0.2, respectively.

The protein disordered confidence analysis was performed using Phyre2 with structure prediction. The cut-off in this program for the protein disordered confidence was 0.6. The ‘ParSe: Predict Phase-Separating Protein Regions from the Primary Sequence’ program^57^ (http://folding.chemistry.msstate.edu/utils/parse.html) was used in the prediction for the LLPS of GRP20 homologues among angiosperms. The disorder confidence scores along HDRs and whether HDRs can undergo LLPS of GRP20 homologues are shown by *V*_model_ and *β*_model_ (*β*-turn propensity). *V*_model_ < 0.56 and *β*_model_ > 0.9 indicate that the domain is intrinsically disordered and prone to undergo LLPS or fold to a stable conformation.

### Plant phenotypic analyses

Flowers from WT, mutants and transgenic lines were examined. Six T3 lines were characterized for the complementation experiment. The number of flowers for various statistical analyses of phenotypes is indicated in the figure legends. The chimeric organs and other defects were shown as examples of mutant phenotypes. Petal morphology including length, top angle and width was measured in the same way for WT, mutants and transgenic plants. Flower photographs were obtained using a Nikon microscope (SMZ-U) and an AmScope microscope digital camera (catalogue number MU1803-HS); the numbers of flower organs were counted using the same Nikon microscope, in four whorls. The chimeric organs of the petal and stamen in the second whorl were counted as petals, whereas the chimeric organs of the petal and stamen in the third whorl were counted as stamens. For scanning electron microscope observations, a fresh unopened single flower was prepared, and the sepals were removed using needles. The scanning electron microscope photographs were taken using a variable-pressure detector in a Zeiss SIGMA VP-FESEM under 10 kV.

### Confocal image analyses

For the observation of YFP protein and other confocal images, stable transgenic lines were used to provide fresh whole flowers or organs including petals, sepals and anthers, which were then used for confocal imaging (LSM880, Zeiss). DAPI (4′,6-diamidino-2-phenylindole, catalogue number 14285; 0.05 mg ml^−1^) was used for nuclear staining. For transient transformation and protein expression, leaves of 4- to 6-week-old *N. benthamiana* were infiltrated by *Agrobacterium* GV3101 containing corresponding plasmids and grown in the dark for 24 h and then in the light for the following 24 h. The images of bottom (abaxial) epidermal cells were obtained using a Zeiss LSM880 confocal microscope or an Olympus FV1000 confocal microscope. The DAPI and YFP signals were captured, respectively, under 405 nm and 514 nm lasers in similar gain settings.

### Transcriptomic analyses and qRT-PCR

For each of three biological replicates, *Arabidopsis* stage 1–12 flowers were collected separately from Col-0 and *grp20*. The RNA extraction and analyses were executed as described previously^58^. RNA sequencing (RNA-seq) was conducted using an Illumina NextSeq 2000 instrument with 2 × 150 bp paired-end outputs. The statistical significance of RNA-seq data was calculated using the *q* value (an adjusted *P* value) cut-off <0.05 and |fold-change| ≥ 2 in the DESeq2 package. Upregulated genes in transcriptomic data were categorized using log_2_(fold change) ≥ 1, *q* value < 0.05, and downregulated genes were categorized using log_2_(fold change) ≤ −1, *q* value < 0.05 in Supplementary Table 2. Venn maps were generated using Venny (version 2.1.0) and R software (version 3.5.2). GO enrichment analysis was conducted using Gene Ontology, and statistics were compiled by FDR (false discovery rate) correction and Fisher’s test. The heat maps and volcano diagrams were generated using Origin (version 2020b) and R (version 3.5.2) software. The primers used for gene expression estimates were designed using qPrimerDB^59^ and Primer3Plus, and listed in Supplementary Table 7. qRT-PCR was performed as described previously^60^, with three biological replicates. The GoTaq qPCR and RT-qPCR systems (catalogue number A6010, Promega) were used for reverse transcription and qPCR. The qRT-PCR experiments were performed using Applied Biosystems StepOnePlus real-time PCR systems (catalogue number 4376600, ThermoFisher) with standard PCR procedure.

### Analyses of RNA splicing and detection of affected transcripts by RT-qPCR

RNA splicing analyses were conducted based on multivariate analysis of transcript splicing assay (rMATS version 4.1.1)^61^, and the results were verified manually in Integrative Genomics Viewer (IGV version 2.9.4). RNA-seq data from WT and *grp20* stage 1–12 flowers with three replicates were used in the analyses. The Hisat2 program is used to map reads for the analyses of splicing as referenced in plants^25–27^ and animals^62^. In our analyses, 2 × 150 bp paired-end read sequences were used and the average usable read length is 130 bp after removing the adaptor sequences. In addition, the annotated *Arabidopsis* gene structures were used as a reference in the mapping process, such that annotated micro exons and small exons in the transcript reads have a low probability of being not detected bioinformatically. The read maps and counts were illustrated based on the IGV output. The reference for gene structures used as the input in IGV was the TAIR10 GFF3 file, which was downloaded from the TAIR website (https://www.arabidopsis.org/download/index.jsp). For *P* value calculation as referenced^61,63^, the rMATS program was used with a hierarchical framework to simultaneously account for estimation uncertainty in individual replicates and variability among replicates (https://github.com/Xinglab/rmats-turbo/blob/v4.1.2/README.md). rMATS uses a hierarchical framework to model exon inclusion levels, similar to percent spliced in (PSI), which is shown in Supplementary Table 1. The illustration of gene structure and support reads in Extended Data Fig. 3 for each transcript was according to the results from IGV. Detailed information on read numbers, TAIR gene IDs and, if available, gene names and symbols is provided in Supplementary Table 1. qRT-PCR was used to confirm the defects in splicing. The primers used to detect the WT transcripts in affected regions are shown in Supplementary Table 7, and the primers used for the detection of transcripts with micro-exon skipping or A5SS are indicated in Fig. 3i and Supplementary Table 7. The definition of micro exon (<51 nucleotides) is based on a previous study^24^, and the small exon (51–100 nucleotides) is defined in this study as it is larger than micro exons and smaller than average exons. The short exons (<101 nucleotides), including micro exons (<51 nucleotides) and small exons (51–100 nucleotides), were identified by searching the *Arabidopsis* genome (TAIR10) and categorized into four groups by size: 1–9 nucleotides, 10–25 nucleotides, 26–50 nucleotides and 51–100 nucleotides.

Although the rMATS program can detect statistical significance in AS between WT and *grp20*, it is not very sensitive in detecting intron retention; therefore, to obtain additional evidence for intron retention, we analysed our datasets using the SUPPA2 tool^64^. In flowers, 3,712 genes were found to have intron retention by the SUPPA2 tool, including 887 genes detected by the above analyses and 2,825 additional genes (see details in Supplementary Table 1).

### RNA motif analyses, RNA binding using EMSA and RNA immunoprecipitation

RNA motif analyses were carried out using the MEME program (version 5.4.1)^65^ for transcripts with different types of splicing defects. The sequences from 50 nucleotides upstream to 50 nucleotides downstream of the affected regions (including the affected region) in the flower-specific group of transcripts according to the splicing types were included as inputs for consensus detection. Single-strand RNA probes corresponding to consensus motifs were synthesized by Integrated DNA Technologies. The RNA powder was diluted in RNase-free water to 200 fmol μl^−1^. EMSA for RNA binding was performed as previously described^66^. To transcribe full-length pre-mRNA for EMSA, the DNA templates of *AP3* and *ACTIN7* were amplified using PCR with the Q5 High-Fidelity DNA Polymerases (catalogue number M0491, New England BioLabs), with *Arabidopsis* genomic DNA and one of the primers fused to the promoter for T7 phage polymerase (TAATACGACTCACTATAGGGAGA). The *Arabidopsis* genomic DNA was extracted using the CTAB assay and further purified using a genomic DNA clean and concentration kit (catalogue number D4010, ZYMO RESEARCH). The DNA templates of *AP3* and *ACTIN7* were recovered from agarose gel using a NucleoSpin Gel and PCR Clean‑up kit (catalogue number 740609, MACHEREY-NAGEL). The primers are listed in Supplementary Table 7. The *AP3* and *ACTIN7* pre-mRNAs were transcribed in vitro with the corresponding DNA template using the T7 phage polymerase and other reagents in the MAXIscript SP6/T7 transcription kit (catalogue number AM1322, ThermoFisher). The RNA transcripts were treated with DNase I and purified using an RNA clean and concentration kit (catalogue number 1015, ZYMO RESEARCH). Purified GRP20 (1 mg or 2 mg) was incubated with 500 ng purified pre-mRNA in 1× EMSA buffer (10 mM HEPES, 150 mM NaCl, 3 mM EDTA, 2 mM DTT, 10% glycerol, 1 mM PMSF) at room temperature for 30 min, respectively. Binding to RNA probe using EMSA was conducted using an EMSA kit (catalogue number E33075, Molecular Probes), including incubation of 400 fmol RNA probes and 17 μg GRP20 proteins in 1× binding buffer at room temperature for 30 min. The total samples with the RNA EMSA loading buffer were loaded onto a 5% polyacrylamide native gel, after a pre-run of 1 h at 45–60 V. The gel was run for about 1 h at 6–15 mA and then was stained using SYBR Green EMSA nucleic acid gel stain (1:10,000 dilution in 0.5× TBE (RNase-free water)) in the EMSA kit (catalogue number E33075, Molecular Probes), and the stained RNA was detected and recorded using a ChemiDoc image system (Bio-Rad).

RNA immunoprecipitation was performed as referenced^67^. The whole inflorescences and leaves were collected from *Pro*_*GRP20*_*::GRP20-eYFP* and *Pro*_*35S*_*::eYFP* transgenic plants. The nuclei of the samples were preliminarily obtained with extraction buffer (20 mM Tris–HCl pH 7.5, 150 mM NaCl, 2.5 mM MgCl_2_, 0.5% Triton X-100, 10% glycerol, 0.5 mM DDT, 2 mM PMSF and 20 U ml^−1^ RNase inhibitor) including RNase inhibitor (catalogue number AM2696, Invitrogen). The anti-GFP antibody (mAb: catalogue number AE012, ABclonal, 1:50) and protein G magnetic beads (catalogue number S1430S, New England BioLabs) were added into the total nuclear RNA lysis for overnight. The protein A/G magnetic beads were washed at least three times with washing buffer (20 mM Tris–HCl pH 7.5, 150 mM NaCl, 2.5 mM MgCl_2_, 0.2% Triton X-100, 10% glycerol, 0.5 mM DDT, 1× protease inhibitor cocktail and 20 U ml^−1^ RNase inhibitor) and dilution buffer (20 mM Tris–HCl pH 7.5, 150 mM NaCl, 2.5 mM MgCl_2_, 10% glycerol, 1 mM PMSF and 20 U ml^−1^ RNase inhibitor). The precipitated complexes were resuspended by protease buffer and treated with RNase inhibitor and proteinase K. Homogenization buffer (100 mM Tris–HCl pH 8.0, 5 mM EDTA pH 8.0, 100 mM NaCl, 0.5% SDS and 0.01 volume β-ME) was added to the precipitated complexes, and RNA was extracted using the phenol–chloroform–isoamyl alcohol method. cDNA was synthesized with purified RNA and ABScript III RT master mix for qPCR with a gDNA remover kit (catalogue number RK20429, ABclonal). Then, qPCR was performed as described previously^60^, with three biological replicates. The 2× Universal SYBR green fast qPCR mix systems (catalogue number RK21203, ABclonal) were used for qPCR. The qRT-PCR experiments were performed using Applied Biosystems StepOnePlus real-time PCR systems (catalogue number 4376600, ThermoFisher) following the instructions.

### Recombinant protein purification

The CDSs of *GRP20*, *GRP20ΔHDR* (residue 1 to 118), *HDR* (residue 119 to 153) and *GRP20Δ143-153* (residue 1 to 142) were cloned into pSUMO (pET28a–SUMO) vector between the BamHI and XhoI restriction sites. The coding sequences of GRP20ΔRBD (deletion of residue 92 to 115), GRP20 (RBDm) (M102A, W103A, Y105A, K106A and K107A) (m, mutation with indicated amino acid changes) and GRP20 (HDRm) (K143A, P144A, P147A, K150A and P151A) were also cloned into pSUMO (pET28a–His–SUMO) and then mutated using a Q5 site-directed mutagenesis kit (catalogue number E0552S, New England BioLabs). The CDSs of the primers for the mutagenesis experiment were designed using NEBaseChanger (New England BioLabs) and listed in Supplementary Table 7. The plasmids were transformed into *Escherichia coli* Rosetta (DE3). The positive strains were grown at 37 °C to OD = 0.6, then transferred to 18 °C for further growth for 16–20 h. The cells were harvested, resuspended in NEBExpress *E. coli* lysis reagent (catalogue number P8116S, New England BioLabs) and sonicated gently using a Diagenode Bioruptor (UCD-300, Diagenode). The recombinant proteins including His–SUMO, His–SUMO–GRP20, His–SUMO–GRP20ΔRBD, His–SUMO–GRP20 (RBDm) and His–SUMO–GRP20ΔHDR were purified with Ni-NTA magnetic beads (1:1 mixture of two kinds of beads: catalogue number S1423S, New England BioLabs, and catalogue number 786-910, G-Biosciences) and ÄKTA Pure 150L FPLC and Frac-950 Fraction Collector (GE Healthcare) with a HiLoad 16/600 Superdex 200-pg column (GE Healthcare). Protein concentration was quantified using a Pierce BCA protein assay kit (catalogue number 23225, ThermoFisher).

### In vivo and in vitro interaction assay

For the BiFC experiment, full-length CDSs of *GRP20* and *Prp18* were cloned into the pXY104 (YFPn) and the pXY106 (YFPc) vectors, respectively. Then, the constructs were co-transformed into *Agrobacterium* cells (GV3101) for subsequent infiltration into *N. benthamiana* leaves as referenced^68^. The transformed leaves were analysed using LSM880 confocal microscopy (Zeiss, Germany). The co-transformations of pXY104–GRP20 and pXY106, pXY104 and pXY106–Prp18, and pXY104 and pXY106 were used as the negative controls. The co-infiltration of pXY104–MMD1 and pXY106–JMJ16 was used for a positive control. His pull-down assay or GST pull-down was performed as previously described^69^. The recombinant full-length GRP20 (residue 1 to 153), truncated GRP20ΔHDR (residue 1 to 118), HDR (residue 119 to 153), truncated GRP20Δ143–153 (residue 1 to 142) and mutated GRP20 (HDRm) proteins, fused with an N-terminal 6× histidine plus SUMO tag (His–SUMO), were purified from *E*. *coli* using Ni-NTA magnetic beads (catalogue number S1423S, New England BioLabs). The recombinant full-length Prp18 protein fused with an N-terminal GST tag (GST) was expressed in *E. coli* and purified using the Pierce glutathione purification beads (catalogue number 78601, Thermo Scientific). His–SUMO and GST proteins were also purified for negative controls. The concentration of purified proteins was determined by A280. The pull-down proteins were incubated with GST magnetic beads or Ni-NTA magnetic beads and detected using western blot with anti-His (mAb, 1:3,000 dilution, catalogue number MA1-21315, Invitrogen) and anti-GST (mAb, 1:5,000 dilution, catalogue number AE001, ABclonal) antibodies. Co-immunoprecipitation was conducted as described^70^; the CDS of *Prp18* was cloned into pCAMBIA1306 with the 35S promoter and transformed into the transgenic plants *Pro35S::GRP20-YFP* and *Pro35S::YFP*. The IP was incubated with protein G magnetic beads (catalogue number S1430S, New England BioLabs) and anti-GFP antibody (rabbit Ab, pAb, catalogue number AE011, ABclonal, 1:100), and the proteins were eluted using 5× SDS-PAGE loading buffer. The input and IP (immunoprecipitation) samples were detected using western blot with anti-GFP (pAb, catalogue number AE001, ABclonal, 1:1,000) and anti-FLAG (mAb, catalogue number AE005, ABclonal, 1:1,000) antibodies. For co-localization, full-length CDSs of *GRP20* and *Prp18* were cloned into the pGWB441 (YFP-tag) and pH7RWG2 (RFP-tag) vectors, respectively. The constructs were co-transformed into *Agrobacterium* cells (GV3101) for subsequent infiltration into *N. benthamiana*, and the co-localization was analysed using LSM880 confocal microscopy (Zeiss) by YFP and RFP channels.

### Plant protein extraction and western blot

Protein extraction and western blot analysis were performed as described previously^69^. Total protein and nuclear protein were extracted using protein extraction buffer (20 mM Tris–HCl pH 8.0, 300 mM NaCl, 1 mM EDTA, 10% glycerol, 1 mM PMSF and 1× protease inhibitor cocktail (catalogue number 11836170001, Roche)) from flower buds. The nuclei were passed through a 40 μm cell strainer (catalogue number 76327-098, VWR) and centrifuged at 3,500 rpm at 4 °C for 30 min. Antibodies (GST tag (mAb): catalogue number AE001, ABclonal, 1:1,000; His tag (mAb): catalogue number MA1-21315, Invitrogen, 1:1,000; GFP tag (pAb): catalogue number AE011, ABclonal, 1:1,000) were used in western blotting. Goat anti-rabbit secondary antibodies (catalogue number 31460, Invitrogen, 1:2,000) or goat anti-mouse secondary antibodies (catalogue number 62-6520, Invitrogen, 1:2,000) were used against the primary antibodies. Signals were visualized with a ChemiDoc image system (Bio-Rad). Anti-β-tubulin (pAb, catalogue number AC008, ABclonal, 1:1,000 dilution) and anti-histone 3 (pAb, catalogue number AS10710, Agrisera, 1:2,000 dilution) antibodies were used as the loading controls.

### Constructs for complementation and GRP20 mutant domains

The *GRP20* CDS was cloned into *Pro35S::pGWB441* (eYFP tag, enhanced YFP) by Gateway BP Clonase II Enzyme mix (catalogue number 11789100, ThermoFisher) and Gateway LR Clonase II Enzyme mix (catalogue number 11791020, ThermoFisher). The *GRP20* CDS was also cloned into *Pro*_*35S*_*::pCAMBIA1306* (FLAG tag) by KpnI/BamHI. The 35S promoter in *Pro*_*35S*_*::GRP20-pGWB441* was replaced by the *GRP20* promoter by digestion with MfeI/XbaI and ligation. The 35S promoter in *Pro*_*35S*_*::GRP20-pCAMBIA1306* was replaced by the *GRP20* promoter by digestion with EcoRI/KpnI and ligation. The 35S-driven GRP20–eYFP, 35S-driven GRP20–FLAG, GRP20-driven GRP20–eYFP and GRP20-driven GRP20–FLAG were transformed into WT and *grp20-1*, respectively. The background for transgenic plants was *grp20-1* for most experiments in this study and is referred to as *grp20*, unless otherwise indicated. The expression of GRP20-driven GRP20–eYFP was confirmed by western blotting with anti-GFP antibody (catalogue number AE011, ABclonal, 1:1,000 dilution) and anti-FLAG antibody (catalogue number AE005, ABclonal, 1:1,000 dilution).

Mutagenesis was conducted using a Q5 Site-Directed Mutagenesis Kit (catalogue number E0552S, New England BioLabs). The primers for mutagenesis were designed based on NEBaseChanger and are listed in Supplementary Table 7. The mutant GRP20 plasmids were transformed into *Agrobacterium* GV3101, including GRP20-driven GRP20 (RBDm)–eYFP, GRP20-driven GRP20ΔHDR–eYFP, 35S-driven GRP20ΔHDR–eYFP, 35S-driven HDR–eYFP, 35S-driven GRP20 (RBDm)–eYFP and GRP20-driven GRP20 (HDRm)–eYFP (m, mutation). The mutant constructs were transformed into WT and *grp20-1*. The expression of mutant GRP20 was confirmed by western blotting. At least five individual transgenic lines were analysed for each construct.

The normal CDSs of floral regulatory genes (*AP1*, *AP2*, *AP3*, *PI*, *SEP3*, *SEP4* and *AS2*) were cloned into *Pro35S::pGWB441* (eYFP tag, enhanced YFP) by Gateway reactions or *Pro35S::N1-cYFP* vector by enzymatic digestion and ligation. Then, the 35S promoter in these constructs was replaced by native promoters of floral regulatory genes by enzymatic digestion and ligation. The deletion of micro exons that were affected by GRP20 in floral homeotic genes was constructed through the Q5 Site-Directed Mutagenesis Kit. The alternative 5′ site fragments of *AS2* were amplified from the *grp20-1* flower cDNA library and then inserted into Pro35S::N1-cYFP. The 35S promoter of this construct was then replaced by the native promoter of *AS2*. For each transgenic line, at least two individual lines were obtained.

The cDNA of GRP20 homologues (*B. rapa*, *G. max*, *O. sativa* and *A. trichopoda*) was amplified from the cDNA library of *B. rapa* or genomic DNA of *G. max*, *O. sativa* and *A. trichopoda*, and then inserted into *Pro*_*AtGRP20*_*::N1-cYFP* vectors. The complementation constructs of *Pro*_*AtGRP20*_*::BrGRP20*, *Pro*_*AtGRP20*_*::GmGRP20*, *Pro*_*AtGRP20*_*::OsGRP20* and *Pro*_*AtGRP20*_*::AmGRP20* were transformed into GV3101 and induced into *Arabidopsis* with *grp20-1* background. For each transgenic plant, at least two individual lines were obtained.

The similar expression levels of GRP20, GRP20 (RBDm) and GRP20ΔHDR proteins in transgenic plants (Fig. 6c and Extended Data Fig. 9b) and in *E. coli* (Supplementary Fig. 7a) suggest that normal and mutant GRP20 proteins are translated with similar stability. In addition, the predicted protein structures were not affected by the amino acid changes (Supplementary Fig. 7b), using structure prediction^71^ and simulation (https://swissmodel.expasy.org/). These results suggest that the folding of the mutant protein was probably not drastically affected by the amino acid substitutions.

### Protein condensates, fluorescent photo bleaching and the corresponding constructs

The protein condensates were observed as described^51^ in sepals and petals of *Arabidopsis* transgenic plants (*Pro*_*GRP20*_*::GRP20–eYFP; grp20*) and also in epidermal cells of tobacco leaves with one of the following constructs: *Pro*_*35S*_*::eYFP*, *Pro*_*35S*_*::GRP20–eYFP,* *Pro*_*35S*_*::GRP20ΔHDR (residue 1 to 118)–eYFP*, *Pro*_*35S*_*::HDR [GRP20 (residue 119 to 153)]–eYFP*, *Pro*_*35S*_*::GRP20 (RBDm)–eYFP*, *Pro*_*35S*_*::GRP20Δ143–153 (residue 1 to 142)–eYFP* and *Pro*_*35S*_*::GRP20 (HDRm)–eYFP*. DAPI (0.05 mg ml^−1^) was used to stain the nuclei of *Arabidopsis* and tobacco cells. Fluorescent bleaching was conducted as described previously^72^ and in the Zeiss manual. The full-length GRP20 (*Pro*_*35S*_*::GRP20 (residue 1 to 153)–eYFP*), GRP20 without HDR (*Pro*_*35S*_*::GRP20 (residue 1 to 118)–eYFP*), HDR alone (*Pro*_*35S*_*::GRP20 (residue 119 to 153)–eYFP*), *Pro*_*35S*_*::GRP20Δ143-153 (residue 1 to 142)–eYFP* and *Pro*_*35S*_*::GRP20 (HDRm)–eYFP)* were transiently transformed into the bottom surface of *N. benthamiana* leaves. Protein condensates were bleached, and the intensity of fluorescence was calculated using confocal microscopy and an associated computational tool (Zeiss) following the manufacturer’s instructions.

### Protein domain alignment, conservation analyses and orthogroup searching

The evolutional relationship of representative angiosperms was derived from published phylogeny^73^. The relationship among ABCE family genes was obtained from previous studies^74^. The protein sequences of *Arabidopsis* GRP20 and its homologues from other species were downloaded from Phytozome (https://phytozome-next.jgi.doe.gov/) and Uniprot (https://www.uniprot.org/), with a threshold of similarity ≥60%, to focus on the close homologues. The protein alignment of GRP20 homologues was conducted using Muscle in MEGA 7 (ref. ^75^). The protein sequences encoded by micro exons of ABCE genes were obtained from Uniprot, and the alignment was conducted using Muscle in MEGA 7. The illustrations of gene structures of ABCE family genes were generated using Gene Structure Display Server (version 2.0)^76^ and TBtools^77^. The GFF3 file was downloaded from TAIR (https://www.arabidopsis.org/). The CDSs and genomic sequences were downloaded from Phytozome. The orthogroup searching and copy number identification for 13 angiosperms (*Arabidopsis*, papaya, grape, poplar, tomato, lettuce, carrot, rice, sorghum, quinoa, pineapple, water lily and *Amborella*) and one gymnosperm (Ginkgo) were performed as referenced^78^. The whole *Arabidopsis* genes were used as queries to search for 1 gymnosperm and 13 high-quality angiosperm genomes using BLASTP with a strict *E*-value threshold of less than 1 × 10^−5^ and a minimal amino acid sequence identity of 30%.

### Statistics and reproducibility

Three experiments were repeated independently with similar results for micrographs in Figs. 6c, 7d and 8a,c; Extended Data Figs. 6h, 8g,h,j, 9c and 10c; and Supplementary Figs. 4b, 5b,d and 7a. For flower images or cell images in Fig. 8b,d; Extended Data Figs. 6b,e–g, 9f,g and 10f; and Supplementary Fig. 5c, three observations were repeated independently with similar results in 20 individual flowers or 25 individual cells. The statistical test used for GO annotations in Supplementary Table 3 and for differential expressed genes in Supplementary Table 6 is the Mann–Whitney *U* test with 95% confidence intervals and FDR correction, and Fisher’s test with 95% confidence intervals, respectively.

### Reporting summary

Further information on research design is available in the Nature Portfolio Reporting Summary linked to this article.

### Supplementary information

Supplementary Information Supplementary Figs. 1–7 and uncropped scans of blots and gels in the Supplementary Figs. 4b, 5b,d and 7a.

Reporting Summary

Supplementary Tables Supplementary Tables 1–7. The title and descriptive captions for each table have been included in the file itself.

Supplementary Data Statistical source data for Supplementary Figs. 2c,d, 3c,f, 4a, 5g and 6b,e–h.

### Source data

Source Data Fig. 2 Statistical source data for Fig. 2e,f.

Source Data Fig. 3 Statistical source data for Fig. 3a,j.

Source Data Fig. 6 Unprocessed western blots and gels for Fig. 6c.

Source Data Fig. 7 Unprocessed western blots and gels for Fig. 7d.

Source Data Fig. 8 Unprocessed western blots and gels for Fig. 8a,c.

Source Data Extended Data Fig. 1 Statistical source data for Extended Data Fig. 1i.

Source Data Extended Data Fig. 4 Statistical source data for Extended Data Fig. 4e.

Source Data Extended Data Fig. 6 Statistical source data for Extended Data Fig. 6d.

Source Data Extended Data Fig. 6 Unprocessed western blots and gels for Extended Data Fig. 6h.

Source Data Extended Data Fig. 8 Unprocessed western blots and gels for Extended Data Fig. 8g,h,j.

Source Data Extended Data Fig. 9 Unprocessed western blots and gels for Extended Data Fig. 9c.

Source Data Extended Data Fig. 10 Statistical source data for Extended Data Fig. 10b,d.

Source Data Extended Data Fig. 10 Unprocessed western blots and gels for Extended Data Fig. 10c.

## Data Availability

All data are available in the main text or Supplementary Information. Raw data of RNA-seq of WT and *grp20* floral and leaf transcriptomes have been deposited in the SRA database of NCBI with accession number PRJNA851744. The gene and protein information of *Arabidopsis* and other species were obtained from TAIR (https://www.arabidopsis.org/), UniProt (https://www.uniprot.org/) and Phytozome v13 (https://phytozome-next.jgi.doe.gov/). The prediction protein structures were obtained from AlphaFold Protein Structure Database (https://alphafold.ebi.ac.uk/). Source data are provided with this paper.
