# Supplementary material for: Regulation of micro- and small-exon retention and other splicing processes by GRP20 for flower development
Source: Nat Plants. 2024 Jan 9;10(1):66–85. doi: 10.1038/s41477-023-01605-8 (PMC10808074; doi:10.1038/s41477-023-01605-8)

nature plants

Article

https://doi.org/10.1038/s41477-023-01605-8

Regulation of micro- and small-exon retention and other splicing processes by GRP20 for flower development

In the format provided by the authors and unedited

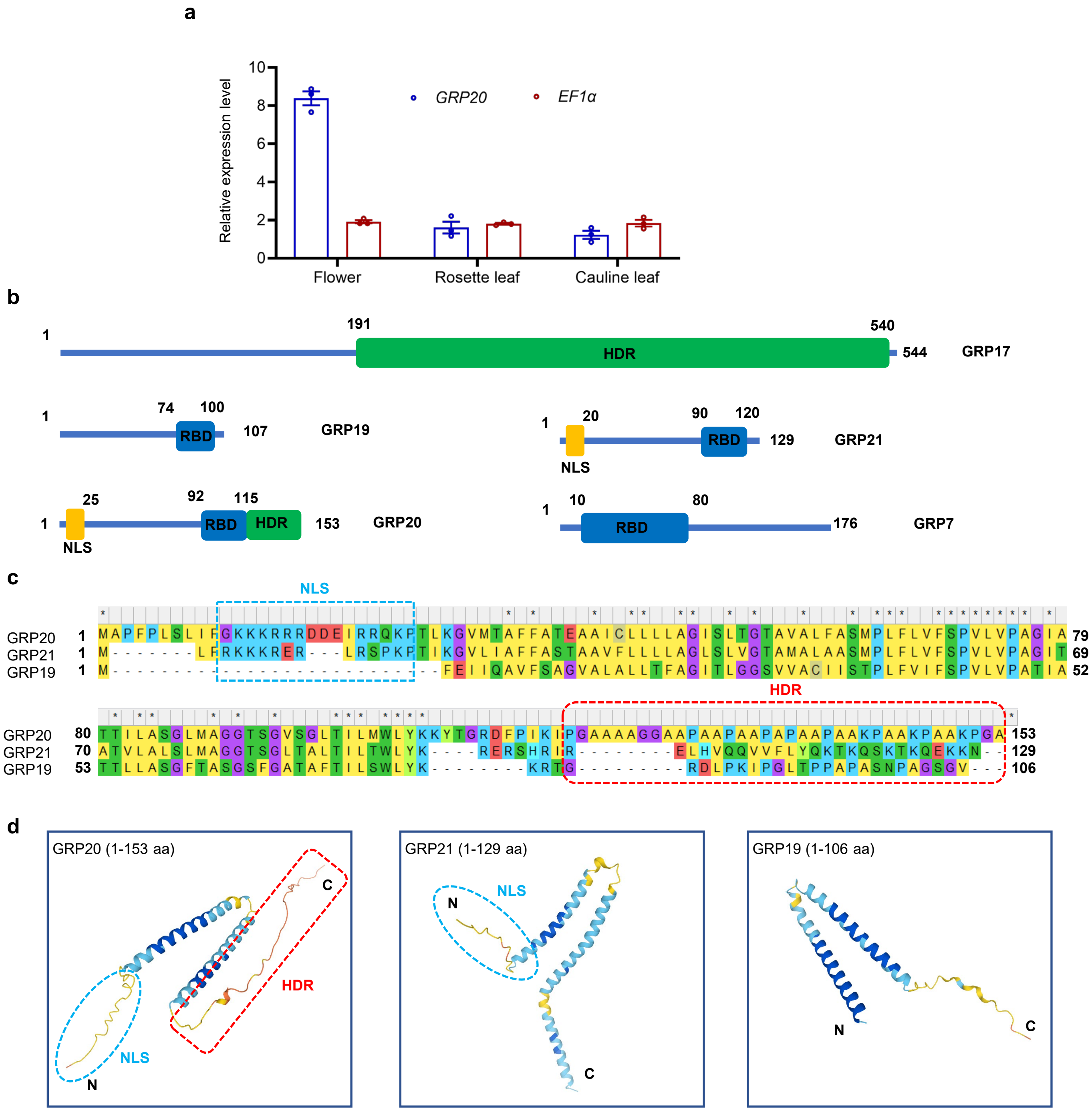

Supplementary Fig. 1. The protein domain, alignment and predicated structures of glycine-rich proteins in the adjacent loci and GRP7.
a, The relative expression levels of *GRP20* and *EF1α* in WT flower, rosette leaf and cauline leaf. Data are presented as mean ± SEM with three independent replicates. **b,** The illustration of protein domain of GRP 17, GRP19, GRP20 and GRP21 in the adjacent genomic loci and GRP7. NLS: nuclear localization signal; RBD: potential RNA binding domain; HDR: highly disorder region. **c,** The protein alignment of GRP19, GRP20 and GRP21 showing that the HDR is absent in GRP21, and NLS and HDR are both absent in GRP19. **d,** The prediction of protein structures of GRP19, GRP20 and GRP21 by AlphaFold2. NLS and HDR are marked by blue and red frame, respectively, in **c** and **d**.

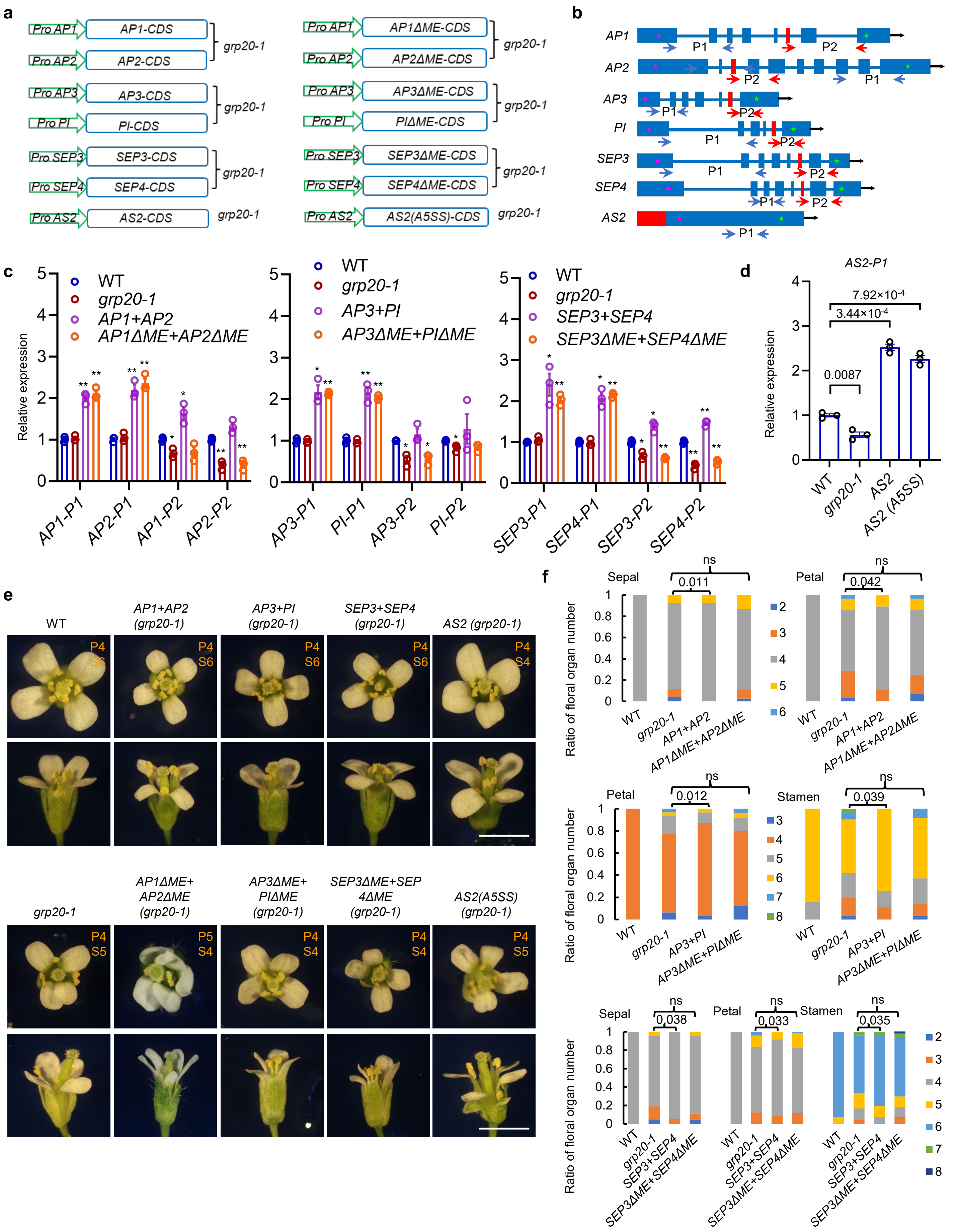
a
Pro AP1
AP1-CDS
Pro AP2
AP2-CDS
grp20-1
Pro AP3
AP3-CDS
Pro PI
PI-CDS
grp20-1
Pro SEP3
SEP3-CDS
Pro SEP4
SEP4-CDS
grp20-1
Pro AS2
AS2-CDS
grp20-1
Pro AP1
AP1ΔME-CDS
Pro AP2
AP2ΔME-CDS
grp20-1
Pro AP3
AP3ΔME-CDS
Pro PI
PIΔME-CDS
grp20-1
Pro SEP3
SEP3ΔME-CDS
Pro SEP4
SEP4ΔME-CDS
grp20-1
Pro AS2
AS2(A5SS)-CDS
grp20-1
b
AP1
P1
P2
AP2
P2
P1
AP3
P1
P2
PI
P1
P2
SEP3
P1
P2
SEP4
P1
P2
AS2
P1
c
WT
grp20-1
AP1+AP2
AP1ΔME+AP2ΔME
Relative expression
AP1-P1
AP2-P1
AP1-P2
AP2-P2
WT
grp20-1
AP3+PI
AP3ΔME+PIΔME
AP3-P1
PI-P1
AP3-P2
PI-P2
WT
grp20-1
SEP3+SEP4
SEP3ΔME+SEP4ΔME
SEP3-P1
SEP4-P1
SEP3-P2
SEP4-P2
d
AS2-P1
7.92×10-4
3.44×10-4
0.0087
Relative expression
WT
grp20-1
AS2
AS2 (A5SS)
e
WT
AP1+AP2 (grp20-1)
AP3+PI (grp20-1)
SEP3+SEP4 (grp20-1)
AS2 (grp20-1)
P4 S6
P4 S6
P4 S6
P4 S6
P4 S4
grp20-1
AP1ΔME+ AP2ΔME (grp20-1)
AP3ΔME+ PIΔME (grp20-1)
SEP3ΔME+SEP 4ΔME (grp20-1)
AS2(A5SS) (grp20-1)
P4 S5
P5 S4
P4 S4
P4 S4
P4 S5
f
Sepal
ns
0.011
Petal
ns
0.042
Ratio of floral organ number
WT
grp20-1
AP1+AP2
AP1ΔME+AP2ΔME
2
3
4
5
6
Petal
ns
0.012
Stamen
ns
0.039
WT
grp20-1
AP3+PI
AP3ΔME+PIΔME
3
4
5
6
7
8
Sepal
ns
0.038
Petal
ns
0.033
Stamen
ns
0.035
WT
grp20-1
SEP3+SEP4
SEP3ΔME+SEP4ΔME
2
3
4
5
6
7
8

Supplementary Fig. 2. The investigation of phenotypes and RNA splicing in transgenic plants with wild-type and micro exon skipped ABE genes, and wild-type and alternative 5' spliced *AS2* in *grp20* background.

a, The illustration of constructions of wild-type and micro exon skipped ABE genes, and wild-type and alternative 5' spliced LOB domain gene *AS2* used in the complementation experiments. **b,** Illustrations of gene structures with positions of primers used for investigation in *AP1*, *AP2*, *AP3*, *PI*, *SEP3*, *SEP4* and a transcript with A5SS in *AS2*; the blue boxes indicate exons and thin lines represent introns. The red boxes indicate skipped micro exons in ABE genes. A red box in *AS2* represents an alternative extra exonic region (A5SS) in *AS2*. The red and blue lines with arrows under the gene structures indicate the primers used for expression detection. P1 is used to detect the wild-type expression of transgene in transgenic plants and P2 indicates the relative expression of inclusive micro exons that are skipped in ABE genes or alternative 5' spliced transcripts of *AS2* in *grp20-1*. **c,** The expression of selected regions of ABE genes in wild-type transgenic lines and micro exon skipped transgenic lines by RT-qPCR. For *AP1* and *AP2*, from left to right, *p*-value between WT and each genotype is: 0.0018, 0.0014; 0.0066, 0.0039; 0.017, 0.039; 0.0037, 0.0066. For *AP3* and *PI*, from left to right, *p*-value is: 0.016, 3.63×10^{-5}; 0.0077, 6.23×10^{-5}; 0.029, 0.029; 0.052. For *SEP3* and *SEP4*, from left to right, *p*-value is: 0.035, 0.0052; 0.012, 5.11×10^{-5}; 0.01, 0.026, 5.1×10^{-4}; 0.0011, 0.0018, 4.05×10^{-4}. **d,** The expression of selected regions of *AS2* in wild-type transgenic lines and alternative 5' spliced transgenic lines by RT-qPCR. **e,** The floral phenotypes of WT, *grp20* and transgenic lines of ABE genes and *AS2*. Bar = 1 mm. The yellow letter and number at top right of each panel indicate corresponding organ and number in the flower. P: Petal; S: Stamen. **f,** The floral organ quantification in WT, *grp20* and transgenic lines shown in **e** and detailed flower counts were shown in Supplementary Table 5. Data are presented as fraction (ratio) of floral organ number in total organ count. For examples, each flower in WT has 4 sepals, therefore, the fraction of 4 in WT is 1 (100%, 19/19). However, among 26 flowers of *grp20-1*, 1, 2, 21 and 2 flowers have 2, 3, 4, 5 sepals, respectively. Therefore, the fractions of 2, 3, 4, 5 in *grp20-1* is 0.038 (1/26), 0.077 (2/26), 0.808 (21/26) and 0.077 (2/26). Data are presented as mean ± SEM with three independent replicates in **c** and **d**. * $p<0.05$, ** $p<0.01$, two-sided Student's *t* test. ns indicates not significant.

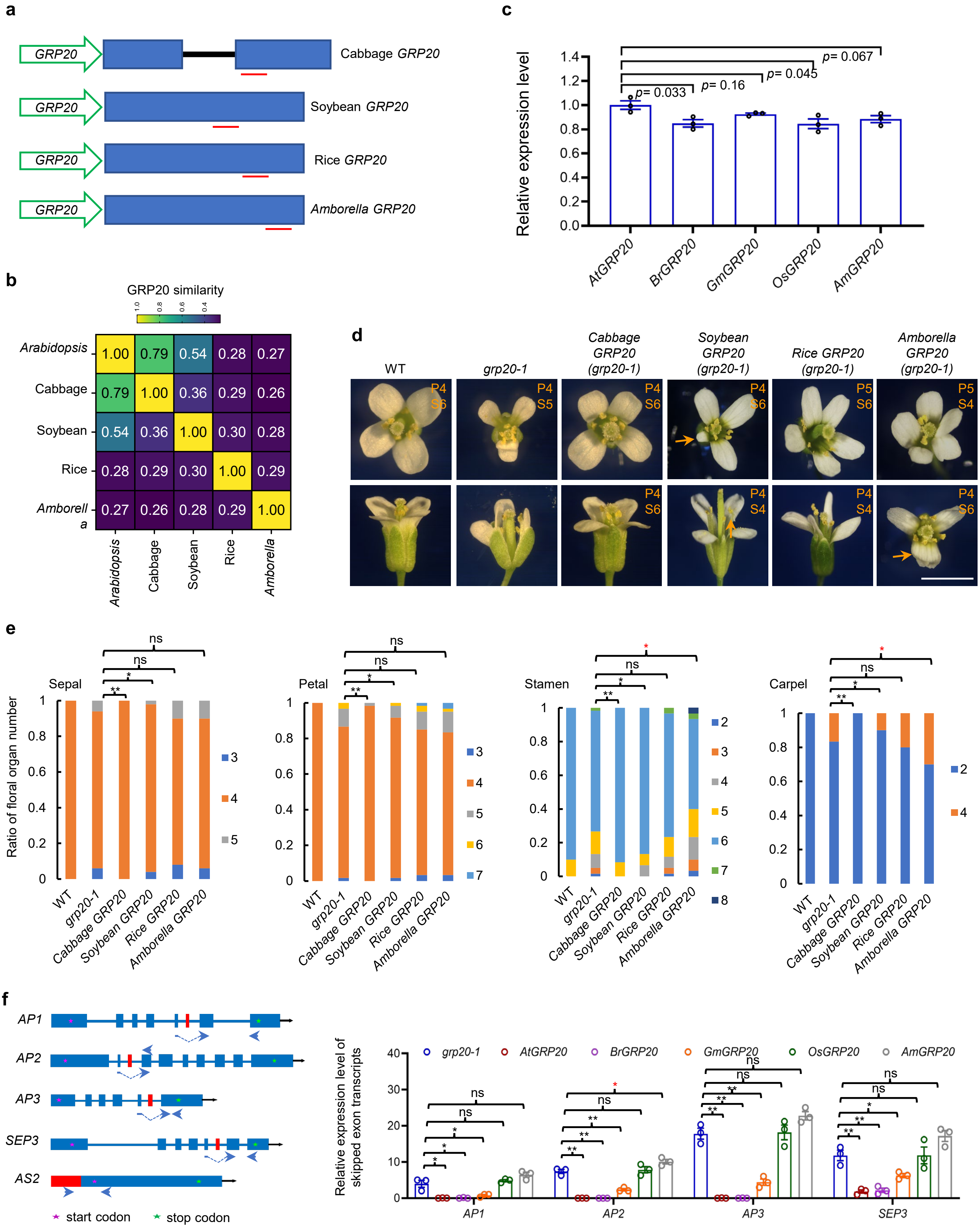

a
GRP20
Cabbage GRP20
Soybean GRP20
Rice GRP20
Amborella GRP20
b
GRP20 similarity
Arabidopsis
Cabbage
Soybean
Rice
Amborella
1.00 0.79 0.54 0.28 0.27
0.79 1.00 0.36 0.29 0.26
0.54 0.36 1.00 0.30 0.28
0.28 0.29 0.30 1.00 0.29
0.27 0.26 0.28 0.29 1.00
c
Relative expression level
p= 0.033
p= 0.16
p= 0.045
p= 0.067
AtGRP20
BrGRP20
GmGRP20
OsGRP20
AmGRP20
d
WT
grp20-1
Cabbage GRP20 (grp20-1)
Soybean GRP20 (grp20-1)
Rice GRP20 (grp20-1)
Amborella GRP20 (grp20-1)
e
Sepal
Petal
Stamen
Carpel
Ratio of floral organ number
WT
grp20-1
Cabbage GRP20
Soybean GRP20
Rice GRP20
Amborella GRP20
ns
f
AP1
AP2
AP3
SEP3
AS2
start codon
stop codon
Relative expression level of skipped exon transcripts
grp20-1
AtGRP20
BrGRP20
GmGRP20
OsGRP20
AmGRP20

Supplementary Fig. 3. Analyses of functions of potential *GRP20* homologues.

a, The illustration of constructions of *GRP20* homologues from *Brassica rapa* (cabbage, *BrGRP20*), *Glycine max* (soybean, *GmGRP20*), *Oryza sativa* (rice, *OsGRP20*) and *Amborella trichopoda* (*Amborella*, *AmGRP20*) driven by *AtGRP20* promoter used in the complementation experiments. Blue box and black line indicates exon and intron, respectively. *BrGRP20* has an intron, however, *GmGRP20*, *OsGRP20* and *AmGRP20*, each only has one exon. The red lines with arrows indicate the primers used for expression detection. **b,** The protein similarity among GRP20 homologues. **c,** The relative expression of *GRP20* homologues in WT and *atgrp20* mutant background. **d,** The floral phenotypes of WT, *grp20* and complementation lines. Bar = 1 mm. The yellow letter and number at top right of each panel indicate corresponding organ and number in the flower. P: Petal; S: Stamen. Yellow arrows indicate small petals and an abnormal stamen with two anthers in the head in *GmGRP20* transgenic lines, and abnormal petals in *AmGRP20* transgenic plants. **e,** The floral organ quantification in WT, *grp20* and complementation lines and detailed counts were shown in Supplementary Table 5. Data are presented as fraction (ratio) of floral organ number in total organ count. For examples, 50 flowers of WT have 4 sepals, therefore, the fraction of 4 in WT is 1 (100%, 50/50). However, among 50 flowers of *grp20-1*, 3, 44 and 3 flowers have 3, 4 and 5 sepals, respectively. Therefore, the fractions of 3, 4 and 5 in *grp20-1* is 0.06 (3/50), 0.88 (44/50) and 0.06 (3/50). *p*-value is shown from left to right for comparison between *grp20* and cabbage *GRP20*; *Soybean GRP20*; *Rice GRP20, Amborella GRP20.* For sepal, *p*-value is 0.005, 0.037, 0.079, 0.059; for petal, *p*-value is 0.0021, 0.020, 0.37, 0.54; for stamen, *p*-value is 0.0023, 0.038, 0.35, 0.036; for carpel, *p*-value is 0.0011, 0.013, 0.32, 0.032. **f,** Illustrations of gene structures with positions of primers used for investigation of micro exon skipping in *AP1*, *AP3*, *SEP3*, *AP2* and a transcript with A5SS in *AS2* in Supplementary Figs. 3f, 5g, 6e-h; the blue boxes indicate exons and thin lines represent introns. The red boxes indicate skipped micro exons in *AP1*, *AP3*, *SEP3* and *AP2*. A red box in *AS2* represents an alternative extra exonic region (A5SS) in *AS2*. The relative expression of skipped micro exon transcripts of *AP1*, *AP2*, *AP3* and *SEP3* in WT, *grp20* and complementation lines. *p*-value is shown from left to right for comparison between *grp20* and *AtGRP20*; *grp20* and *BrGRP20*; *grp20* and *GmGRP20*; *grp20* and *OsGRP20*; *grp20* and *AmGRP20*; For *AP1*, *p*-value is 0.016, 0.016, 0.034, 0.47, 0.12; for *AP2*, *p*-value is 0.0056, 1.8×10^{-4}, 0.0015, 0.68, 0.033; for *AP3*, *p*-value is 0.0071, 3×10^{-4}, 0.0016, 0.90, 0.06; for *SEP3*, *p*-value is 0.0029, 0.0037, 0.024, 1.0, 0.063. Data were presented as mean ± SEM with three independent replicates in **c** and **f**. Red star indicates the significantly more severe phenotypes or changes compared to WT. * $p<0.05$, ** $p<0.01$, two-sided Student's *t* test. ns indicates not significant.

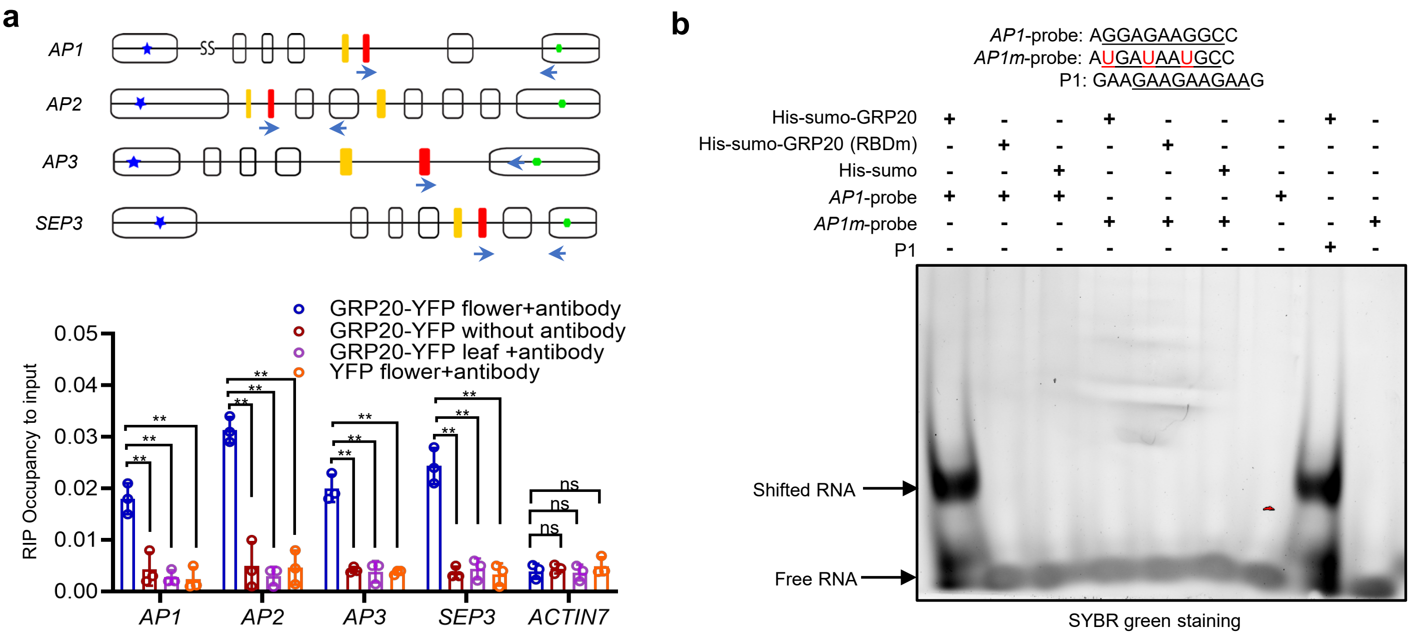

Supplementary Fig. 4. The in vivo and in vitro binding of GRP20 to micro exons of ABCE genes.
a, The RNA immunoprecipitation (RIP) between GRP20 and ABE genes. The binding to micro exon regions of *AP1*, *AP2*, *AP3* and *SEP3* are tested by RIP-qPCR using the primers indicated under the gene structure by blue arrows. The affected micro exons are highlighted by red in gene structure diagrams on the right. The other micro exons are shown in orange, whereas other exons are shown as open boxes. Blue asterisks and green boxes indicate star codon and stop codon, respectively. In RIP experiments, GRP20-YFP without antibody, GRP20-YFP leaf with antibody and 35S::YFP flowers with antibody were used as negative experimental controls. *ACTIN7* was a negative control for RNA-binding targets. From left to right, for *AP1*, *p*-value is 0.0058, 0.0054, 0.0026; for *AP2*, *p*-value is 0.0028, 1.79×10^{-4}, 5.3×10^{-4}; for *AP3*, *p*-value is 0.0071, 0.0014, 0.0073; for *SEP3*, *p*-value is 0.0054, 0.0026, 0.0021; for *ACTIN7*, *p*-value is 0.66, 0.86, 0.49. **b,** The EMSA test between GRP20 and native probe from *AP1*. The probes of *AP1*, *AP1m* (mutations of three G's to three U's) and Probe 1 are indicated above EMSA gel. His-sumo and His-sumo-GRP20 (RBDm) were used as protein negative controls. Probe 1 is used as a positive control. The mutations in the RBD were selected based on predicted RNA binding domain and residues shown in Extended Data Fig. 1. Data are presented as mean ± SEM with three independent replicates in **a**. * $p<0.05$, ** $p<0.01$, two-sided Student's *t* test. ns indicates not significant.

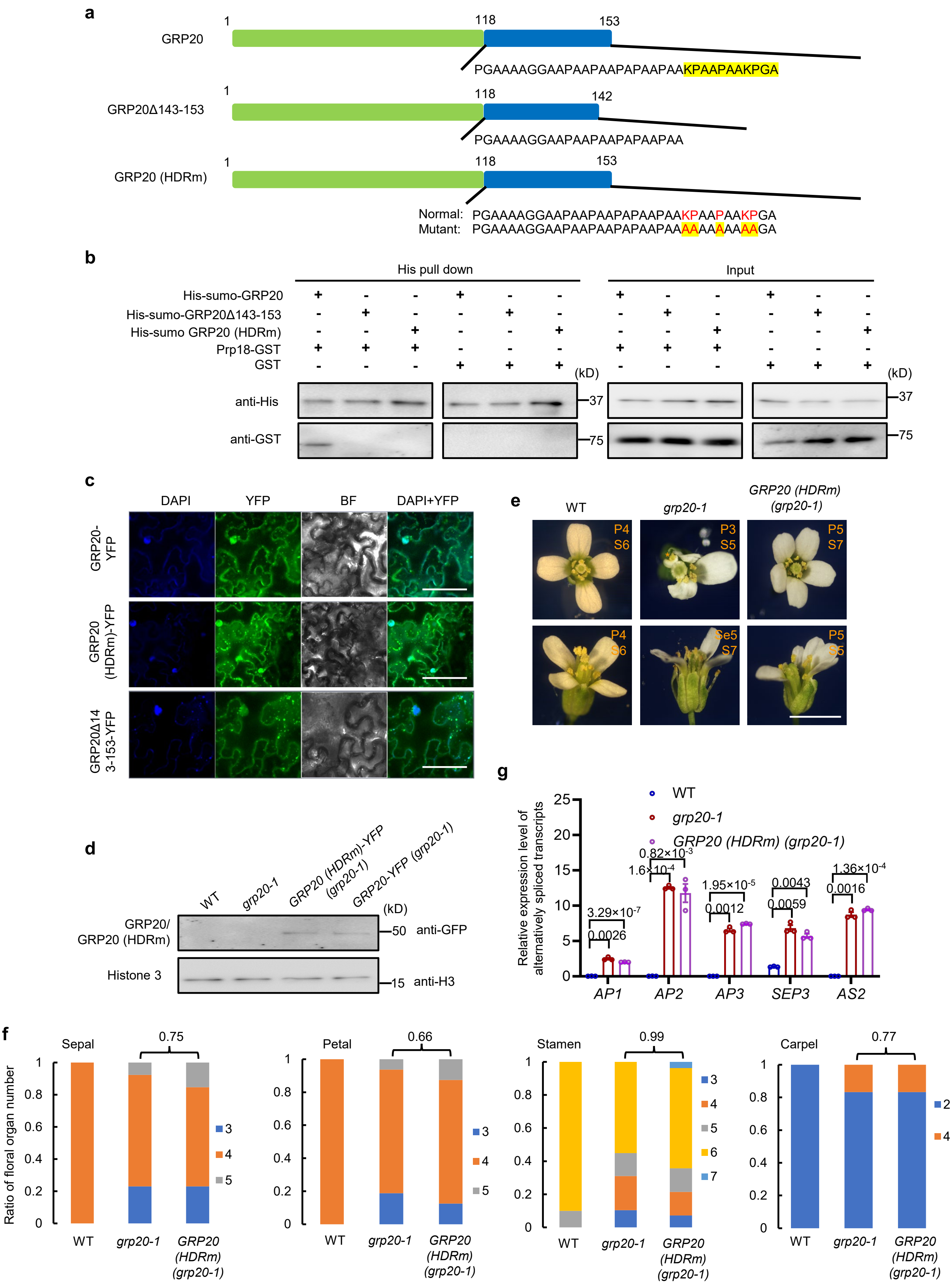
a
GRP20
1
118
153
PGAAAAGGAAPAAPAAPAPAAPAAKPAAPAAKPGA
GRP20Δ143-153
142
PGAAAAGGAAPAAPAAPAPAAPAA
GRP20 (HDRm)
Normal: PGAAAAGGAAPAAPAAPAPAAPAAKPAAPAAKPGA
Mutant: PGAAAAGGAAPAAPAAPAPAAPAAAAAAAAAAAGA
b
His pull down
Input
His-sumo-GRP20
His-sumo-GRP20Δ143-153
His-sumo GRP20 (HDRm)
Prp18-GST
GST
(kD)
anti-His
anti-GST
37
75
c
DAPI
YFP
BF
DAPI+YFP
GRP20-YFP
GRP20 (HDRm)-YFP
GRP20Δ143-153-YFP
d
WT
grp20-1
GRP20 (HDRm)-YFP (grp20-1)
GRP20-YFP (grp20-1)
(kD)
GRP20/ GRP20 (HDRm)
Histone 3
50
15
anti-GFP
anti-H3
e
WT
grp20-1
GRP20 (HDRm) (grp20-1)
P4 S6
P3 S5
P5 S7
P4 S6
Se5 S7
P5 S5
g
WT
grp20-1
GRP20 (HDRm) (grp20-1)
Relative expression level of alternatively spliced transcripts
3.29×10^-7
0.0026
0.82×10^-3
1.6×10^-4
1.95×10^-5
0.0012
0.0043
0.0059
1.36×10^-4
0.0016
AP1
AP2
AP3
SEP3
AS2
f
Ratio of floral organ number
Sepal
0.75
Petal
0.66
Stamen
0.99
Carpel
0.77
WT
grp20-1
GRP20 (HDRm) (grp20-1)

Supplementary Fig. 5. The important role of interaction between GRP20 and spliceosome in flower development and RNA splicing.
a, The illustration of C-terminal deletion GRP20 protein (GRP20Δ143-153) and mutated GRP20 protein (HDRm) in HDR regions. **b,** The pull-down assay between truncated/mutated GRP20 proteins and Prp18. GST was used as the negative control. **c,** The condensation formation of truncated/mutated GRP20 fusion with YFP proteins in tobacco leaves. Bar= 20 μm. **d,** The protein level in WT, *grp20* and complementation lines detected by Western blotting. The GRP20-YFP and GRP20 (HDRm)-YFP proteins in transgenic plants were detected by anti-GFP. WT and *grp20-1* plants were used as the negative control. Histone 3 detected by anti-H3 were used as the loading control. **e,** The floral phenotypes of WT, *grp20* and *GRP20 (HDRm)* complementation lines. Bar = 1 mm. The yellow letter and number at top right of each panel indicate corresponding organ and number in the flower. Se: Sepal; P: Petal; S: Stamen. The asterisk indicates the chimeric organ. **f,** The floral organ quantification in WT, *grp20* and *GRP20 (HDRm)* complementation lines and detailed counts were shown in Supplementary Table 5. Data are presented as fraction (ratio) of floral organ number in total organ count. For examples, 11 flowers of WT have 4 sepals, therefore, the fraction of 4 in WT is 1 (100%, 50/50). However, among 13 flowers of *grp20-1*, 3, 9 and 1 flowers have 3, 4 and 5 sepals, respectively. Therefore, the fractions of 3, 4 and 5 in *grp20-1* is 0.231 (3/13), 0.692 (9/13) and 0.077 (1/13). **g,** The relative expression level of alternatively spliced transcripts of *AP1*, *AP2*, *AP3*, *SEP3* (micro exon skipping) and *AS2* (alternative 5' site splicing) in WT, *grp20* and *GRP20 (HDRm)* complementation lines. The primers used for RT-qPCR were shown in the Supplementary Fig. 3f. Data are presented as mean ± SEM with three independent replicates in **g**. Two-sided Student's *t* test.

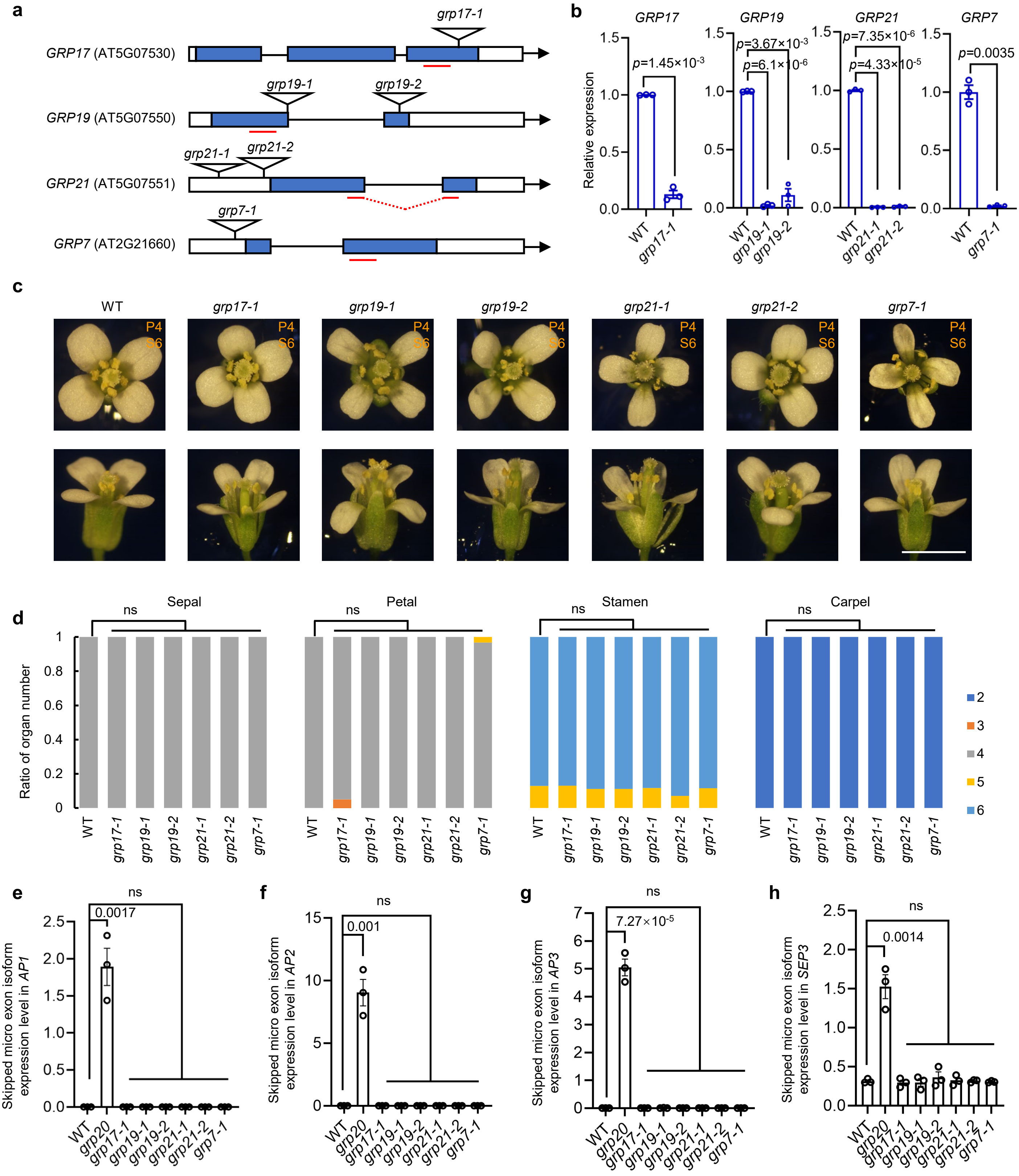

a
GRP17 (AT5G07530)
grp17-1
GRP19 (AT5G07550)
grp19-1
grp19-2
GRP21 (AT5G07551)
grp21-1
grp21-2
GRP7 (AT2G21660)
grp7-1
b
GRP17
GRP19
GRP21
GRP7
Relative expression
p=1.45×10-3
p=3.67×10-3
p=6.1×10-6
p=7.35×10-6
p=4.33×10-5
p=0.0035
WT
grp17-1
grp19-1
grp19-2
grp21-1
grp21-2
grp7-1
c
WT
grp17-1
grp19-1
grp19-2
grp21-1
grp21-2
grp7-1
P4
S6
d
Sepal
Petal
Stamen
Carpel
ns
Ratio of organ number
2
3
4
5
6
e
f
g
h
Skipped micro exon isoform expression level in AP1
Skipped micro exon isoform expression level in AP2
Skipped micro exon isoform expression level in AP3
Skipped micro exon isoform expression level in SEP3
0.0017
0.001
7.27×10-5
0.0014
ns
grp20

Supplementary Fig. 6. The phenotypic analyses of mutants of glycine-rich proteins.

a, The gene structures and T-DNA insertions in other glycine-rich proteins. Black line, blue box and open box indicates intron, exon and UTR region, respectively. The red lines below genes indicate the primers used for relative expression detection. **b,** The relative expression of glycine rich genes in flowers of WT and corresponding mutants. **c,** The flower phenotypes of glycine-rich mutants. Bar = 1 mm. The yellow letter and number at top right of each panel indicate corresponding organ and number in the flower. P: Petal; S: Stamen. The GRP mutants showed similar flowers to WT. **d,** The floral organ counts in glycine-rich mutants and detailed counts were shown in Supplementary Table 5. Data are presented as the fraction (ratio) of floral organ number in total organ count. For examples, 38 flowers of WT have 4 sepals, therefore, the fraction of 4 in WT is 1 (100%, 50/50). However, all 20 flowers of *grp17-1* have 4 sepals. Therefore, the fractions of 4 in *grp17-1* is also 1 (100%, 20/20). **e-h,** The relative expression of skipped micro exon transcripts of *AP1* (e), *AP2* (f), *AP3* (g) and *SEP3* (h) in glycine-rich mutants. The primers used for RT-qPCR were shown in the Supplementary Fig. 3f. Data are presented as mean ± SEM with three independent replicates in **b** and **e-h**. Two-sided Student's *t* test. ns indicates not significant or not detectable.

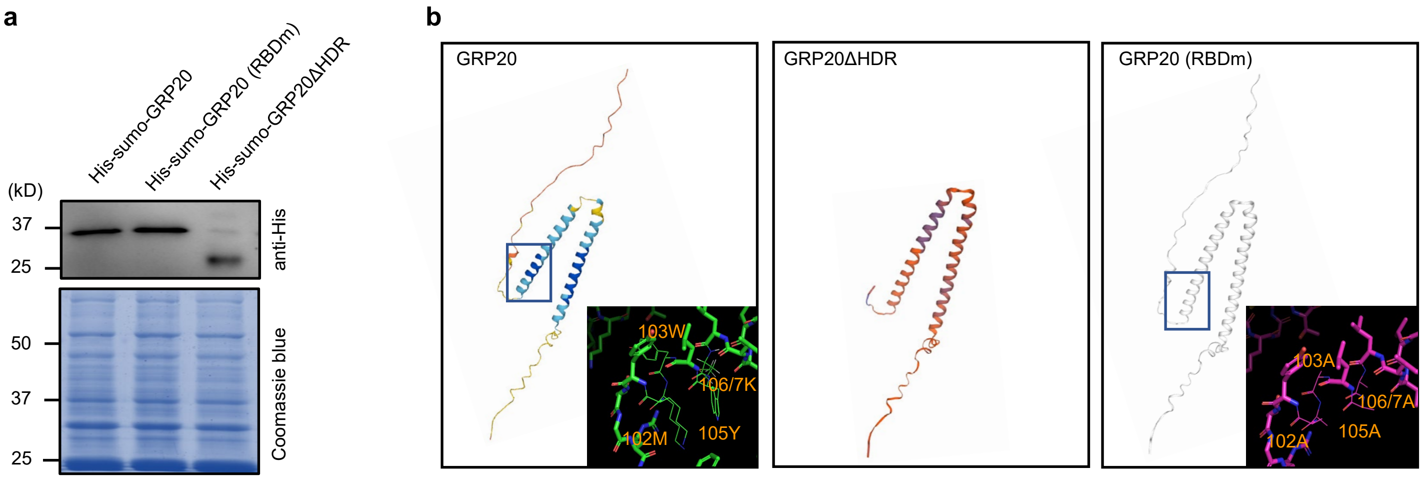

Supplementary Fig. 7. The mutations of GRP20 likely do not affect protein translation and folding.

a, The protein translation detected by western blotting and expressed by *E. coli*. The deletion of HDR and mutation of RBD likely do not affect protein translation. **b,** The predicted structure of GRP20 by AlphaFold2 and the structure simulation of GRP20 mutants by Swiss-model. The right-down panels show the mutation loci of RBD marked by blue frames with detail amino acid changes. The structure prediction indicates that the mutation and deletion likely do not affect protein folding.

Supplementary Fig. 4b

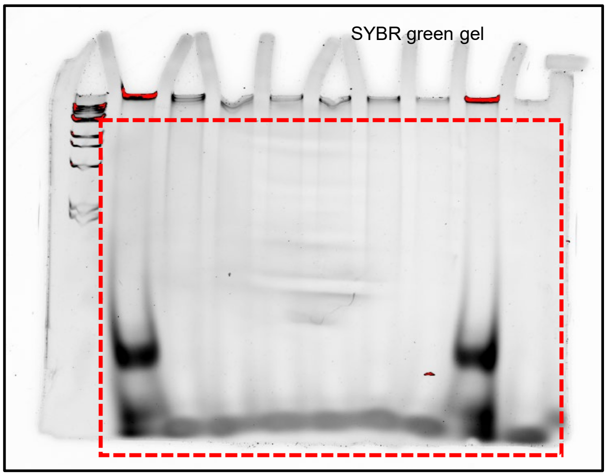

Supplementary Fig. 5b

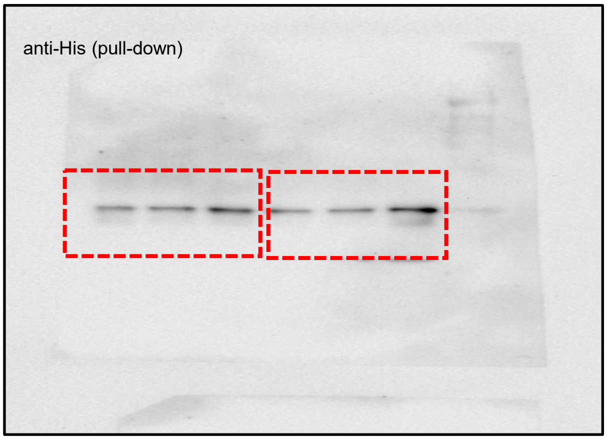

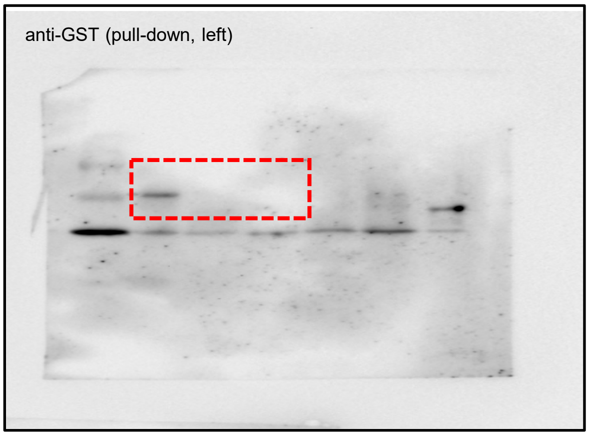

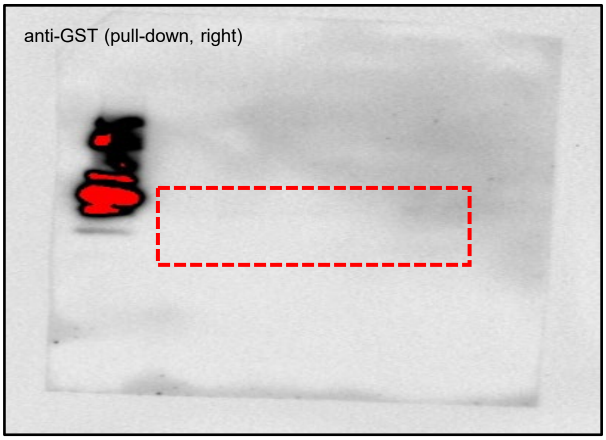

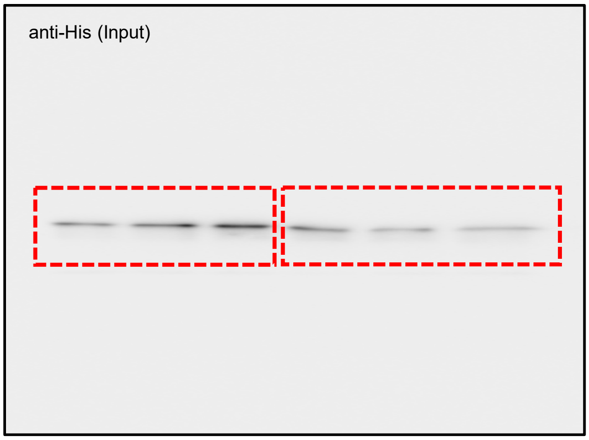

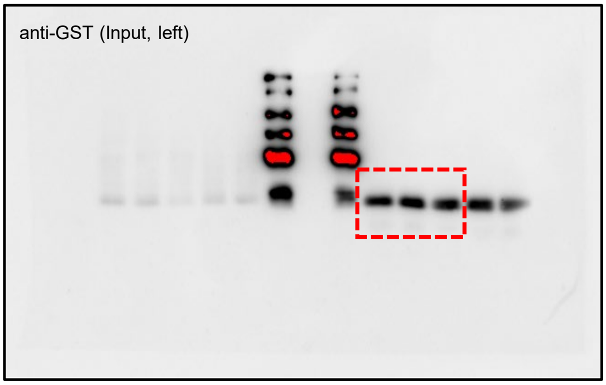

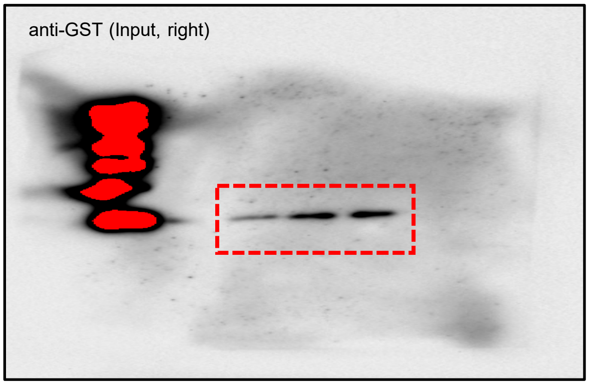

Supplementary Fig. 5d

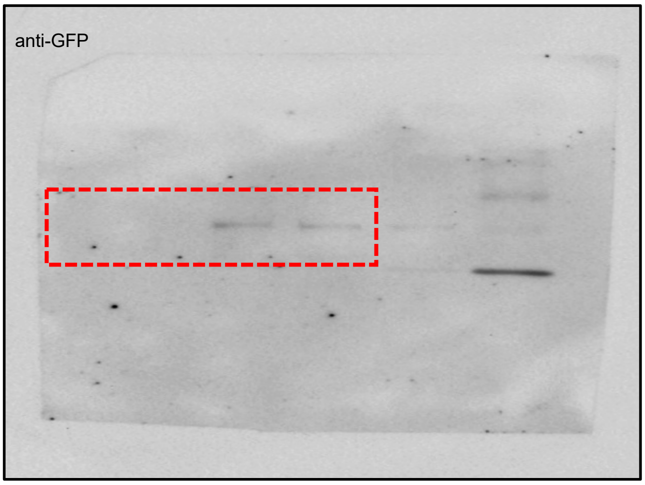

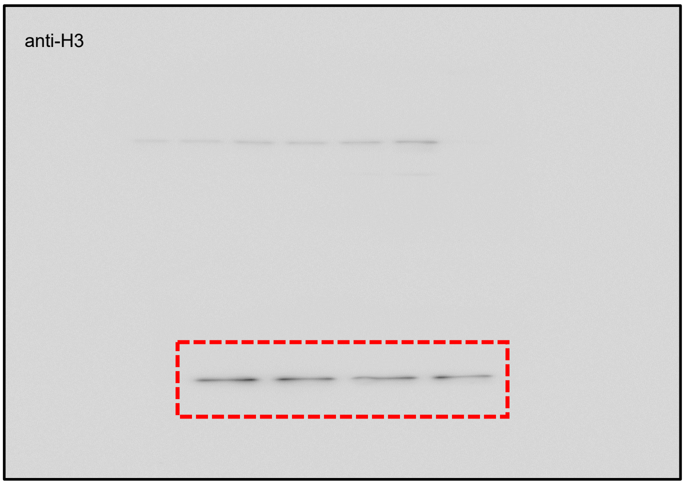

Supplementary Fig. 7a

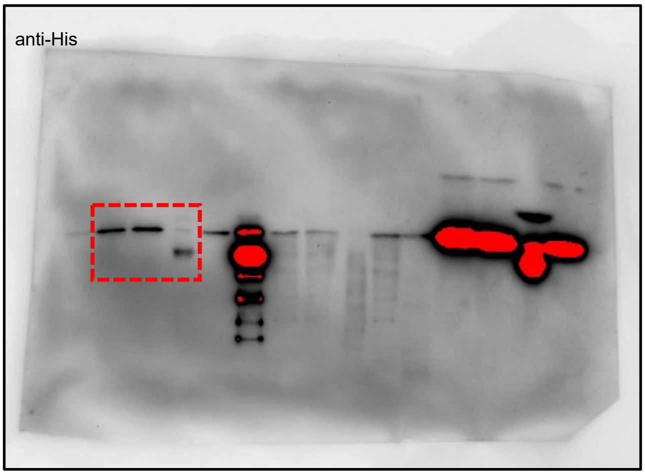

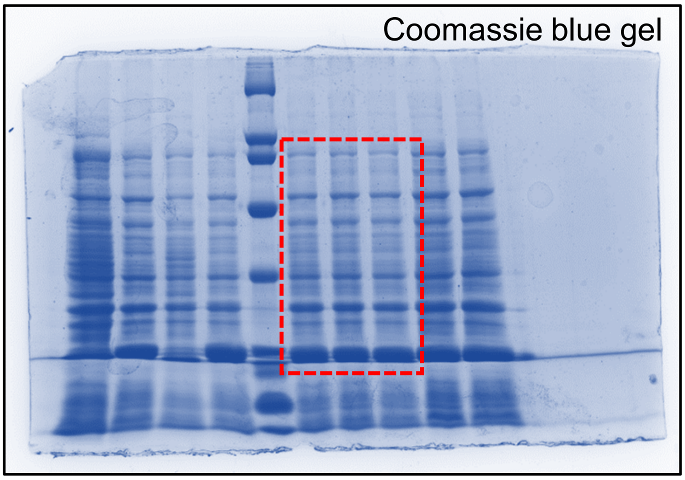

Supplement: Supplementary file 1 — Supplementary Figs. 1–7 and uncropped scans of blots and gels in the Supplementary Figs. 4b, 5b,d and 7a. [file 41477_2023_1605_MOESM1_ESM.pdf]
