# Supplementary material for: Regulation of micro- and small-exon retention and other splicing processes by GRP20 for flower development
Source: Nat Plants. 2024 Jan 9;10(1):66–85. doi: 10.1038/s41477-023-01605-8 (PMC10808074; doi:10.1038/s41477-023-01605-8)
Supplement: Supplementary file 2 — Reporting Summary [file 41477_2023_1605_MOESM2_ESM.pdf]

nature portfolio

Corresponding author(s): Hong Ma

Last updated by author(s): Nov 15, 2023

Reporting Summary

Nature Portfolio wishes to improve the reproducibility of the work that we publish. This form provides structure for consistency and transparency in reporting. For further information on Nature Portfolio policies, see our Editorial Policies and the Editorial Policy Checklist.

Statistics

For all statistical analyses, confirm that the following items are present in the figure legend, table legend, main text, or Methods section.

n/a	Confirmed	
☐	☒	The exact sample size (*n*) for each experimental group/condition, given as a discrete number and unit of measurement
☐	☒	A statement on whether measurements were taken from distinct samples or whether the same sample was measured repeatedly
☐	☒	The statistical test(s) used AND whether they are one- or two-sided<br>*Only common tests should be described solely by name; describe more complex techniques in the Methods section.*
☒	☐	A description of all covariates tested
☒	☐	A description of any assumptions or corrections, such as tests of normality and adjustment for multiple comparisons
☐	☒	A full description of the statistical parameters including central tendency (e.g. means) or other basic estimates (e.g. regression coefficient) AND variation (e.g. standard deviation) or associated estimates of uncertainty (e.g. confidence intervals)
☐	☒	For null hypothesis testing, the test statistic (e.g. *F*, *t*, *r*) with confidence intervals, effect sizes, degrees of freedom and *P* value noted<br>*Give P values as exact values whenever suitable.*
☒	☐	For Bayesian analysis, information on the choice of priors and Markov chain Monte Carlo settings
☒	☐	For hierarchical and complex designs, identification of the appropriate level for tests and full reporting of outcomes
☐	☒	Estimates of effect sizes (e.g. Cohen's *d*, Pearson's *r*), indicating how they were calculated

Our web collection on statistics for biologists contains articles on many of the points above.

Software and code

Policy information about availability of computer code

Data collection	Flower images were obtained by a Nikon microscope (SMZ-U) and AmScope Microscope Digital Camera (Cat#MU1803-HS, AmScope); SEM photographs were taken by a VP detector in Zeiss SIGMA VP-FESEM (Zeiss); confocal microscopes LSM880 (Zeiss) or FV1000 (Olympus) were used for the confocal imaging; Applied Biosystems (ABI) StepOnePlus real-time PCR systems (Cat#4376600, ThermoFisher) was used to collect qPCR raw data; RNA-seq raw data were obtained by Illumina NextSeq 2000 instrument with 2×150 bp paired-end outputs; the images of RNA EMSA and Western blot were obtained by a ChemiDoc image system (Bio-Rad).
Data analysis	catRAPID (http://s.tartaglialab.com/page/catrapid_group)<br>RNAbindPlus (http://ailab1.ist.psu.edu/RNABindRPlus/)<br>DRNApred (http://biomine.cs.vcu.edu/servers/DRNApred/)<br>PPRInt (https://webs.iiitd.edu.in/raghava/pprint/index.html)<br>ParSe (http://folding.chemistry.msstate.edu/utils/parse.html)<br>Phyre2 (http://www.sbg.bio.ic.ac.uk/phyre2/html/page.cgi?id=index)<br>Image J (v1.53t; https://imagej.nih.gov/i)<br>TopHat (v2.0.9; https://ccb.jhu.edu/software/top)<br>DESeq2 (v1.0; https://bioconductor.org/packages/release/bioc/html/DESeq2.html)<br>Gene Ontology (http://geneontology.org/)<br>Venny (v2.1.0; http://bioinfogp.cnb.csic.es/tools/venny/)<br>R (v3.5.2)<br>qPrimerDB (http://biodb.swu.edu.cn/qprimerdb)<br>Primer3Plus (https://www.primer3plus.com/)<br>Multivariate analysis of transcript splicing assay program (v4.1.1; http://rnaseq-mats.sourceforge.net/)

IGV (v2.9.4)
MEME program (v5.4.1); https://meme-suite.org/meme/tools/meme)
NEBaseChanger (https://nebasechanger.neb.com/
MEGA 7 (www.megasoftware.net)
Gene Structure Display Server (v2.0) (http://gsds.gao-lab.org/index.php)
TBtools (https://github.com/CJ-Chen/TBtools)
Photoshop CS5 (Adobe)
Illustrator CC (Adobe)
Phyre2 (http://www.sbg.bio.ic.ac.uk/phyre2/html/page.cgi?id=index)
Confocal software (Zeiss, LSM Zen v3.4)
Confocal software (Olympus, FV1000)
SEM software (Zeiss, SIGMA VP-FESEM)
GraphPad Prism 9 (https://www.graphpad.com/scientific-software/prism/)
The Microsoft Excel (Microsoft)
BioRender (https://biorender.com/)
AlphaFold (https://alphafold.ebi.ac.uk/)
Swiss model (https://swissmodel.expasy.org/)
Phytozome v13 (https://phytozome-next.jgi.doe.gov/)
Microsoft Excel 2013
Microsoft Powerpoint 2021
SUPPA2 (https://github.com/comprna/SUPPA)

For manuscripts utilizing custom algorithms or software that are central to the research but not yet described in published literature, software must be made available to editors and reviewers. We strongly encourage code deposition in a community repository (e.g. GitHub). See the Nature Portfolio guidelines for submitting code & software for further information.

Data

Policy information about availability of data

All manuscripts must include a data availability statement. This statement should provide the following information, where applicable:

- Accession codes, unique identifiers, or web links for publicly available datasets
- A description of any restrictions on data availability
- For clinical datasets or third party data, please ensure that the statement adheres to our policy

All data are available in the main text or Extended data. Raw data of RNA-seq of WT and grp20 floral and leaf transcriptomes have been deposited in the SRA database in NCBI with accession number PRJNA851744. The gene and protein information of Arabidopsis and other species were obtained from TAIR (https://www.arabidopsis.org/), UniProt (https://www.uniprot.org/) and Phytozome v13 (https://phytozome-next.jgi.doe.gov/). The prediction protein structures were obtained from AlphaFold Protein Structure Database (https://alphafold.ebi.ac.uk/).

Human research participants

Policy information about studies involving human research participants and Sex and Gender in Research.

Reporting on sex and gender	N/A
Population characteristics	N/A
Recruitment	N/A
Ethics oversight	N/A

Note that full information on the approval of the study protocol must also be provided in the manuscript.

Field-specific reporting

Please select the one below that is the best fit for your research. If you are not sure, read the appropriate sections before making your selection.

☒ Life sciences ☐ Behavioural & social sciences ☐ Ecological, evolutionary & environmental sciences

For a reference copy of the document with all sections, see nature.com/documents/nr-reporting-summary-flat.pdf

Life sciences study design

All studies must disclose on these points even when the disclosure is negative.

Sample size: For transgenic plants, six transgenic lines were characterized for each complementation/rescue experiments with wild-type or mutant constructs. qRT-PCR and RIP (RNA immunoprecipitation)-qRT-PCR experiments were performed with three biological replicates. Western blot and RNA EMSA experiments were conducted with three biological replicates. Statistics have been derived to calculate error bars. All error bars were defined as the standard deviation (SD) or standard error (SE) based on sample sizes indicated in the method and figure legends. The

two-tailed Student's t test with two-sided in 95% confidence intervals was used in the manuscript. Box plots were displayed with five values (lines) from top to bottom: maximum, third quartile, median, first quartile and minimum. The lower and upper whiskers are the minimum value and maximum value, respectively, without the outliers, which are observed data points statistically determined to be outside the upper and lower boundaries (whiskers).

Data exclusions

No data were excluded from the final analyses.

Replication

All analyses in the study have two or more biological replications. The numbers of biological replication were indicated in the corresponding figure legends.

Randomization

The materials were collected randomly from WT, mutant and transgenic plants. The clones for plasmid construction and protein expression were randomly selected.

Blinding

We did not perform analyses with blinding when preformed experiment and data analyses. However, the investigators were blinded to group allocation during data collection.

Reporting for specific materials, systems and methods

We require information from authors about some types of materials, experimental systems and methods used in many studies. Here, indicate whether each material, system or method listed is relevant to your study. If you are not sure if a list item applies to your research, read the appropriate section before selecting a response.

Materials & experimental systems

n/a	Involved in the study	
☐	☒	Antibodies
☒	☐	Eukaryotic cell lines
☒	☐	Palaeontology and archaeology
☒	☐	Animals and other organisms
☒	☐	Clinical data
☒	☐	Dual use research of concern

Methods

n/a	Involved in the study	
☒	☐	ChIP-seq
☒	☐	Flow cytometry
☒	☐	MRI-based neuroimaging

Antibodies

Antibodies used

anti-His antibody (mouse Ab, Cat#MA1-21315, ThermoFisher, 1:1000 dilution, clone name: HIS.H8);
anti-GST antibody(mouse Ab, Cat#AE001, ABclonal, 1:1000 dilution, clone name: AMC0501);
anti-GFP antibody(rabbit Ab, Cat#AE011, ABclonal, 1:1000 dilution, clone name: not provided by the manufacturer);
anti-GFP antibody(mouse Ab, Cat#AE012, ABclonal, 1:50, dilution, clone name: AMC0483R);
anti-FLAG antibody(mouse Ab, Cat#AE005, ABclonal, 1:1000 dilution, clone name: AMC0382);
anti-β-Tubulin antibody (rabbit Ab, Cat#AC008, ABclonal, 1:1000 dilution, clone name: not provided by the manufacturer);
anti-Histone 3 antibody (rabbit Ab, Cat#AS10710, Agrisera, 1:2000 dilution, clone name: not provided by the manufacturer);
goat anti-mouse HRP conjugated secondary antibody (Cat#62-6520, Invitrogen, 1:2000 dilution, RRID: AB_2533947);
goat anti-rabbit HRP conjugated secondary antibody (Cat#31460, Invitrogen, 1:2000, RRID: AB_228341).

Validation

anti-His antibody (mouse Ab, Cat#MA1-21315, validation in Western blot on untransfected HeLa cells and by transfecting HeLa cells with PTEN-pTT5 plasmid. The blot was probed with Anti-6x-His Tag Mouse Monoclonal Antibody (1:500 dilution));
anti-GST antibody (mouse Ab, Cat#AE001, validation in Western blot (Homo sapiens, Oryza sativa, Ctenopharyngodon idellus, Gallus gallus, Yeast, Other, Arabidopsis thaliana, Nicotiana benthamiana, Mus musculus, grass carp, Botrytis cinerea, Glycine max, Caenorhabditis elegans, Escherichia coli, Sus scrofa, Chlorocebus aethiops, Oryza sativa L, Malus pumila));
anti-GFP antibody(rabbit Ab, Cat#AE011, validation in Western blot (Arabidopsis thaliana, Homo sapiens, Mus musculus, Zea mays, lamprey, Nicotiana tabacum, Streptococcus pneumoniae, Glycine max, Solanum tuberosum , Cyprinus Carpio) and in Co-IP (Homo sapiens, Arabidopsis thaliana));
anti-GFP antibody(mouse Ab: Cat#AE012, validation in Western blot (Homo sapiens, Helicoverpa armigera, Saccharomyces cerevisiae, Arabidopsis thaliana, Ctenopharyngodon idellus, Nicotiana tabacum L, Rosa rugosa Thunb, Mus musculus, Yeast, Nicotiana tabacum L., Chlorocebus sabaeus, Other, Sus scrofa, N. benthamiana, Cotton bollworms, Populus, Solanum tuberosum, Oryza sativa, Ictalurus punctatus, Anatinae, N. benthamiana plants, Pyrus pyrifolia, Hordeum vulgare, Juvenile tilapia, G.hirsutum, Phytophthora capsici, N. tabacum , Nicotiana benthamiana, Danio rerio, N. tabacum , Carassius auratus gibelio, Caenorhabditis elegans, Solanum lycopersicum L, Cynoglossus robustus, Zea mays, tobacco, M. oryzae, Triticum aestivum, Chlorocebus aethiops, Rattus norvegicus) and in immunoprecipitation (Homo sapiens, Ctenopharyngodon idellus, Anatinae, Chlorocebus aethiops, Arabidopsis thaliana));
anti-FLAG antibody(mouse Ab, Cat#AE005, validation in Western blot (Homo sapiens, Mus musculus, Oryza sativa, Arabidopsis thaliana, Sus scrofa, Danio rerio, Drosophila melanogaster, Ctenopharyngodon idellus, Escherichia coli, Common carp, Xenopus laevis, Cyprinus carpio, Grapevine, Lycopersicon esculentum, D. melanogaste, G.hirsutum, Phytophthora capsici, N. benthamiana, Pimephales promelas, Nicotiana benthamiana, Human, Cherax quadricarinatus, Rattus norvegicus, Zea mays, Oryctolagus cuniculus, Saccharomyces cerevisiae, Ictalurus punctatus, Triticum aestivum) and in ChIP (Arabidopsis thaliana plants));
anti-β-Tubulin antibody (rabbit Ab, Cat#AC008, validation in Western blot (Mus musculus, Crassostrea gigas, Homo sapiens, Rattus norvegicus, Oncorhynchus mykiss, Brachyura, Capra hircus, Eriocheir sinensis H.Milne-Edwards, Dianthus caryophyllus L., Gallus gallus, Sus scrofa, blunt snout bream));
anti-Histone 3 antibody (rabbit Ab, Cat#AS10710, validation in Arabidopsis thaliana, Brassica oleracea, Capsicum annuum, Cicer arietinum L., Chlamydomonas acidophila, Chlamydomonas reinhardtii, Cucumis sativus L cv Suyo, Cucurbita pepo L. var. cylindrica,

Hordeum vulgare, human, Nicotiana benthamiana, Phalaenopsis equestris, Physcomitrium patens, Salicornia europaea, Solanum lycopersicum, Solanum sogarandinum, Solanum tuberosum, Vicia faba, Zea mays);
goat anti-mouse HRP conjugated secondary antibody (Cat#62-6520, validation in Western blot (whole cell extracts of T47D, A549, U2OS, K562, HeLa, Raji, HEK-MSR, MCF7, MDA-MB-231, LNCaP and Hep G2 cell lines);
goat anti-rabbit HRP conjugated secondary antibody (Cat#31460, validation in Western blot (whole cell extracts of A549 and Hep G2 cell lines).
The validation statement and complete species and experiment test of antibodies also can be found in the manufacturers' websites using catalogue numbers.
