# Supplementary figures and images for: Regulation of micro- and small-exon retention and other splicing processes by GRP20 for flower development
Source: Nat Plants. 2024 Jan 9;10(1):66–85. doi: 10.1038/s41477-023-01605-8 (PMC10808074; doi:10.1038/s41477-023-01605-8)

**Fig. 6c**

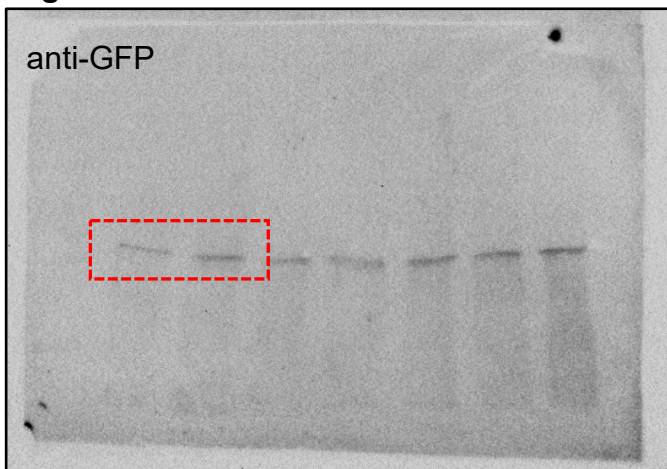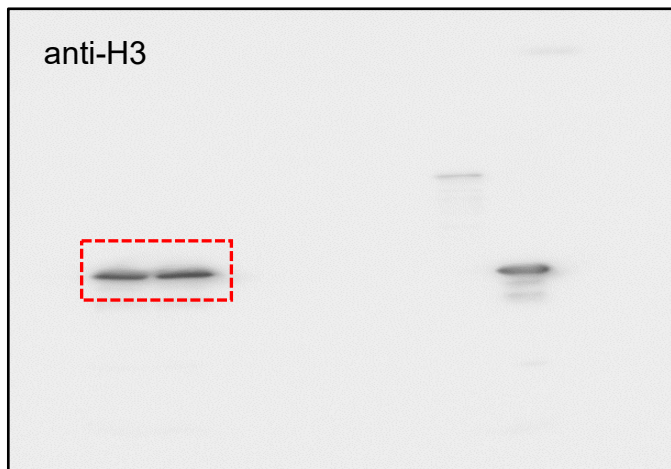

Supplement: Supplementary file 7 — Unprocessed western blots and gels for Fig. 6c. [file 41477_2023_1605_MOESM7_ESM.pdf]

**Fig. 7d**

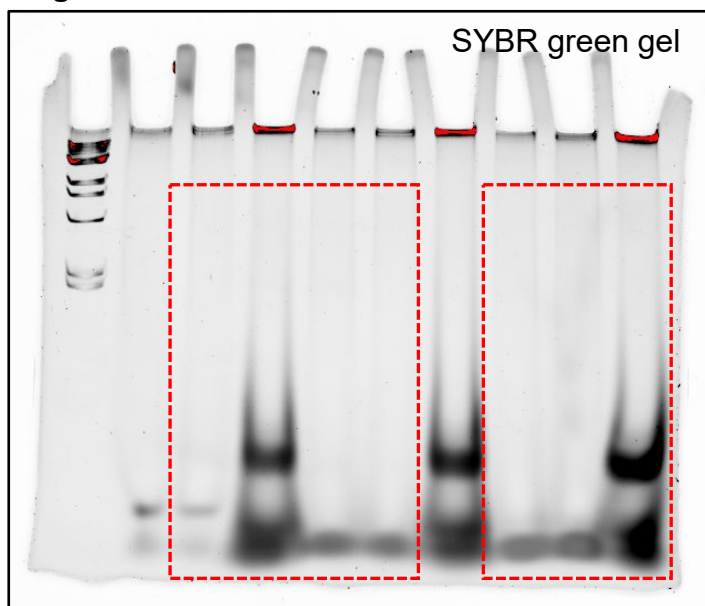

Supplement: Supplementary file 8 — Unprocessed western blots and gels for Fig. 7d. [file 41477_2023_1605_MOESM8_ESM.pdf]

**Fig. 8a**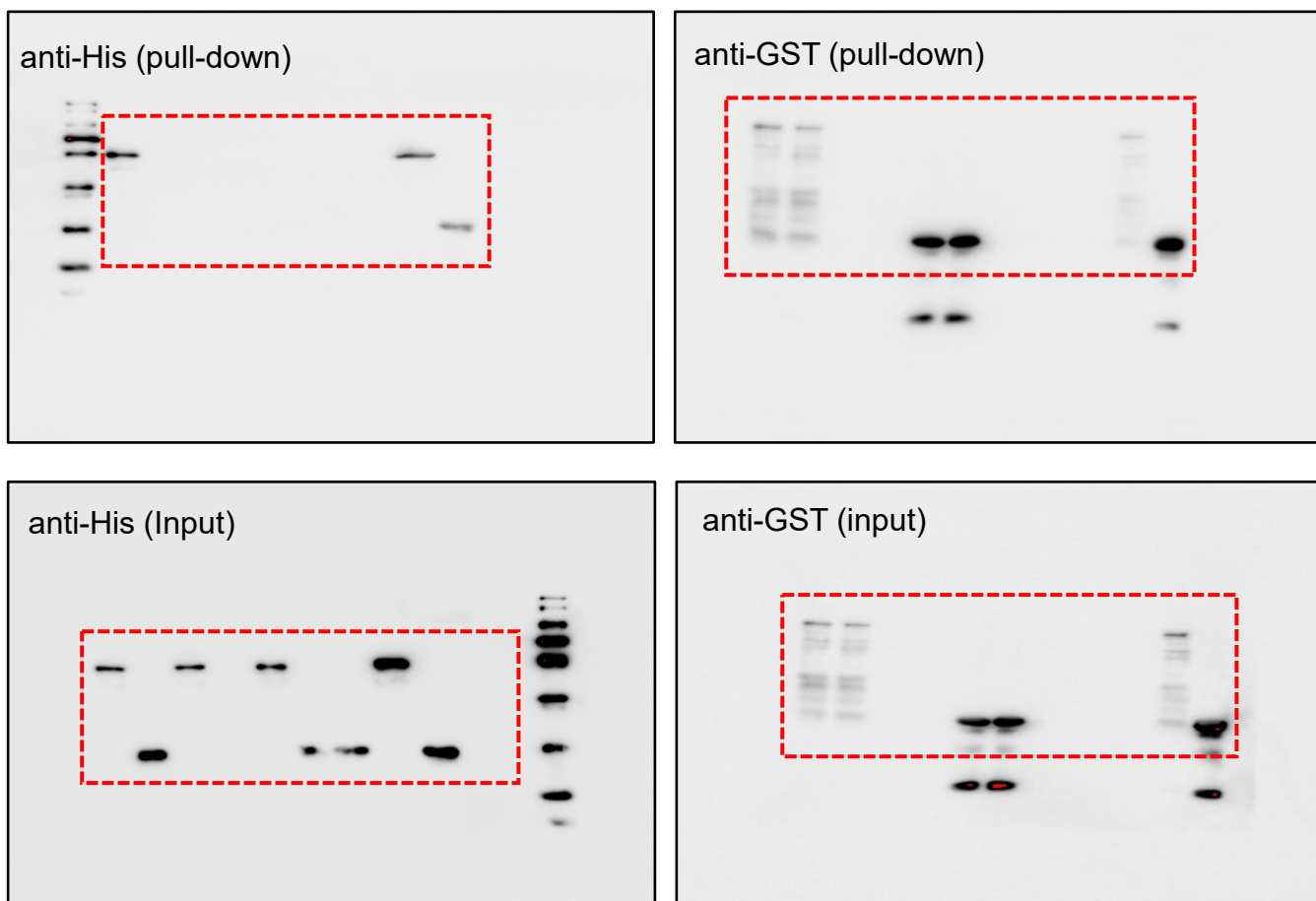

**Fig. 8c**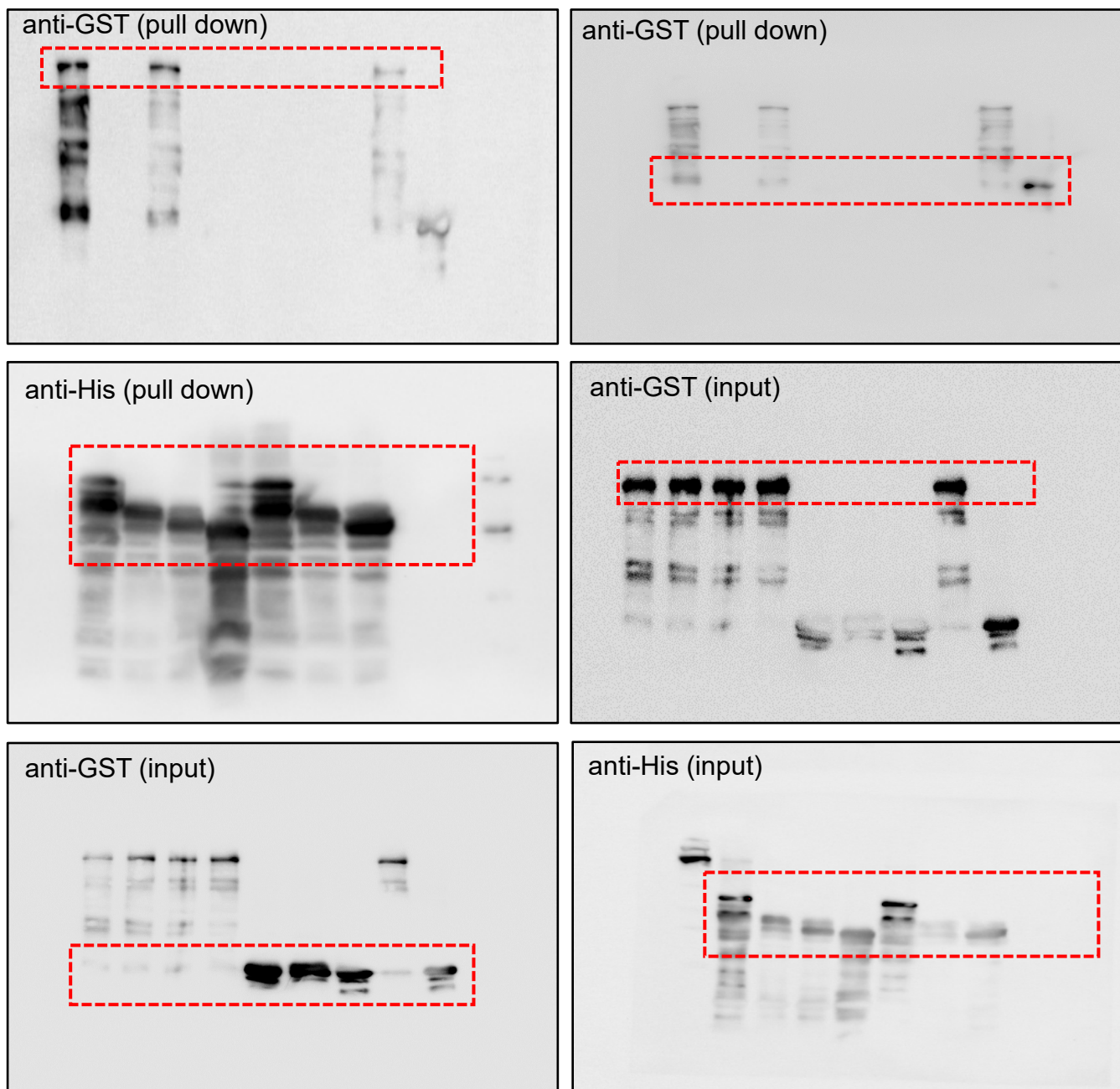

Supplement: Supplementary file 9 — Unprocessed western blots and gels for Fig. 8a,c. [file 41477_2023_1605_MOESM9_ESM.pdf]

**Extended Data Fig. 6h**

anti-GFP

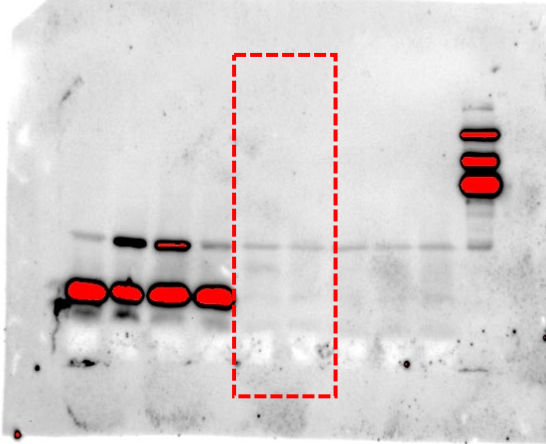

Supplement: Supplementary file 13 — Unprocessed western blots and gels for Extended Data Fig. 6h. [file 41477_2023_1605_MOESM13_ESM.pdf]

Extended Data Fig. 8g

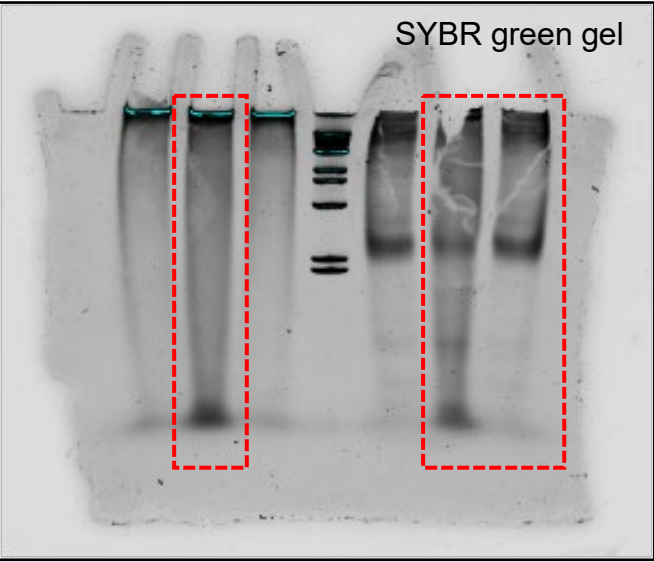

Unprocessed gels

Extended Data Fig. 8h

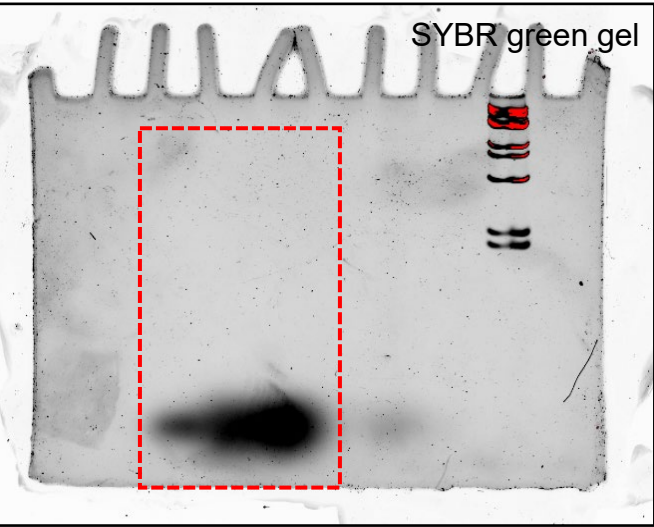

Extended Data Fig. 8j

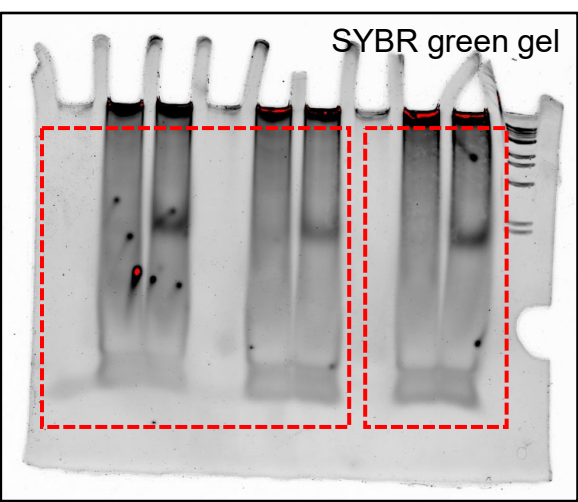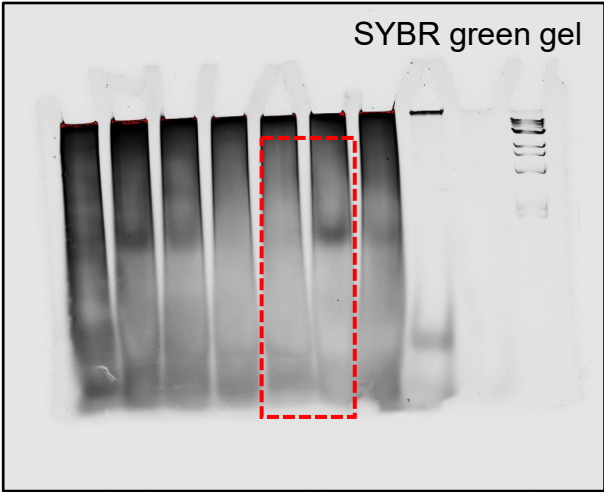

Supplement: Supplementary file 14 — Unprocessed western blots and gels for Extended Data Fig. 8g,h,j. [file 41477_2023_1605_MOESM14_ESM.pdf]

**Extended Data Fig. 9c**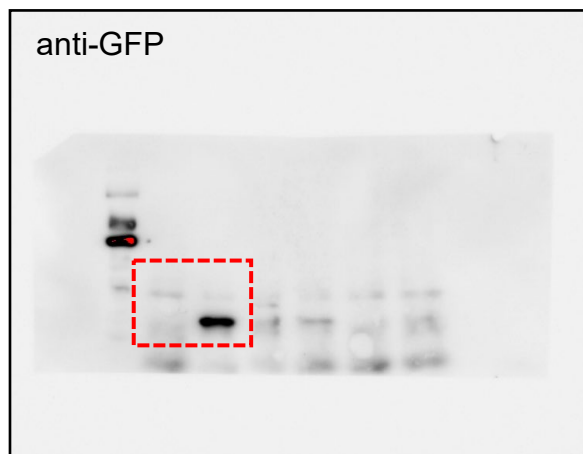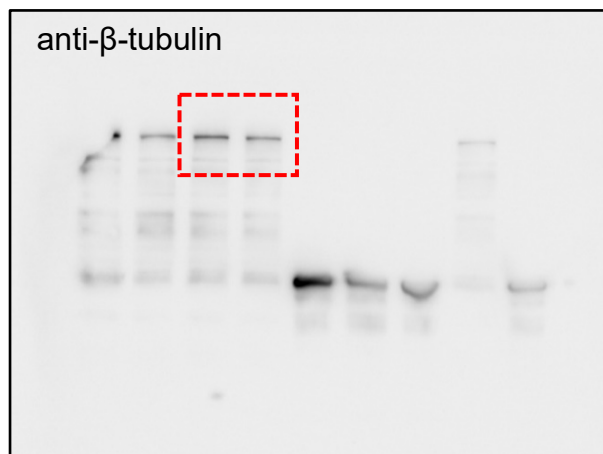

Supplement: Supplementary file 15 — Unprocessed western blots and gels for Extended Data Fig. 9c. [file 41477_2023_1605_MOESM15_ESM.pdf]

**Extended Data Fig. 10c**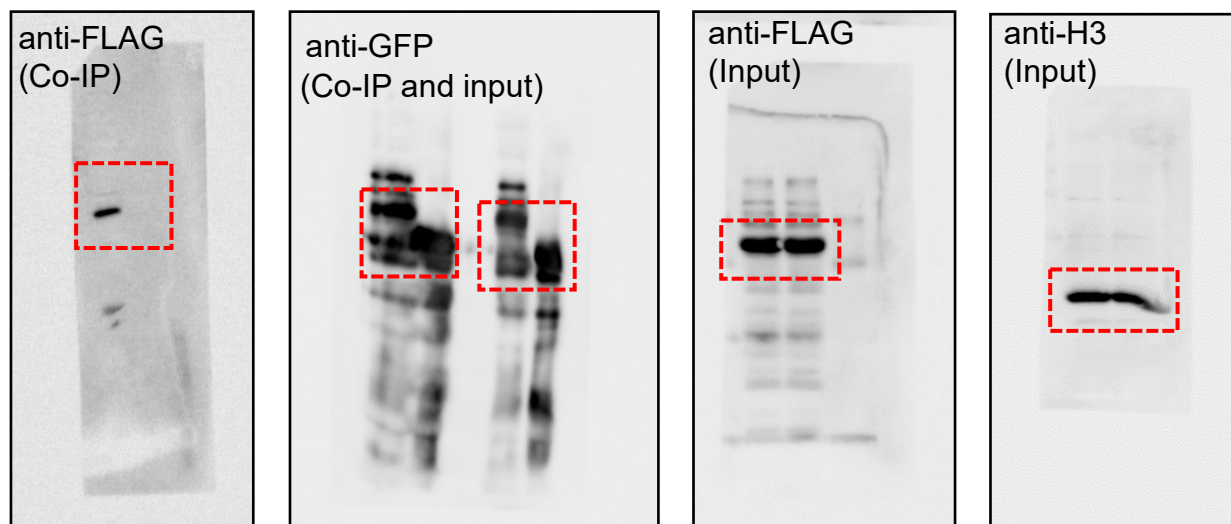

Supplement: Supplementary file 17 — Unprocessed western blots and gels for Extended Data Fig. 10c. [file 41477_2023_1605_MOESM17_ESM.pdf]
